# An Evidence Map of Pupillary Assessment in a Preselected Acute-Care Literature Archive

**DOI:** 10.3390/diagnostics16213698

**Published:** 2026-11-09

**Authors:** Cheng-Yueh Li, Chiao-Yin Cheng, Chih-Jung Chang, Hua Ho, Yao-Jen Liang, Jen-Tang Sun

**Affiliations:** 1 Department of Emergency Medicine, Far Eastern Memorial Hospital, New Taipei 220216, Taiwan; femh97069@femh.org.tw (C.-Y.L.); femhr9969@femh.org.tw (C.-Y.C.); femh88558@femh.org.tw (C.-J.C.); femh97657@femh.org.tw (H.H.); 2 Graduate Institute of Applied Science and Engineering, Fu Jen Catholic University, New Taipei 242062, Taiwan; 071558@mail.fju.edu.tw; 3 Department and Institute of Life Science, Fu Jen Catholic University, New Taipei 242062, Taiwan

**Keywords:** pupillary light reflex, quantitative pupillometry, acute care, neurocritical care, diagnostic assessment, prognosis, evidence mapping

## Abstract

**Background/Objectives:** We examined clinical purpose and acquisition method in a preselected acute-care archive of unknown representativeness. **Methods:** We reconciled 871 historical records using numerical adult-age evidence. Historical dual-human review, investigator-adopted dispositions for 325 unresolved records, a separate 100-record human-verified audit, and source reconciliation were distinguished. Six mapping domains and a scenario adding 274 age-unverified reports were analyzed. **Results:** The archive contained 355 core reports, 15 noncore records, 497 eligibility exclusions, three unresolved sources and one duplicate. Acquisition remained unestablished in 237/355 reports (66.8%): 38 remained unresolved after targeted source reconciliation, and 199 were not sampled for that acquisition audit. Any automated component was documented in 17/39 cardiac-arrest reports (43.6%), 15/27 mixed neurological/trauma reports (55.6%), and 6/116 trauma reports (5.2%). Diagnostic/physiological questions predominated in ocular and toxicological reports; neurological prognosis predominated after cardiac arrest. Adding age-unverified reports increased trauma representation from 32.7% to 39.9%. **Conclusions:** Clinical questions and documented acquisition profiles differ within this archive, while the numerical-age rule changes case mix. Unestablished acquisition and unequal verification limit comparisons of documented assessment methods across clinical groups. These findings do not establish field-wide coverage, clinical adoption, or comparative effectiveness.

## 1. Introduction

The pupil is both a readily observable clinical sign and a dynamic physiological signal. Its size, symmetry, reactivity, and response kinetics reflect interacting autonomic, ocular, and central nervous system processes. These features make pupillary examination useful at the bedside, but also make its interpretation dependent on the stimulus, ambient illumination, examination technique, medication exposure, and clinical context. Therefore, a binary description of an absent light response and a device-derived continuous parameter should not be treated as interchangeable measurements [1,2].

Pupillary assessment spans several distinct clinical questions. In emergency care, a new abnormality may contribute to recognition of neurological injury, ocular pathology, or drug exposure. In neurocritical care, serial measurements may accompany physiological monitoring or contribute to outcome assessment. Manual examination is widely recognized, while automated devices provide standardized stimulation and quantitative readouts; nevertheless, a numerical output does not by itself establish diagnostic validity or clinical utility. Studies comparing bedside examinations with instrumented assessment illustrate why acquisition technique and the target question must be recorded explicitly [3,4,5,6].

Recent multicenter and cohort investigations demonstrate the breadth of this evidence. The ORANGE study enrolled 514 patients with acute brain injury, with six-month outcomes available for 497 [7]. Miroz and colleagues investigated early quantitative pupillometry in 100 patients receiving venoarterial extracorporeal membrane oxygenation [8]. These populations are not interchangeable with one another or with a general emergency-department population. Contemporary post-cardiac-arrest guidance places pupillary findings within multimodal prognostication rather than treating an isolated pupil measurement as a universal decision rule [9,10].

Evidence mapping organizes reports by clinical context, measurement approach, and intended outcome, whereas scoping reviews use systematic and transparent methods to characterize a defined body of literature [11,12,13,14]. We adapted the descriptive mapping function to a fixed historical archive; this is not equivalent to a comprehensive scoping review. Because the original collection history is only partially recoverable, an empty cell in this archive cannot establish that the wider literature contains no relevant research.

Therefore, we analyzed a fixed historical collection as a secondary, report-level dataset. The contribution is a source-linked comparison of clinical questions, acquisition evidence, and the consequences of a strict adult-evidence rule within that collection. We did not design this analysis to rank field-wide evidence gaps. The six-domain map separates verified information from unresolved extraction and preserves an auditable route from each count to its report.

## 2. Materials and Methods

### 2.1. Design, Registration, and Unit of Analysis

This descriptive secondary analysis uses reports, not independent cohorts, as its unit. PROSPERO CRD420261441918 was first published on 5 July 2026. The supplied Version 1.0 includes adult eligibility and evidence mapping by phenotype, cause, assessment, setting, and outcome; it does not prescribe the final six-domain taxonomy or the later numerical-documentation rule. Therefore, these operational refinements are distinguished from the original protocol. The supplied registration PDF does not establish publication of a later amendment. Reporting was informed by PRISMA-ScR and PRISMA-S, with a PRISMA 2020 applicability crosswalk rather than a claim of a newly completed field-wide review [15,16,17]. The scope is specified in Supplementary Table S1; the supplementary methods separately itemize protocol-to-analysis differences.

The analysis frame was the preselected 871-record historical archive, dated 7 July 2026 by the investigator. Documentation did not independently separate its establishment from final locking or explain the original closure decision. Retaining a fixed frame allowed reconciliation of report identities, eligibility, and classifications without presenting incompletely processed external candidates as a completed update. This is the rationale for the present secondary analysis, not an explanation reconstructed for the original selection process. The underlying disposition version was assembled on 12 October 2026; the present revision preserves all 871 identifiers, with versioned changes to two eligibility decisions after further source-evidence review on 30 October 2026.

### 2.2. Search Provenance and Archive Boundaries

The preserved PubMed execution log is dated 5 July 2026. Five modules, run through NCBI Entrez E-utilities without date, language, age, human, or publication-type filters, yielded 8953 record appearances and 7440 distinct PubMed identifiers; 1513 appearances were repeated across modules. These are search-operation counts, not the denominator from which the 355 core reports can be reconstructed. The investigator-reported last search date was 17 July 2026, after the archive date. Later search records could link back to the archive but were not automatically added to its closed frame. Original exports, queries, and duplicate-review materials are preserved separately from retrospective corrections.

The historical retrieval ledger contains 1239 records: 871 mapped records, 166 historical exclusions, and 202 retrieval failures. Its link to the original database-selection process remains incomplete. Two external PubMed candidate lists each contain 335 records, but share 315; their union is 355, comprising 97 historical exclusions, 156 historical retrieval failures, and 102 records without a ledger link. None belong to the 871-record frame. Thus, these lists and the 202 retrieval failures cannot be added as disjoint sets. Supplementary-source deduplication, metadata recovery, retrieval, and integration also remain incomplete. Embase was not searched, and no formal peer review of the search was documented. Exact module counts, candidate crosswalks, and processing status are provided in Supplementary Table S2.

A retrospective PubMed syntax audit on 28 October 2026 imposed a publication-date cutoff of 17 July 2026. The original syntax retrieved 7493 unique identifiers in the current database; canonical MeSH corrections retrieved 7534, and adding brain/cerebral herniation title/abstract terms retrieved 7536. The controlled changes therefore added 41 plus 2 candidates, without removing original-current results. These 43 candidates were not integrated into the map. This was not a replay of July indexing. Four of five post hoc illustrative known articles were retrieved; Meeker 2005 was missed because the applicable module required an outcome/prognosis block that the article did not satisfy. The test identifies a limitation, not a validated search sensitivity (Supplementary Table S2).

### 2.3. Eligibility and Disposition Rules

Eligible evidence concerned adults aged at least 18 years in acute illness, injury, exposure, new or otherwise unexplained pupillary abnormalities, or prespecified clinically relevant diagnostic-mimic contexts. Adult eligibility required numerical evidence, such as an explicit lower age limit, a reported age range confined to adults, individually identifiable adult observations, or numeric eligibility in a study registry explicitly linked by the article. Registry eligibility documents the intended population, not the observed minimum age. The word adult, a mean or median age, and an interquartile range alone did not satisfy this documentary rule. Where a source included minors, only a separately identifiable, complete baseline-defined adult subgroup could support restricted inclusion. Such a subgroup could be recovered from individual rows or uniquely determined cross-tabulations without requiring a separately published adult aggregate. A subset selected by survival or another subsequent outcome could not rescue eligibility. This rule concerns a post hoc rescue subset within a report whose full population otherwise lacks adult evidence; it does not exclude an originally defined report population solely because that study selected participants by outcome. Such a report can contribute only its documented adult population and design-appropriate descriptive findings, not the size or risk estimates of an unverified parent cohort.

Original reports required sufficient pupil observations or pupil-related results for their stated use. Reviews, editorials, and protocol-only reports were ineligible as original evidence. Single cases and anecdotal series were generally excluded; selected rare-condition descriptive series were retained outside the core. The 15 noncore records comprise 14 descriptive series and one BOX preliminary abstract (FT-1174), retained only to link it to the full report FT-0693. FT-1174 specifies age, cohort size, outcome, and quantitative results; the sibling abstract FT-1147 lacks information needed for independent eligibility and outcome interpretation. The protocol excludes abstracts with insufficient extractable data, rather than all abstracts. Neither abstract contributes an independent core count; the source comparison is provided in Supplementary Table S3.

Reports lacking verifiable numerical adult evidence were not included in this round and were retained on a reversible evidence-recovery list. This disposition did not imply that children were known to have been enrolled. Inseparable mixed-age evidence was treated separately. Delayed or chronic assessments without separable acute adult data were outside the main scope. Healthy-volunteer studies were generally outside scope unless they addressed a prespecified benign pupillary variation or pharmacological diagnostic mimic.

A missing or incorrect complete source was classified as a retrieval problem, not an eligibility failure. Clear publication-type exclusions could be supported by unambiguous source material without possession of every page. Retrieval was stopped at the investigator’s request for FT-0609, FT-0643, and FT-0897, two incomplete articles and one incorrect article, after the available records were reviewed. This post hoc, report-specific decision was not a prespecified age-of-publication cutoff and may select against older evidence. These three records remain separate from eligibility exclusions. Restricted adult-subgroup findings were not replaced by whole-cohort results; patients, eyes, model nodes, and repeated readings were distinguished. Outcome-selected subsets could not establish baseline adult eligibility, and outcome-optimized model nodes were not treated as independently validated adult test performance (Supplementary Table S3).

### 2.4. Human Review and Subsequent Source Verification

Chiao-Yin Cheng and Hua Ho independently performed the original screening, extraction, and mapping. Disagreements and unclear assignments were resolved through joint discussion involving both reviewers and Cheng-Yueh Li. This was a three-person consensus process, not an independent third-reviewer assessment. The authors confirmed this allocation of responsibilities for the present revision; historical reviewer-number fields alone do not identify individuals. That workflow is distinguished from the later source-verification batch: report-specific dispositions for 325 previously unresolved records were adopted by the investigator. Batch membership is defined by the structured adjudication object, not a free-text marker. Adoption does not itself document another independent rereading of each source; agreement estimates from a different frame are not reused for the present decisions.

Article identity was checked against title, authors, year, identifiers, and article-header or front-page evidence; an identifier appearing only in references was insufficient. Fifty of 273 EX07 records and 50 of 251 core records with initially unestablished acquisition were sampled without replacement using seed 20261028. Chiao-Yin Cheng prepared and passed the forms to Jen-Tang Sun for source-based judgment; Claude assisted with provisional form preparation. Sun was separate from the original screening and consensus decision-makers. The authors confirmed that the 43/50 age result was recorded after Sun’s first-pass source judgment and confirmation, before later comparison and reconciliation. The template omitted prior categories and reasons, but the records do not establish whether Sun could access those decisions during his first pass; a blinded human assessment is not claimed. The form dated 29 October 2026 preserves seven age discrepancies and 16 proposed manual classifications. Codex assisted subsequent source comparisons; Sun determined the reconciled human dispositions. This was reconciliation by the second reviewer, not another independent third review. Ancillary acquisition entries in the EX07 stratum did not replace sensitivity-map classifications.

The 30 October eligibility exercise examined preserved source-evidence records for all 239 reports then labeled acquisition unestablished. Codex assisted source-text comparison, consistency checking and preparation of technical eligibility recommendations. Twenty-one original PDFs were reopened for targeted age, subgroup or population questions; 218 records were checked using preserved source evidence. File identity and hashes were checked for all 239. Jen-Tang Sun independently verified the source-grounded results and gave final human confirmation of the 239 eligibility dispositions, as clarified by the authors for this revision. The tools were not reviewers and did not provide final decisions. This workflow does not establish that 239 full PDFs were newly read or that their acquisition methods were all re-audited. Unestablished acquisition was not itself an exclusion criterion.

### 2.5. Mapping Domains and Interpretive Safeguards

Each core report received one canonical category in six domains: etiology, assessment method, care setting, primary pupil-related endpoint, design, and publication period. Endpoint classification follows the primary pupil-related aim supported by Methods and Results. Agreement, inter-rater reliability, repeatability, and misclassification against another pupil-measurement method are methodological endpoints; diagnosis/acute physiology requires a clinical target condition, diagnostic question, or physiological association beyond measurement agreement. For example, device agreement in FT-0223 and nurse/device comparisons in FT-0296 and FT-0299 are methodological, even where mortality or management changes are mentioned secondarily. In contrast, FT-0080 evaluates detection of a relative afferent pupillary defect against a blinded ophthalmologist’s examination and is diagnostic. Sources with genuinely co-primary pupil-related aims enter the co-primary/multiple category once, not several endpoint cells. Treatment names in titles and the mere presence of secondary outcomes do not determine the category. Worked examples and source pages appear in Supplementary Table S3.

Assessment categories were manual, automated, manual plus automated, other instrumented, other mixed methods, and not established. Manual required a described clinical pupil observation or light-response test linked to patient assessment; a named examiner or light-source brand was not mandatory. A pupil category, a generic examination heading, or absence of a device name alone was insufficient. Thus, FT-0514 was classified as manual from the described clinical light-stimulation test, whereas FT-0773 remained unestablished after checking its linked AMPAS sources. Instrumented photography with manual grading was classified by its actual acquisition and grading process. Manual and automated indicate documented methods, not their exclusive use; nonexclusive component indicators were retained. Direct manual-automated comparison required within-study comparative data in eligible adult evidence. Differently timed prognostic comparisons were distinguished from synchronous agreement studies; use of both methods alone was insufficient. Not established was retained when the examined sources did not establish acquisition. The audit distinguished missing description in the materials examined, ambiguous evidence, and unavailable supporting material rather than treating all uncertainty as deficient original reporting.

Care setting concerned the relevant assessment, not author affiliation. A hospital report without a verified unit was labeled Hospital, unit not established; other unresolved settings were labeled Not established. These labels are used consistently in the main tables, current canonical data, and report index. Original extraction wording and historical decisions remain preserved in the audit record. Potentially overlapping reports were not summed into an independent patient denominator.

### 2.6. Descriptive Analysis

Core counts used 355 reports, with 15 noncore records indexed separately. Cross-tabulations compared etiology with acquisition and endpoints. A descriptive sensitivity scenario added all 274 EX07 reports to produce a 629-record denominator and repeated all six domains. Updated core classifications and retained EX07 classifications were used; the targeted EX07 age audit did not re-adjudicate acquisition across that stratum. The newly excluded FT-0120 enters this EX07 scenario, whereas the inseparable mixed-age report UF-43BA3EE5 does not. This scenario tests the compositional effect of relaxing the numerical-age documentation rule; it neither establishes adult eligibility nor resolves other source limitations. Periods were before 2000, 2000–2009, 2010–2019, and 2020–2026, with no annualization. Percentages were rounded to one decimal place. Audit confirmation and reclassification proportions used Wilson 95% confidence intervals, interpreted only within their sampled strata. Acquisition verification coverage was analyzed separately from eligibility. The 237 current unestablished labels were partitioned into 38 sampled and reconciled reports and 199 reports not sampled in the acquisition audit. A conditional sensitivity calculation held the 38 observed unresolved sampled reports fixed and applied q = 38/50 to the 199 unsampled reports: (38 + 199q)/355. Substitution of the Wilson limits for q gives a scenario range, not a confidence interval for the entire core or a new classification. It assumes comparable verification yield in sampled and unsampled retained reports, treats reconciled outcomes as fixed, and does not account for errors outside that sampling stratum. No disease-specific extrapolation was made.

Integer counts and audit intervals were calculated in Python. The coded report-level inputs, compact audit records, runtime information, and executable reproduction code are provided for publication as Supplementary Data File S1. The underlying original review forms and retained historical source records are available on reasonable request as specified in the Data Availability Statement. Figures and supplementary PDFs were generated from the data, retaining the Diagnostics Word template. No patient-level total, meta-analysis, or pooled accuracy was calculated. Cohen’s kappa was not used as overall reliability because the targeted strata were selected for a single baseline decision or category. Formal risk-of-bias and certainty assessments were not undertaken across this heterogeneous map; counts do not quantify evidence strength.

## 3. Results

### 3.1. Archive Disposition

**Figure 1:**
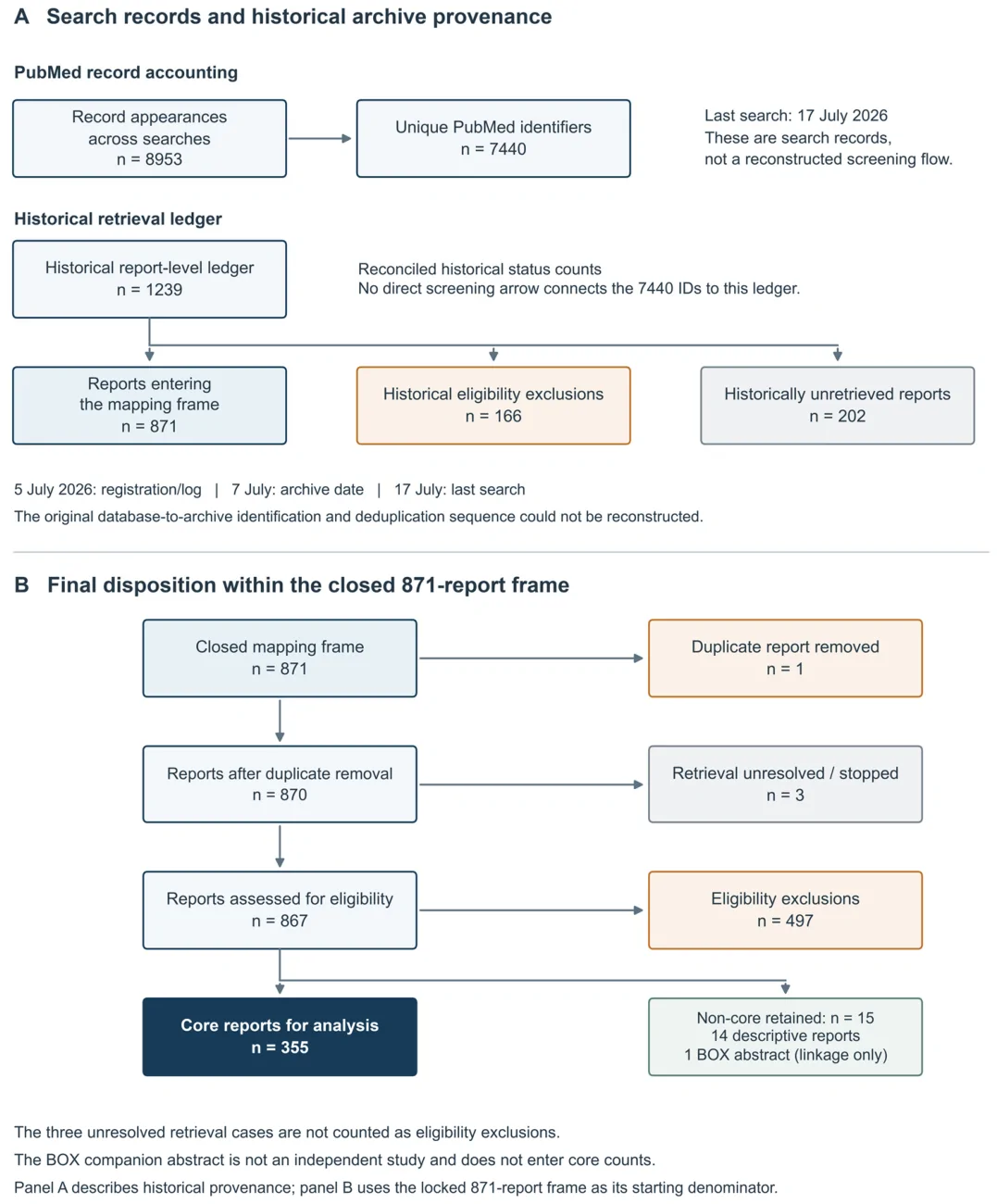
Search provenance and closed-archive disposition. Panel (**A**) distinguishes search identifiers from the historical retrieval ledger; the connecting selection pathway is not reconstructable. Panel (**B**) follows the fixed 871-record frame, keeping duplicate removal, unresolved retrieval, eligibility exclusions, core inclusions, and noncore retention separate. The 15 noncore records are 14 descriptive series and one linkage-only companion abstract.

One duplicate alias was removed from 871 historical records. Of 870 remaining records, three lacked a correct complete source at retrieval closure; 867 had source-supported dispositions. These comprised 497 eligibility exclusions, 355 core reports, and 15 noncore records: 14 descriptive series and one linkage-only BOX abstract. The additional eligibility review moved FT-0120 and UF-43BA3EE5 from the core to exclusions. Figure 1 separates search provenance from this eligibility flow rather than implying a continuous database-to-inclusion pathway (Figure 1; Supplementary Table S4). Newly supplied main articles and unresolved sources are distinguished in Supplementary Table S5, and the noncore inventory is given in Supplementary Table S6.

### 3.2. Clinical Contexts and Acquisition Methods

Trauma accounted for 116 core reports (32.7%), cerebrovascular disease for 74 (20.8%), and cardiac arrest for 39 (11.0%). Ocular/neuro-ophthalmic and toxicological/pharmacological contexts contributed 32 and 29 reports, respectively. These are report-level clinical contexts, not frequencies of causes among patients (Table 1).

The ICU was verified for 93 reports (26.2%) and the emergency department for 66 (18.6%). Multiple settings occurred in 25 reports, ophthalmic care in 20, and prehospital/community care in 10. The hospital unit was unestablished in 120 reports, and the setting was otherwise unestablished in five (Table 1).

Assessment was classified as automated in 53 reports (14.9%), manual in 42 (11.8%), and manual plus automated in 16 (4.5%). Four reports used other mixed methods and three other instrumented methods. Any automated component occurred in 71 reports (20.0%), including two other mixed-method reports: 17/39 cardiac-arrest reports (43.6%), 15/27 mixed neurological/trauma reports (55.6%), and 6/116 trauma reports (5.2%). Of 237 unestablished labels (66.8%), 38 reports (10.7% of the core) remained unresolved after sampled source reconciliation, and 199 (56.1%) were not sampled in that acquisition audit. Therefore, their verification coverage is unequal, including within the 106 trauma reports carrying that label. Under the conditional calculation, the unresolved share would be 53.3% (sensitivity range 45.8–58.7%). The observed ledger count remains 237; neither it nor the scenario estimates clinical adoption (Table 2; Supplementary Table S9f).

### 3.3. Pupil-Related Endpoints and Study Designs

Diagnosis/acute physiology was primary in 122 reports (34.4%), neurological outcome in 87 (24.5%), mortality in 67 (18.9%), and co-primary/multiple endpoints in 42 (11.8%). Management/disposition and other clinical outcomes each accounted for 16 reports; methodological endpoints accounted for five. Retrospective analyses were the largest design group (162), followed by prospective observational studies (64), secondary analyses (42), and case series (32). The complete endpoint and design distributions are provided in Supplementary Table S7.

Diagnostic/physiological questions predominated in ocular/neuro-ophthalmic reports (21/32, 65.6%) and toxicological/pharmacological reports (23/29, 79.3%). Neurological prognosis predominated after cardiac arrest (27/39, 69.2%). Thus, the same broad assessment domain served substantially different clinical questions across contexts; each report contributes to only one primary endpoint cell (Table 3).

### 3.4. Publication Periods and Age-Rule Sensitivity

**Figure 2:**
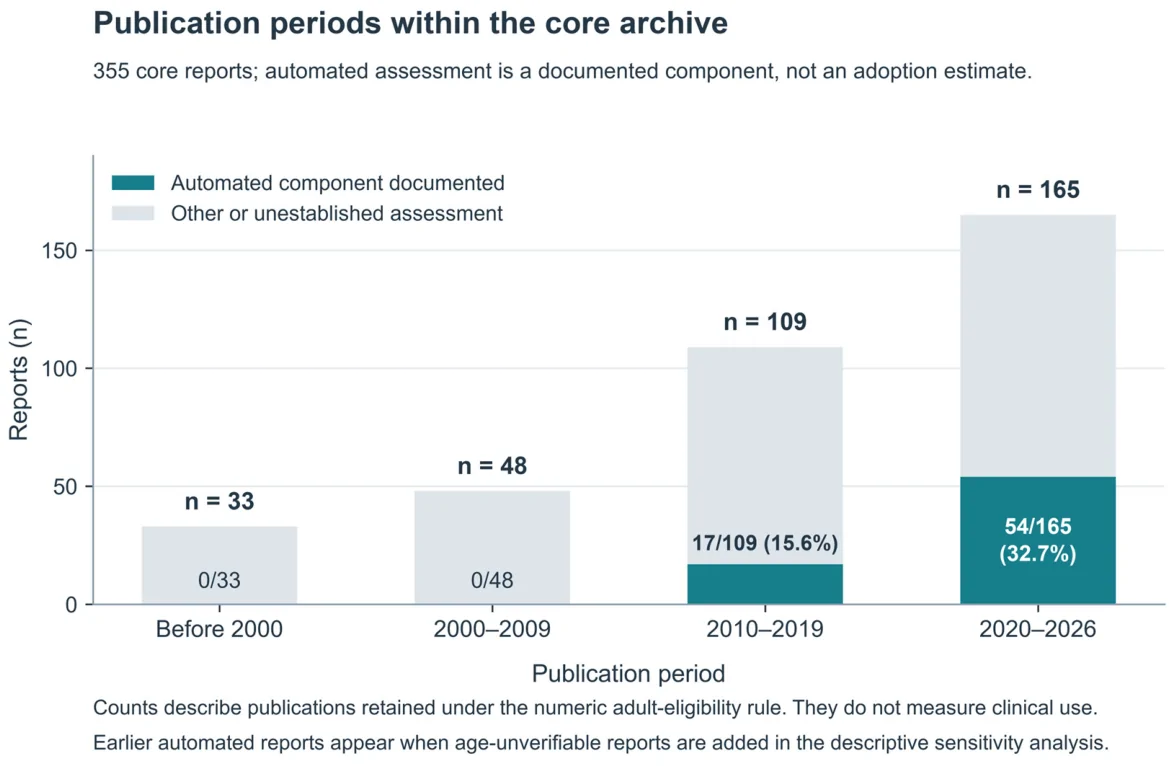
Publication periods in the core archive (*n* = 355). Automated-component reports are a subset of each total. Unequal periods are not annualized; counts do not estimate clinical adoption.

The core reports spanned 1981–2026; 165/355 (46.5%) were published during 2020–2026. Reports with any verified automated component occurred in 2010–2019 (17) and 2020–2026 (54), with the earliest core example in 2014. Acquisition remained unestablished in 99 of the 165 latest-period reports (Figure 2).

**Figure 3:**
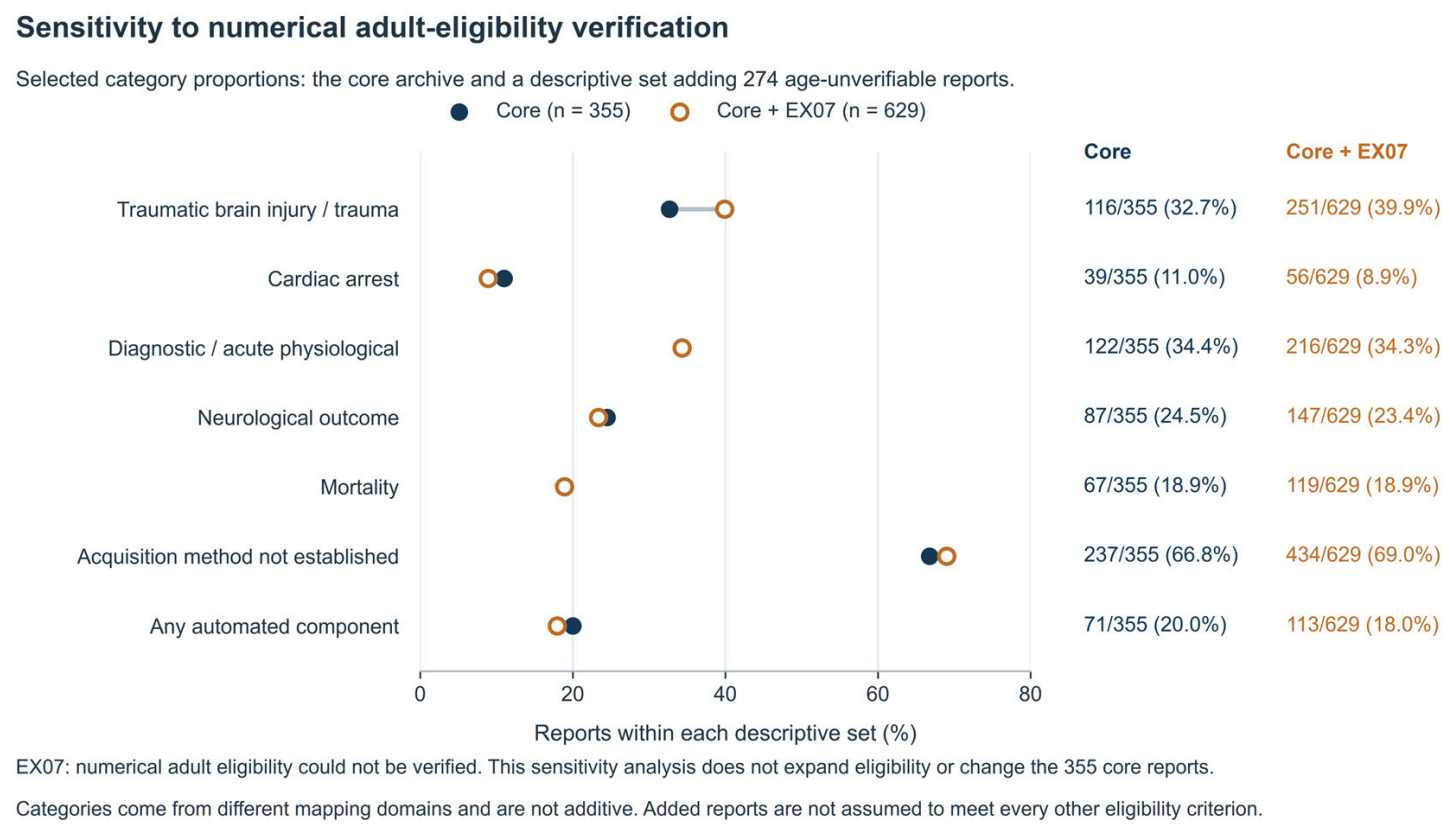
Descriptive sensitivity to the numerical-age documentation rule. The core map (*n* = 355) is compared with a hypothetical core-plus-EX07 scenario (*n* = 629). EX07 reports remain excluded from the primary map; this scenario does not establish adult eligibility. Acquisition labels combine sampled source reconciliation and retained classifications, rather than uniform verification. This age-rule comparison is distinct from the acquisition-verification scenario in S9f. Six-domain distributions and both acquisition matrices are provided in Supplementary Table S7.

In the 629-record sensitivity scenario, trauma representation increased from 116/355 (32.7%) to 251/629 (39.9%), whereas cardiac arrest decreased from 39/355 (11.0%) to 56/629 (8.9%). The proportion carrying an unestablished-acquisition label increased from 237/355 (66.8%) to 434/629 (69.0%); these totals combine different levels of verification; the diagnostic/physiological share was 34.4% versus 34.3%. Any automated component decreased from 20.0% to 18.0%. The higher verified automated representation in cardiac arrest and mixed neurological/trauma than in trauma persisted (41.1%, 55.8%, and 5.2%, respectively). However, the earliest automated record shifted to 1989 (FT-1083), so the core archive’s apparent post-2010 onset was not robust to the age rule. The full combined-frame etiology-by-acquisition matrix, including component flags, is provided in Supplementary Table S7b2 (Figure 3; Supplementary Table S7).

Twelve core reports had a documented direct manual-automated comparison (12/355, 3.4%). These included measurement-agreement studies and prognostic comparisons; they were not all synchronous paired tests. Their comparison type, timing, adult-population scope, and source locations are retained individually (Supplementary Table S7). Remaining numerical-extraction questions in FT-0671 and FT-0691 are listed separately from their completed mapping dispositions; no unresolved effect estimate was pooled in this study (Supplementary Table S8).

### 3.5. Targeted Audit and Source Reconciliation

All 50 sampled EX07 records retained the judgment that numerical adult eligibility could not be verified (100%; Wilson 95% CI 92.9–100%). Within the 50-report acquisition stratum, the first-pass judgments confirmed by Jen-Tang Sun before reconciliation supported adult eligibility in 43/50 (86.0%; 95% CI 73.8–93.0%), classified five as ineligible, and left two unverifiable. These raw judgments are not replaced by the later reconciled results. For acquisition, 34/50 retained the initial unestablished label and 16 proposed manual assessment. Reconciliation accepted 12/50 as manual (24.0%; 95% CI 14.3–37.4%) and retained 38 as unestablished. This reduced the original 251 unestablished labels to 239 before the subsequent two eligibility exclusions (Supplementary Table S9).

Jen-Tang Sun resolved the seven age discrepancies using complete baseline-defined adult subgroups in six reports and explicitly linked registry eligibility in one; all seven core inclusions were retained. He accepted 12 of the 16 proposed manual classifications and retained uncertainty in four. FT-0514 contributed its complete five-person adult subgroup, not only two deaths; its pupil denominator remained incompletely described. FT-0773 remained unestablished for acquisition because nonstandardized assessment and a registry pupil field did not identify the observation method. The raw return, evidence used in reconciliation, and final restrictions are separately recorded in Supplementary Table S9. Reconciled agreement is not substituted for the original 43/50 age result.

The further 239-record eligibility audit retained 237 reports: 191 on evidence for the report population and 46 with restricted, separately identifiable adult evidence. FT-0120 was excluded because a mean age for 122 ketamine-intoxication presentations did not establish numerical adult eligibility; the seven patients subsequently admitted to intensive care could not provide a baseline adult rescue subgroup (EX07). UF-43BA3EE5 was excluded because its naloxone responders included ages 14–83 and the adult nonresponders were selected by post-treatment response, not a separable baseline adult population (EX02). The audit therefore reduced the core from 357 to 355 and the current acquisition-unestablished group from 239 to 237; neither change established an acquisition method. FT-0133’s age-boundary note was corrected to at least 65 years without changing eligibility. FT-0637 remained restricted to its original, entirely adult 37-person report population; its recovery-selected design did not justify extrapolation to the 125-person parent cohort. Full source locations and retained-use restrictions are given in Supplementary Table S9.

## 4. Discussion

### 4.1. Principal Findings and Their Clinical Meaning

Three observations emerge from the mapped records. First, verified automated measurement is more prominent in cardiac-arrest and mixed neurological/trauma reports than in trauma reports. Second, ocular and toxicological contexts predominantly address diagnostic or physiological questions, whereas cardiac-arrest reports predominantly address neurological prognosis. Third, the numerical-age rule changes the clinical composition: adding age-unverified records increases the trauma share by 7.2 percentage points. The first two contextual patterns persist in that sensitivity scenario, but the apparent timing of automated measurement does not. These findings support matching a pupil result to its clinical question and acquisition process rather than treating all pupil-related evidence as interchangeable.

A useful distinction is between four levels of inference: whether a measurement can be reproduced, whether it identifies a target condition, whether it predicts a subsequent outcome, and whether using it improves patient management. Evidence at one level does not automatically establish the next. Agreement between a manual examination and a device, for example, is not equivalent to diagnostic accuracy against an independent reference standard; prognostic association does not demonstrate that acting on a result benefits patients. Diagnostic, prediction-model, and observational reporting frameworks help make these differences explicit [18,19,20].

The endpoint distinction is consequential even in apparently similar device studies. Comparing a nurse’s diameter estimate with a device evaluates measurement agreement; testing a sign against a clinical reference evaluates diagnosis; relating that sign to future functional status evaluates prognosis. A descriptive management change after a measurement does not establish benefit from pupil-guided care. Co-primary coding is reserved for reports whose pupil-related objectives genuinely span these categories.

Examples from emergency care further illustrate the distinction between measurement and clinical utility. Pupillometry has been investigated in relation to emergency-department disposition [21]. Point-of-care ultrasound has also been used to assess a relative afferent pupillary defect in adult ocular trauma against an ophthalmologist’s diagnosis [22]. These are different clinical questions, and operator-guided ultrasound is not automated infrared pupillometry. Neither an exploratory disposition association nor diagnostic performance alone establishes the benefit of a pupil-guided management strategy.

### 4.2. Automated Measurements Require Context-Specific Interpretation

Automated pupillometry can standardize aspects of stimulus delivery and quantify changes that are difficult to describe with a categorical bedside label. Historical measurement comparisons and later cardiac-arrest cohorts illustrate this potential [23,24,25]. These citations provide clinical context and are not all core-map inclusions. The present map cannot establish overall superiority of automated over manual assessment because patient selection, device, pupil parameter, timing, endpoint, and reference standard differ. A meaningful comparison requires a specified clinical question and acquisition protocol.

Device-derived parameters also require precise naming. The Neurological Pupil Index was introduced as a composite measure in relation to intracranial pressure; it is not simply another name for pupil diameter or a single constriction parameter. Combining differently defined measurements under a generic abnormal-pupil label can conceal important differences in what was measured. Background references illustrating this terminology are not automatically additional eligible reports in the core map [26].

The sample showed recoverable verification gaps: 12 of 50 initially unestablished classifications were resolved as manual. Among 38 remaining unresolved sampled reports, 29 lacked an explicit acquisition description in the materials examined, eight had ambiguous evidence, and one required unavailable supporting material. These results concern examined materials, not proven omissions from every article and supplement. The 199 unsampled retained reports may contain additional recoverable classifications. Thus, 237/355 is a mixed-verification ledger total, not a uniform assessment of source reporting. The conditional 53.3% estimate and 45.8–58.7% scenario range show the effect of assuming similar verification yield, without changing any unsampled record. Differential source accessibility and residual classification error could invalidate that assumption.

Clinical transferability also requires caution. A pupil finding during extracorporeal support, a serial neurological examination after trauma, and an ocular diagnostic evaluation have different possible mechanisms. The ORANGE and Miroz cohorts illustrate distinct populations, and their presence in one report-level map does not justify pooling them [7,8]. Post-cardiac-arrest guidelines emphasize timing, avoidance of confounding, and multimodal interpretation [9,10]. The AHA research-standards statement additionally identifies bias introduced when prognostic findings influence withdrawal of life-sustaining treatment [27]. This map does not override those safeguards.

### 4.3. A Practical Reporting Agenda for Future Studies

The reporting considerations in Supplementary Table S8 are author-proposed minimum information for future source verification, not an empirically validated checklist or a count of omissions in the original articles. Priorities include the assessed eye and timing, stimulus and acquisition procedure, device and metric, eligible denominator, target condition or endpoint, and how the measurement influenced care. These fields would allow a subsequent reader to determine which inferential question the reported result can answer.

Denominators require equal attention. The number of enrolled patients may differ from the number with a usable pupil assessment, the number of examined eyes, and the number of repeated observations. A complete age-defined subgroup is also different from a subset selected after survival or follow-up. Studies combining pupil findings with other predictors should distinguish the development sample, validation sample, missing-data handling, and performance attributable to the full model from any pupil-only association. The role of outcome adjudication, masking, and clinical decisions informed by the index finding should be made transparent. These priorities align with STARD, TRIPOD + AI, and STROBE reporting guidance and the QUIPS framework for assessing bias in prognostic-factor research [18,19,20,28].

Future quantitative syntheses should link reports from shared cohorts before combining results, because repeated analyses do not provide independent patient samples. Severe-TBI management guidelines provide clinical context, not validation of every pupil measure [29]. The IMPACT and GCS-P publications illustrate the use of pupil reactivity within multivariable prognostic frameworks and draw on overlapping IMPACT/CRASH data resources [30,31]. These contextual models do not supply missing adult-subgroup data for reports excluded from this archive.

### 4.4. Relation to Focused Pupillometry Syntheses

Focused reviews address questions that this map intentionally does not answer. Martínez-Palacios and colleagues examined quantitative pupillometry for intracranial-pressure monitoring in traumatic brain injury, while Kiani and colleagues reviewed its prognostic role in traumatic brain injury [32,33]. The present contribution is the cross-context distinction among measurement agreement, clinical diagnosis/physiology, prognosis, and management, together with a reproducible examination of how documentary eligibility changes a fixed archive. It does not replace those focused syntheses or extend their conclusions to unverified methods.

### 4.5. Strengths and Limitations

The analysis provides a reproducible, source-linked account of a fixed collection. Its strengths are explicit eligibility restrictions, separation of acquisition evidence from inferred technique, a one-report/one-primary-category rule, and sensitivity analysis of the largest exclusion category. Search counts, candidate relationships, and historical versus current review fields remain distinguishable instead of being combined into a falsely complete selection pathway.

The main limitation is selection. The original route into the archive is incompletely reconstructable, the supplemental searches were unevenly processed, and neither the 355 external candidate union nor the remaining historical retrieval failures has been integrated. The retrospective query test also found both additional candidates and a known methodological article missed by every tested variant. Accordingly, this is a preselected subset of unknown representativeness; zero cells and period patterns cannot establish field-wide gaps or trends. The three investigator-directed retrieval closures add possible selection against older evidence.

The numerical-documentation requirement may exclude genuinely adult cohorts. Its effect was differential: 135 of 251 trauma reports in the combined scenario were age-unverified, compared with 17 of 56 cardiac-arrest reports. All 50 sampled EX07 decisions remained unverifiable on review; this supports application of the documentary rule within that sample, not the claim that those cohorts included children. The sensitivity map shows the resulting compositional change without asserting adult eligibility. Its EX07 acquisition classifications were not comprehensively re-audited and therefore remain subject to the same verification limitations.

Acquisition and setting uncertainty, restricted adult subgroups, unresolved numerical denominators, and possible cohort overlap limit further synthesis. Acquisition verification was not uniform: 38 unestablished labels were retained after sampled reconciliation, while 199 were not acquisition-audited. The scenario range reflects uncertainty in the sampled resolution proportion under an exchangeability assumption; it does not capture all extraction error, establish source-reporting completeness, or resolve the unexamined records. Reports were not transformed into an independent patient count. A canonical endpoint simplifies multifaceted studies; secondary findings remain accessible. Without map-wide risk-of-bias assessment, neither a cell size nor a method frequency demonstrates superiority.

Historical dual-human review with three-person consensus, the investigator-adopted 325-record verification batch, and Jen-Tang Sun’s subsequent source verification are distinguished. The random sample targeted two uncertainty strata rather than all reports or domains. The 43/50 age result represents Sun’s confirmed first-pass judgments before reconciliation, as clarified by the authors; restoration of seven inclusions does not convert it to 100% initial agreement. Masking during that first pass could not be established. The 239-record eligibility exercise cannot be treated as a 239-record acquisition audit or a fresh full reading of every PDF. Tool-assisted preparation and comparisons are described in Section 2.4; human judgments, original returns and later reconciliations remain separate (Supplementary Table S9).

## 5. Conclusions

In this preselected acute-care archive, pupil-related clinical questions and documented acquisition differ by context. Diagnostic/physiological questions predominate in ocular and toxicological reports, while neurological prognosis predominates after cardiac arrest. The adult-documentation rule materially changes the trauma share, and acquisition uncertainty remains substantial under sensitivity analysis. The map supplies an auditable within-archive comparison, not a census of the field or evidence of comparative clinical effectiveness.

## Figures and Tables

**Table 1:** Clinical context and acquisition method.

Domain/Category	Reports, *n*	%
**Etiology**		
Traumatic brain injury/trauma	116	32.7
Cerebrovascular disease	74	20.8
Cardiac arrest	39	11.0
Ocular/neuro-ophthalmic	32	9.0
Toxicological/pharmacological	29	8.2
Mixed neurological/trauma	27	7.6
Other acute/mixed	13	3.7
Infection	9	2.5
Iatrogenic/perioperative	5	1.4
Other neurological	4	1.1
Metabolic/systemic	3	0.8
Mixed acute neurological	2	0.6
Physiological/benign mimic	1	0.3
Unclear	1	0.3
**Assessment method**		
Not established total	237	66.8
Sampled and reconciled	38	10.7
Not sampled for acquisition	199	56.1
Automated	53	14.9
Manual	42	11.8
Manual + automated	16	4.5
Other mixed methods	4	1.1
Other instrumented	3	0.8
**Care setting**		
Hospital; unit not established	120	33.8
ICU	93	26.2
ED	66	18.6
Multiple settings	25	7.0
Ophthalmic care	20	5.6
Other hospital	13	3.7
Prehospital or community	10	2.8
Not established	5	1.4
Research or volunteer	2	0.6
Neurosurgical care	1	0.3

Core map, *n* = 355. Each report contributes once per domain. The two subrows beneath Not established total partition that category by acquisition-audit coverage and are not additional categories. Sampled refers to the 50-report acquisition stratum, not the later eligibility audit. Automated does not establish the absence of manual assessment; mixed categories identify documented additional methods.

**Table 2:** Etiology by documented acquisition method in the 355 core reports.

Etiology	Auto	Manual	Both	Mixed	Other Inst.	NE Sampled	NE Unsampled	Total	Any Auto
Traumatic brain injury/trauma	5	4	1	0	0	20	86	116	6
Cerebrovascular disease	9	8	0	0	0	9	48	74	9
Cardiac arrest	10	7	7	0	0	2	13	39	17
Ocular/neuro-ophthalmic	1	8	1	3	1	2	16	32	3
Toxicological/pharmacological	1	6	0	0	0	3	19	29	1
Mixed neurological/trauma	9	4	6	0	0	1	7	27	15
Other acute/mixed	7	2	1	1	0	0	2	13	9
Infection	4	1	0	0	0	1	3	9	4
Iatrogenic/perioperative	2	2	0	0	0	0	1	5	2
Other neurological	2	0	0	0	0	0	2	4	2
Metabolic/systemic	1	0	0	0	0	0	2	3	1
Mixed acute neurological	2	0	0	0	0	0	0	2	2
Physiological/benign mimic	0	0	0	0	1	0	0	1	0
Unclear	0	0	0	0	1	0	0	1	0
Total	53	42	16	4	3	38	199	355	71

Core *n* = 355. Six canonical acquisition categories remain mutually exclusive; Not established is displayed as two disjoint verification-status columns. NE sampled: still unresolved after the sampled acquisition audit and source reconciliation (*n* = 38). NE unsampled: not selected for that acquisition audit (*n* = 199), although eligibility was later checked. Any auto is an overlapping component flag, not another category. Both, manual and automated; Mixed, other mixed methods; Other inst., other instrumented. All acquisition columns except Any auto sum to the row total. Instrumented measurement with manual grading is not automatically automated pupillometry.

**Table 3:** Etiology by primary pupil-related endpoint.

Etiology	D/AP	Neuro	Death	Multiple	Mgmt	Other	Methods	Total
Traumatic brain injury/trauma	22	28	40	19	5	2	0	116
Cerebrovascular disease	23	16	16	12	5	1	1	74
Cardiac arrest	4	27	6	1	0	1	0	39
Ocular/neuro-ophthalmic	21	0	0	1	0	10	0	32
Toxicological/pharmacological	23	1	1	1	1	2	0	29
Mixed neurological/trauma	8	8	0	6	3	0	2	27
Other acute/mixed	6	2	2	0	1	0	2	13
Infection	5	1	2	1	0	0	0	9
Iatrogenic/perioperative	2	3	0	0	0	0	0	5
Other neurological	1	1	0	1	1	0	0	4
Metabolic/systemic	3	0	0	0	0	0	0	3
Mixed acute neurological	2	0	0	0	0	0	0	2
Physiological/benign mimic	1	0	0	0	0	0	0	1
Unclear	1	0	0	0	0	0	0	1
Total	122	87	67	42	16	16	5	355

Core map, *n* = 355. Each report contributes once within each domain. D/AP, diagnosis or acute physiology; Neuro, neurological outcome; Death, mortality; Multiple, co-primary or multiple; Mgmt, management or disposition; Other, other clinical outcome; Methods, methodological outcome. Zero cells describe this archive only.

## Data Availability

Supplementary Tables S1–S9, the Separate S4 Report-Decision Index, Reporting Checklists, and Supplementary Data File S1 are provided for publication alongside this article. Supplementary_Data_File_S1.zip contains the coded report-level analytic dataset (JSON/CSV), searchable decision ledger (HTML), compact audit and sampling records, data dictionary, executable reproduction code, and reference outputs. Original review forms, detailed source-verification notes, and retained historical search, deduplication, and version records not included in the public file are available on reasonable request from Jen-Tang Sun (femh83316@femh.org.tw), subject to applicable access and copyright restrictions. Copyrighted source-article PDFs and unpublished companion manuscripts are not included in the public file; their redistribution is not promised.

## Supplementary Materials

The following supporting information can be downloaded at https://www.mdpi.com/article/10.3390/diagnostics16213698/s1: The materials provided for publication alongside this article are the scope file and Tables S1–S9, the separate S4 report-decision index, the reporting checklists, and Supplementary Data File S1 (Supplementary_Data_File_S1.zip). The data file contains the coded analytic dataset, searchable report-level decision ledger, compact audit and sampling records, data dictionary, reproduction code, and reference outputs. Detailed historical records not included in this public file are available as specified in the Data Availability Statement. Reports are not assumed to represent independent cohorts.

## Abbreviations

The following abbreviations are used in this manuscript:

AI	Artificial intelligence
ED	Emergency department
ICU	Intensive care unit
IQR	Interquartile range
NPi	Neurological Pupil Index
PRISMA	Preferred Reporting Items for Systematic Reviews and Meta-Analyses
PROSPERO	International Prospective Register of Systematic Reviews
TBI	Traumatic brain injury
