# Supplementary material for: An Evidence Map of Pupillary Assessment in a Preselected Acute-Care Literature Archive
Source: Diagnostics (Basel). 2026 Nov 9;16(21):3698. doi: 10.3390/diagnostics16213698 (PMC13761962; doi:10.3390/diagnostics16213698)
Supplement: Supplementary file 1 [file diagnostics-16-03698-s001.zip › 03_Supplementary_Tables_S1-S9.pdf]

SCOPE file

An Evidence Map of Pupillary Assessment in a Preselected Acute-Care Literature Archive

This file defines the analysis scope and supplies Supplementary Tables S1-S9 for the report-level evidence map. The analysis uses 355 core reports from an 871-record historical frame; 15 qualitative/linkage-only reports are separate. It is not a completed field-wide literature update or a diagnostic-accuracy meta-analysis.

Presentation revision 6 November 2026; underlying eligibility dispositions remain dated 30 October 2026. Current dispositions are 355 core, 15 qualitative/linkage-only, 497 criteria exclusions, three unresolved-source closures and one duplicate. Of the 237 core records still labelled acquisition not established, 38 received the sampled acquisition check and source reconciliation, while 199 did not receive that acquisition audit. The subsequent 239-record exercise assessed eligibility, not acquisition. S7 and S9 separate these verification strata and present a conditional sensitivity scenario. S4 is supplied as a separate decision index; its searchable ledger and the analysis materials are designated for publication as described below. There are nine numbered supplementary tables.

Item	Contents
S1	Scope, eligibility hierarchy and disposition totals
S2	Search provenance, original deduplication and verbatim logged PubMed expressions
S3	Eligibility rules and mapping definitions
S4	Report-level decisions and exclusions; separate full index and ledger
S5	Source-specific updates and unresolved main reports
S6	Fifteen qualitative-only reports
S7	All six core-map distributions and exact cross-tabulations
S8	Restricted uses and author-proposed reporting recommendations
S9	Review provenance, appraisal limitations and reporting status

Publication materials: this S1-S9 PDF, the separate S4 report-decision index, the reporting checklists, and Supplementary Data File 1 (Supplementary_Data_File_1.zip) are provided for publication alongside the article. The ZIP contains the coded report-level analytic dataset, bibliographic identifiers and decision codes, six-domain classifications and component flags, the searchable HTML decision ledger, compact audit and sampling data, the data dictionary, expected counts and reproduction code. These materials allow readers to trace the published report counts and rerun the analysis without requesting the underlying historical files. Original review forms, detailed source-verification records, and retained historical search, deduplication and version logs not included in the public supplement are available on reasonable request from Jen-Tang Sun (femh83316@femh.org.tw). Historical filenames cited below identify retained records; only files listed in the Supplementary Data File 1 README belong to the public data ZIP. Copyrighted source-article PDFs and the unpublished companion manuscript are not included; access to additional sources is subject to copyright and permissions. No separate public repository or repository DOI is claimed.

Supplementary Table S1 Scope and current report disposition

The analysis unit is a report, not a unique cohort or a summed patient population. PROSPERO CRD420261441918 was registered on 5 July 2026. The supplied export, printed on 19 July 2026, lists only Version 1.0 published on 5 July 2026. The additionally supplied PROSPERO PDF is byte-identical to that archive and does not independently document a later amendment. The original framework explicitly includes evidence mapping and adults aged at least 18 years; the subsequent numeric-documentary rule, terminal non-inclusion rule and exact category hierarchy are distinguished in S9 rather than attributed retrospectively to the original registration.

Disposition	Reports	Interpretation
Core map	355	All current core percentages use n = 355.
Qualitative only	15	14 descriptive series plus one report-linkage companion; outside core percentages.
Criteria exclusions	497	Includes 274 reversible EX07 adult-evidence exclusions.
Unresolved correct complete source	3	FT-0609, FT-0643 and FT-0897. Retrieval stopped by investigator; not verified eligibility failures.
Duplicate alias	1	Removed from report counting; historical record retained.
Total historical frame	871	355 + 15 + 497 + 3 + 1 = 871.

The operational master uses Exclude for all 500 non-included nonduplicate records. Its three author-directed retrieval closures are displayed separately in Figure 1 and this supplement. A zero active-request count means no outstanding retrieval request in the workflow, not complete physical retrieval of the historical frame.

The investigator-supplied archive date is 7 July 2026. Separate establishment and final-lock events, and the contemporaneous rationale for closing the archive, were not independently documented. The author-confirmed last search date is 17 July 2026; the preserved primary PubMed run is independently dated 5 July 2026. The present source verification and manuscript synchronization are not a new field-wide search.

Supplementary Table S2 Search provenance and original deduplication

PubMed via NCBI Entrez E-utilities esearch and efetch; strategy v1.1; executed 5 July 2026. No date, language, adult, human, or publication-type limits were applied in the executed log. The module labels Q1-Q5 below correspond exactly to the source log labels S1-S5; Q notation distinguishes query modules from the numbered supplementary tables.

Module	Purpose	Appearances
Q1	Acute/emergency abnormal pupil phenotype	2369
Q2	Neurocritical prognostic pupillometry	588
Q3	Toxicological and pharmacological causes	2050
Q4	Acute ophthalmic and neuro-ophthalmic emergencies	3594
Q5	Prehospital, police and first response	352
Sum	Module appearances, with overlap	8953
Union	Unique PMIDs	7440
Overlap removed	Repeated appearances across modules	1513

Repeated appearances are not necessarily duplicate publications. Original identifier and duplicate-review records are retained byte-for-byte in Original_Search_and_Review_Archive.zip and Recovered_Search_Review_Provenance.zip. The historical cross-source integration recorded 142 merge events; this is distinct from the 1,513 PubMed module repeats and the one alias removed from the current 871-record frame. Possible-duplicate flags were not silently converted into confirmed deletions.

The original download list had 1,236 rows. The reconciled retrieval ledger had 1,239 IDs: 871 historically mapped, 166 historical exclusions and 202 historical retrieval failures. The ledger incorporates 31 added UF/manual IDs and omits 28 base-list FT IDs; it is not the original list plus three reports. These historical categories are not the current Figure 1 dispositions. Their original selection pathway cannot be fully reconstructed.

Recovered supplementary-source inventory

Source label in preserved files	Appearances	Groups	No usable title
CENTRAL	113	113	0
DOAJ	4	4	0
Epistemonikos	587	478	210
Europe PMC	1314	986	0
GoogleScholar-labelled list / OpenAlex proxy provenance	81	81	0
NICE MIB	1	1	0
OpenAIRE	323	322	165
OpenAlex	1595	1593	670
medRxiv	2	2	0
medRxiv/PubMed	1	1	0

Across these snapshots, 4,021 appearances were normalized into 3,065 groups, of which 931 lacked a usable title. Source rows may overlap, so source-specific groups must not be summed as unique reports. Structured identifiers linked 651 groups to PubMed; 2,414 had no confirmed PubMed link. An unmatched group is not proof of a new eligible report. Full planned-source processing was not completed; the tracker retains incomplete work, including LILACS/BVS and BASE. A GoogleScholar-labelled list has OpenAlex-proxy provenance and is not presented as an independently executed Google Scholar search.

An older PubMed master retained 1,430 out-of-archive Include/Maybe candidates. Later queue decisions were 335 Include, 78 Unclear and 1,017 Exclude. A distinct later technical-audit set retained 325 Include plus 10 Unclear, and excluded 1,095. These two 335-record retained sets are not identical: 315 are shared and 20 are unique to each, giving a union of 355 candidates. All 355 lie outside the current 871-report frame. Historical inclusion labels are candidate-stage decisions, not current full-text eligibility or integration. The exact crosswalk below reconciles the stages without adding them to the core denominator.

Verbatim logged query expressions

Block A - abnormal pupil phenotype (reused by S1, S3, S4, and within S5)

"Pupil Disorders"[Mesh] OR "Anisocoria"[Mesh] OR "Miosis"[Mesh] OR "Mydriasis"[Mesh] OR anisocoria*[tiab] OR "unequal pupil*"[tiab] OR "pupil asymmetr*"[tiab] OR "pupillary asymmetr*"[tiab] OR miosis[tiab] OR miotic[tiab] OR "pinpoint pupil*"[tiab] OR "constricted pupil*"[tiab] OR mydriasis[tiab] OR mydriatic[tiab] OR "dilated pupil*"[tiab] OR "blown pupil*"[tiab] OR "fixed pupil*"[tiab] OR "fixed dilated pupil*"[tiab] OR "nonreactive pupil*"[tiab] OR "non-reactive pupil*"[tiab] OR "unreactive pupil*"[tiab] OR "sluggish pupil*"[tiab] OR "abnormal pupil*"[tiab] OR "pupillary abnormal*"[tiab] OR "relative afferent pupillary defect"[tiab] OR (RAPD[tiab] AND (pupil*[tiab] OR pupillary[tiab] OR afferent[tiab])) OR "dilation lag"[tiab] OR "light-near dissociation"[tiab] OR dyscoria[tiab] OR corectopia[tiab] OR "irregular pupil*"[tiab] OR "distorted pupil*"[tiab]

S1 = A AND S1C - S1C (acute/emergency context):

"Emergency Medical Services"[Mesh] OR "Emergency Service, Hospital"[Mesh] OR "Critical Care"[Mesh] OR "Intensive Care Units"[Mesh] OR prehospital[tiab] OR "out-of-hospital"[tiab] OR ambulance*[tiab] OR paramedic*[tiab] OR "first responder*"[tiab] OR "emergency department*"[tiab] OR "critical care"[tiab] OR "intensive care"[tiab] OR neurocritical[tiab] OR coma[tiab] OR unconscious*[tiab] OR "altered mental status"[tiab] OR "altered consciousness"[tiab] OR collapse[tiab] OR "brain death"[tiab] OR "traumatic brain injur*"[tiab] OR "head injur*"[tiab] OR "intracranial hemorrhage*"[tiab] OR "intracranial haemorrhage*"[tiab] OR "intracerebral hemorrhage*"[tiab] OR "intracerebral haemorrhage*"[tiab] OR "subarachnoid hemorrhage*"[tiab] OR "subarachnoid haemorrhage*"[tiab] OR stroke[tiab] OR "brain herniation"[tiab] OR "cerebral herniation"[tiab] OR "cardiac arrest"[tiab] OR hypoxi*[tiab] OR anoxi*[tiab] OR seizure*[tiab] OR "status epilepticus"[tiab] OR meningitis[tiab] OR encephalitis[tiab] OR hypoglyc*[tiab] OR "respiratory failure"[tiab]

S2 = (pupillometry/reactivity) AND (acute brain / critical illness) AND (outcome / prognosis)

Block 1: "Reflex, Pupillary"[Mesh] OR pupillometr*[tiab] OR "quantitative pupillometry"[tiab] OR "neurological pupil index"[tiab] OR "neurologic pupil index"[tiab] OR (NPi[tiab] AND (pupil*[tiab] OR pupillary[tiab])) OR "pupillary reactiv*"[tiab] OR "pupil reactiv*"[tiab] Block 2: "Brain Injuries"[Mesh] OR "Brain Injuries, Traumatic"[Mesh] OR "Intracranial Hemorrhages"[Mesh] OR "Stroke"[Mesh] OR "Brain Herniation"[Mesh] OR "Cardiac Arrest"[Mesh] OR "Coma"[Mesh] OR "Critical Illness"[Mesh] OR "traumatic brain injur*"[tiab] OR "acute brain injur*"[tiab] OR "subarachnoid hemorrhage*"[tiab] OR "subarachnoid haemorrhage*"[tiab] OR "intracranial hemorrhage*"[tiab] OR "intracranial haemorrhage*"[tiab] OR stroke[tiab] OR "cardiac arrest"[tiab] OR coma[tiab] OR neurocritical[tiab] OR "critical* ill"[tiab] OR "intensive care"[tiab] OR "status epilepticus"[tiab] OR postanoxic[tiab] OR "hypoxic-ischemic"[tiab] OR "hypoxic ischaemic"[tiab] Block 3: "Mortality"[Mesh] OR "Prognosis"[Mesh] OR "Treatment Outcome"[Mesh] OR mortality[tiab] OR death[tiab] OR fatal*[tiab] OR prognos*[tiab] OR outcome*[tiab] OR deterioration[tiab] OR herniation[tiab] OR intubat*[tiab] OR ventilat*[tiab] OR "neurologic* outcome*"[tiab] OR "neurological outcome*"[tiab]

S3 = A AND S3C - S3C (toxicological/pharmacological):

"Poisoning"[Mesh] OR "Drug Overdose"[Mesh] OR "Substance-Related Disorders"[Mesh] OR "Alcoholic Intoxication"[Mesh] OR poisoning[tiab] OR overdose[tiab] OR intoxication[tiab] OR toxidrome*[tiab] OR opioid*[tiab] OR fentanyl[tiab] OR clonidine[tiab] OR organophosph*[tiab] OR anticholinergic*[tiab] OR sympathomimetic*[tiab] OR cocaine[tiab] OR amphetamine*[tiab] OR alcohol*[tiab] OR methanol[tiab] OR "carbon monoxide"[tiab] OR scopolamine[tiab] OR ipratropium[tiab] OR atropine[tiab] OR brimonidine[tiab] OR Datura[tiab] OR "topical medication*"[tiab] OR "medication exposure"[tiab] OR iatrogenic[tiab]

S4 = A AND S4C - S4C (acute ophthalmic/neuro-ophthalmic):

"Eye Injuries"[Mesh] OR "Oculomotor Nerve Diseases"[Mesh] OR "Horner Syndrome"[Mesh] OR "Glaucoma, Angle-Closure"[Mesh] OR "Carotid Artery Dissection"[Mesh] OR "Aneurysm, Intracranial"[Mesh] OR "ocular trauma"[tiab] OR "eye injur*"[tiab] OR "acute angle closure"[tiab] OR "angle-closure glaucoma"[tiab] OR "third nerve pals*"[tiab] OR "oculomotor pals*"[tiab] OR "Horner syndrome"[tiab] OR "Horner's syndrome"[tiab] OR "carotid dissection"[tiab] OR "intracranial aneurysm*"[tiab] OR "compressive aneurysm*"[tiab] OR "optic neuritis"[tiab] OR "orbital compartment syndrome"[tiab]

S5 = S5P AND S5C - S5P (phenotype OR quantitative assessment) = Block A OR pupillometr*[tiab] OR "quantitative pupillometry"[tiab] OR "neurological pupil index"[tiab] OR "neurologic pupil index"[tiab] OR (NPi[tiab] AND (pupil*[tiab] OR pupillary[tiab])) OR "pupillary reactiv*"[tiab] OR "pupil reactiv*"[tiab]; S5C (prehospital/first-response):

"Emergency Medical Services"[Mesh] OR prehospital[tiab] OR "out-of-hospital"[tiab] OR ambulance*[tiab] OR paramedic*[tiab] OR "emergency medical technician*"[tiab] OR EMT[tiab] OR EMS[tiab] OR "first responder*"[tiab] OR police[tiab] OR "law enforcement"[tiab] OR custody[tiab] OR detention[tiab]

The expressions above are reproduced without silently repairing the logged search. The source log reports unrecognized MeSH strings for Brain Herniation, Cardiac Arrest, Carotid Artery Dissection and Aneurysm Intracranial, and an outstanding sentinel-article check. The original search-strategy document and raw query log are retained. Source-specific historical execution details that cannot be reconstructed are not presented as completed or as searched on 17 July merely because that was the author-confirmed overall last date.

S2a. Chronology and different denominators

Date	Event	Interpretation
5 July 2026	Original registry submission/registration and Version 1.0 publication. Preserved PubMed module execution log.	Registry pp. 7, 14; original PubMed log. These are not the archive lock date.
7 July 2026	Investigator-reported historical archive date.	Separate establishment/final-lock events and contemporaneous closing rationale are not independently documented.
17 July 2026	Author-confirmed overall last search date; contemporary download stamps also occur on some reports.	Does not establish that every database was run on that date; source-specific logs control execution claims.
16 September 2026	Numeric documentary age threshold confirmed.	Later operational restriction, not verbatim prospective registration.
11-12 October 2026	Terminal non-inclusion rule and source/eligibility corrections.	Closed operative report dispositions; no field-wide update.
28 October 2026	Technical search-syntax audit, crosswalk and descriptive sensitivity calculations.	Current database with publication cutoff 17 July 2026; not reconstruction of July indexing.

8,953 is the sum of PubMed appearances across five query modules; 7,440 is their unique-PMID union. The 871-report frame is a separately reconciled historical sample, not the number left after a documented eligibility screen of all 7,440 records. The current 355 core reports are selected from the 871 frame using the rules and dispositions in S1 and S3. The original path from source identification to historical archive selection remains incompletely reconstructed.

S2b. Out-of-frame retained-candidate crosswalk

Candidate stage	Records	Historical exclusion	Historical not retrieved	No historical link
Queue Include set	335	88	148	99
Later-audit retained set	335	97	156	82
Union of the two retained sets	355	97	156	102

For the 355-record union, 97 + 156 + 102 = 355. The 253 linked candidates have historical dispositions; these do not demonstrate that all are newly eligible. The remaining 102 are not established to be 102 unique eligible reports. No external candidate has been integrated into the present core map. The exact ID-level set membership and links are retained in candidate_crosswalk_rows.json. Final cross-source deduplication, missing-metadata recovery, retrieval and eligibility integration remain incomplete.

S2c. Retrospective technical audit of logged syntax

A retrospective technical audit of the preserved PubMed syntax was performed on 28 October 2026, applying a publication-date cutoff of 17 July 2026 to every comparison. The original modules retrieved 7,493 unique PMIDs in the current database. Substitution of three canonical MeSH headings increased retrieval to 7,534; separately replacing the unsupported Brain Herniation heading with brain/cerebral herniation title/abstract terms increased retrieval to 7,536. The 43 additional PMIDs are retained as an unintegrated candidate set, not new eligible reports. Four of five post hoc named examples were retrieved; the measurement-agreement example by Meeker et al. remained undetected because it did not satisfy the relevant phenotype or outcome/context requirements. These findings identify technical and structural limitations of the archived search and do not establish field-wide completeness. Exact requests, raw responses, translations, warnings, and UID lists are retained with the research records.

**Variant, executed 28 October 2026**	**Unique PMIDs**	**Difference on the same date**
Original logged syntax, current run	7493	Reference current-date run
Three canonical MeSH corrections	7534	41 additional PMIDs
Separate herniation free-text change	7536	2 further PMIDs; 43 total additions
**Original literal heading**	**Tested replacement**	**Type of change**
Cardiac Arrest[Mesh]	Heart Arrest[Mesh]	Canonical descriptor correction
Carotid Artery Dissection[Mesh]	Carotid Artery, Internal, Dissection[Mesh]	Canonical descriptor correction
Aneurysm, Intracranial[Mesh]	Intracranial Aneurysm[Mesh]	Canonical descriptor correction
Brain Herniation[Mesh]	brain/cerebral herniation title/abstract terms	Separate free-text test, not an asserted exact MeSH synonym

All comparisons used the same publication-date cutoff of 17 July 2026. The archived values 8,953 appearances and 7,440 unique PMIDs remain unchanged. Subtracting 7,440 from 7,536 would confound database/indexing changes with syntax corrections and is not the incremental yield of corrected headings. None of the 43 added candidates has undergone eligibility integration in this technical audit.

**Post hoc example**	**PMID**	**Archived union**	**Current original**	**Current expanded**
Miroz 2020	31870911	Yes	Yes	Yes
ORANGE 2023	37652068	Yes	Yes	Yes
Oddo 2018	30478620	Yes	Yes	Yes
Meeker 2005	15794443	No	No	No
IMPACT 2008	18684008	Yes	Yes	Yes

These five examples are post hoc checks, not a recovered prespecified sentinel set or a search-sensitivity estimate. Meeker 2005 satisfies the current Q2 pupil and clinical-context blocks but not its required outcome block; it also fails the shared abnormal-pupil phenotype block and Q5 prehospital context. Heading correction therefore does not remedy every

structural omission. Exact query specifications, responses, warnings, translations and UID sets are retained in Research_Records/Search_Syntax_Audit_20261028.

Supplementary Table S3 Eligibility and charting definitions

Rule	Operational definition
Adult evidence	Numerical age evidence establishes at least 18 years. The word adult, means, medians or an IQR alone do not establish the required lower bound.
Mixed age	Only a complete identifiable adult subset defined independently of a subsequent outcome can support restricted inclusion. The reviewer must not construct an age-known rescue subset by selecting later survivors, treatment responders, ICU disposition or a later complication within an otherwise age-unverified report population. An entire originally defined report population with independently documented adult ages is not made nonadult by an outcome-selected study design; its selection bias still limits use.
Clinical context	Acute illness, injury, exposure, unexplained pupil findings, or prespecified clinically relevant diagnostic-mimic contexts.
Qualitative exception	14 descriptive series plus one BOX companion abstract (FT-1174), retained solely for report linkage, not as an independent study. All 15 are outside core counts and percentages.
Source identity	Own article title, authors, year and identifiers plus article-header/front-page evidence. Identifiers found only in a reference list cannot authenticate the PDF.
Uncertainty	Preserve unresolved age or acquisition evidence; do not infer children, manual assessment or ICU location from absence of detail.
**Mapping domain**	**Definition**
Aetiology	Clinical context supported by the mapped pupil evidence; not prevalence of causes among all patients.
Assessment method	Manual, automated, both, other instrumented, other mixed methods, or not established. Categorical reactivity alone does not establish manual acquisition.
Care setting	Where the relevant pupil assessment occurred, not the department in the affiliation. ICU admission does not establish the location of an initial measurement.
Primary pupil-related endpoint	Endpoint supported by the pupil evidence: diagnosis/acute physiology, neurological outcome, mortality, management/disposition, other clinical, methodological, or multiple. Clinical diagnosis/physiology is distinct from measurement agreement, reliability, or misclassification against another method. Use the primary pupil-related aim; preserve secondary outcomes.
Study design	Canonical design plus retained source detail. Diagnostic/prognostic purpose is not itself a study-design category.
Publication period	Final journal issue year grouped as before 2000, 2000-2009, 2010-2019 or 2020-2026. Unequal periods are not annualized.

Total enrolled people, pupil-assessed people, eyes, model nodes and repeated readings remain separate fields. Aetiology and endpoint cross-tabulations allocate one category per core report. Method-component flags are nonexclusive; 53 classified as Automated + 16 manual-and-automated + 2 other mixed-method reports = 71 reports with any automated component. Automated does not establish the absence of manual or other assessment.

Report sufficiency and BOX companion handling

Non-core material comprised 14 descriptive series and one sufficiently informative preliminary BOX-trial abstract (FT-1174), retained solely to document its link with the full report (FT-0693). The companion abstract was not treated as an independent study or included in core percentages. Conference abstracts were assessed for extractable eligibility and outcome information rather than excluded by publication label: a second BOX abstract (FT-1147) lacked the required standalone population and outcome details and was excluded.

Report	Own-source information	Current use
FT-1174; DOI 10.1093/ehjacc/zuad036.141	PDF p. 2 / printed i208: 710 patients, age 18-93; CPC 3-5 at 3 months; serial quantitative measures and threshold estimates.	Linkage only to FT-0693, not an independent study and not in the core 355.
FT-1147; DOI 10.1093/ehjacc/zuad036.142	PDF pp. 1-2 / printed i210-i211: arm-specific 48-hour AUCs, but no age criterion, participant denominators or explicit target-outcome definition/horizon.	Excluded for insufficient standalone information, not the abstract label alone. Family link preserved.

Primary pupil-related endpoint: operational hierarchy

Decision	Operational rule
Unit	Pupil-related principal research objective, established from Methods and Results rather than title keywords or every outcome appearing in the paper.
Methodological	Agreement, repeatability, inter-rater reliability, instrument interchangeability or misclassification of the same measured pupil feature against a measurement comparator. Words accuracy, sensitivity or ROC alone do not establish clinical diagnostic validity.
Diagnosis acute physiology	Clinical disease/lesion/mimic identification or localisation, independently adjudicated clinical deficit, or association with a contemporaneous clinical physiological state. Diagnostic and physiological subtypes should be visible in secondary notes because these do not all represent diagnostic-accuracy studies.
Coprimary	Use the co-primary/multiple endpoint category only when distinct pupil-related clinical domains are genuinely co-primary. Do not count the report separately in each primary category.
Secondary	Retain measured secondary clinical physiology and management observations as separate flags; do not infer clinical benefit from measurement agreement or a descriptive management change.
Unclear	If source aims and analyses cannot establish a primary endpoint, retain an unclear category rather than guessing from the title.

Worked source examples

Report	Assigned primary endpoint	Source basis and distinction
FT-0223; Robba 2020	Other methodological outcome	Primary goal compares size, quantitative PLR, velocity and latency between two devices using agreement statistics. Discussion explicitly says the study did not assess outcome, other neuromonitoring or pupil-guided management. Descriptive ICU mortality among 112 participants is not a pupil-linked mortality endpoint. Manual and two-device comparisons establish acquisition methods but do not demonstrate clinical diagnostic accuracy. Source: PDF p. 2 / printed p. 324 and PDF p. 6 / printed p. 328.
FT-0296; Kerr 2016	Other methodological outcome	Purpose is nurses' pupil-diameter/symmetry accuracy and thresholds where subjective assessment degrades. Clinical phase 3 compares bedside assessment with NPi-100 in eligible patients >=18; drawings/photo phases are distinct. Anisocoria/reactivity misclassification versus a measuring instrument is not lesion-diagnostic accuracy or proof of patient-outcome improvement. Restrict mapped clinical data to phase 3. Source: PDF pp. 2-3 / printed pp. 214-215.
FT-0299; Couret 2016	Other methodological outcome	Primary aim is standard-versus-automated assessment of pupil size, anisocoria and PLR. Main text reports 12 changes in management but explicitly states the trial was neither designed nor powered to assess patient-outcome impact. Keep methodological primary classification. Preserve secondary sufentanil-dose/pupil-size association and reported management changes without converting them to co-primary clinical outcomes or demonstrated treatment benefit. Exact management actions require Additional file 5/Table S1 and were not reverified here. Source: PDF p. 2 objectives/methods and PDF p. 8 limitations and conclusion; supplementary extraction ledger pp. 3-5 for secondary physiological association.
FT-0520; Gotoh 2001	Other methodological outcome	Purpose is weighted stroke-scale construction; 378 clinical participants have ages 42-88. The separate 1274-person variable-selection cohort has only mean/SD age. Pupil-item reliability and weighting concern scale development. Methodological/scale-development design, not cross-sectional clinical diagnostic performance. Do not import historical cohort prognostic effects into adult-eligible pupil-specific evidence. Source: PDF p. 2 / printed p. 1801; current source ledger cites Table 6 on p. 4 / printed p. 1803 and Table 7/Figure 2 on p. 5 / printed p. 1804.
FT-0764; Yic 2020	Other methodological outcome	Explicit objective is correlation between ultrasonographic and infrared assessment in critically ill patients; ultrasound measurement is manual and paired with infrared within 3 minutes. Interchangeability of pupil measurements, not accuracy for herniation or prediction of functional outcome. Repeated measurements are not independent people. Source: PDF p. 2 objective/methods; current source ledger pp. 3-6 for sample and results.
FT-0080; Ramamoorthy 2024	Diagnosis/acute physiology	Primary outcome explicitly diagnostic accuracy of POCUS-guided RAPD and EP clinical examination against a blinded ophthalmologist's final RAPD diagnosis, assessed within 1 hour in adult ocular trauma. Clinical deficit adjudication against an independent specialist reference, unlike diameter interchangeability. This supports diagnostic classification, but does not establish improved treatment outcomes. Source: PDF p. 3 / printed p. 1345, Outcome Measures and preceding reference-standard description.

Adult-subset boundary and outcome-selected report designs

The prohibition concerns review-created adult rescue subsets, not a blanket exclusion of outcome-selected study designs. FT-0120 cannot be rescued by seven later ICU admissions, and UF-43BA3EE5 cannot be rescued by adult naloxone nonresponders. By contrast, the original FT-0637 Part I report explicitly defines its complete analytic population as 37 recovering post-arrest patients aged 31-82 (PDF p. 1 / printed p. 133, title and Summary; PDF p. 2 / printed p. 134, Patients). That report may be retained only as descriptive recovery-phase pupil evidence in those 37 adults. Their ages cannot establish adulthood of the 125-person parent cohort, and the selected series cannot estimate prognosis in an unselected arrest population. A complete clinical group defined by a new acute index event before its pupil observations is distinct from a subgroup selected by a later pupil-related outcome.

Supplementary Table S4 Report level decisions and exclusion reasons

The separate S4 PDF lists all 871 report IDs with author/year, title, persistent identifiers and current disposition/reason code. Supplementary Data File 1 contains the searchable HTML report-decision ledger, coded analytic data and mapping fields for tracing the published counts. Detailed source-verification records and original review forms remain available on reasonable request from Jen-Tang Sun (femh83316@femh.org.tw), rather than being represented as part of the public ledger. Mapping completion is separate from numerical-reuse restrictions: FT-0671 and FT-0691 remain core inclusions, while their source/version ambiguities prevent unqualified numerical reuse. No new pooled analysis is performed.

Current criterion category	n	Preserved raw code
Numerical adult eligibility unverified	274	EX07
Paediatric or mixed age without separable eligible adult pupil data	196	EX02_POPULATION 176; EX02 20
No eligible pupil observations or pupil-related results	16	EX03 10; four long-code variants 6
No separable eligible acute/clinical context	8	EX04
Single-case technical demonstration	1	EX01
Secondary review rather than original evidence	1	EX01_PUBLICATION_TYPE
Conference report with insufficient data	1	EX_CONFERENCE_INSUFFICIENT_DATA
Total criteria exclusions	497	Not including three author-directed retrieval closures

The raw reason codes come from different historical coding versions. The display labels above were checked against the actual saved reasons; the original codes are preserved and not treated as self-explanatory universal categories. EX07 exclusions remain reversible and do not indicate confirmed children.

Supplementary Table S5 New source checks and unresolved reports

Report	Source and decision	Supporting location
FT-0046 Shen 2025	Complete 5-page main article; n = 245, age 15-92. Exclude EX02; no complete baseline-defined adult subset verified. DOI 10.1097/SCS.0000000000011124; PMID 39913905.	p. 1698 age; p. 1699/Figure 2 pupil associations. Publisher supplementary Tables 1-3 not retrieved.
FT-0047 Rana 2025	Complete 5-page main article; n = 77. Adult label and means/SDs do not establish numerical minimum. Exclude EX07, reversible. DOI 10.1097/JNN.0000000000000818; PMID 39883016.	pp. 83-87; cohort 27 versus 50. NCT02804438 has no minimum age and permits multiple age groups; this does not prove minors in these 77. External appendix not retrieved.
FT-0090 Chilakapati 2024	Complete 6-page main article; whole cohort n = 2568, matched sample n = 1002. Numerical minimum not established. Exclude EX07, reversible. DOI 10.1097/TA.0000000000004255; PMID 38196125.	pp. 938-943; OSF project zfu7n and linked registration cqy4a do not establish numeric adult eligibility. External matching figure not retrieved.

All three are removed from active missing-main-text requests. This does not imply access to all external supplements. The full new-source evidence and SHA256 identity records are retained. If a verified complete adult cohort/subgroup or numerical eligibility evidence is later recovered, the relevant decision may be reconsidered.

Unresolved ID	Remaining problem	Disposition
FT-0609 Sarabia 1988	Missing printed pages 42-46; PMID 3189019.	Author-directed non-inclusion; retrieval stopped.
FT-0643 Price 1979	Missing printed pages 181-182; PMID 158292.	Author-directed non-inclusion; retrieval stopped.
FT-0897 Feldman 1991	Correct target article unresolved; prior PDF was a different article.	Author-directed non-inclusion; retrieval stopped.

Earlier source-based corrections also remain applied: FT-0879 EX07 because the 22-78-year range covers only ten outcome-selected survivors, not the whole 111-arrest population; FT-1073 EX04 because the separately identifiable adult-eye data cannot be linked to an eligible acute subgroup; FT-1042 IN02 only for descriptive evidence from the complete 18-person model node aged at least 38.5 years, not whole-cohort pupil performance or independent adult validation.

Outside the current 871-record frame, the retained dated overlay corrects FT-1233 to the actual healthy adult-volunteer/clinical-scope rationale rather than childhood. It also preserves the verified UF download-copy relationship. These historical-ledger corrections do not add reports to the current 355-report core denominator.

FT-0964 Bawaskar 1982 is excluded as EX02. The ten pulmonary-oedema cases arose after baseline assessment among 51 mixed-age scorpion-sting patients; the two 23-year-old adults in Tables III-IV are an outcome-selected subgroup, not a complete baseline adult cohort. This is not an EX07 absence-of-numeric-age exclusion. Source: PDF pp. 1-3, Patients, Tables I-IV, Results and Discussion; PMID 6120403.

30 October 2026 eligibility audit: changed decisions and boundary clarifications

Report	Current disposition	Source-based conclusion	Locator
FT-0120	Exclude; EX07	The 122-patient ketamine-intoxication cohort has only a mean age of 26.7 years (SD 6.5), without a numerical adult entry criterion or observed full age range. The seven individually aged ICU patients were selected by disposition after emergency care, explicitly listed as an outcome. This subsequent-outcome subgroup cannot establish adult eligibility for the report under the current rule. Adult eligibility is unverified; this is not evidence of confirmed paediatric enrollment.	PDF p.2 / printed p.7, Methods: disposition after emergency care; PDF p.3 / printed p.8, Table 1: ICU admission under Desenlace; PDF p.7 / printed p.12, Table 2: seven ICU cases
UF-43BA3EE5	Exclude; EX02	The report evaluates 43 naloxone recipients. The 28 treatment responders include ages 14-83, while the 15 nonresponders are 33-89. Group assignment was determined by improvement after naloxone. Selecting only adult nonresponders cannot establish an adult baseline cohort, and adult pupil findings from the mixed-age responders cannot be separated. The source documents mixed ages, unlike an age-unverified exclusion.	PDF p.2 / printed p.447, Methods and Results: response-defined allocation; PDF p.3 / printed p.448, Table 4: group age ranges; PDF p.4 / printed p.449: chart documentation limitations
FT-0133	Include; IN02	Retain only the age-defined elderly stratum and its admission-BFDP subgroup for separately reported adult pupil-linked outcomes. Group membership uses admission age/pupils, not subsequent survival. The full 497-person mixed-age model cannot enter adult synthesis.	PDF pp2-4 Methods and Results/Table1; PDF pp4-7; Figure3 p6 visually checked; PDF pp.2-4, Methods/Results and Table 1; PDF p.6 Figure 3 (image checked) and p.7 Discussion
FT-0637	Include; IN01	The complete analytic population of this separately published Part I report consists of 37 patients aged 31-82 who regained consciousness, explicitly defined in its title, abstract and Patients section. No age-known subset is newly constructed by this review. Retain the report only as descriptive recovery-phase pupil evidence in those 37 adults. Do not treat the 125-person parent cohort as adult-verified or use the selected series to estimate prognosis in an unselected arrest population.	PDF p.1 / printed p.133, title and Summary; PDF p.2 / printed p.134, Introduction and Patients

Only FT-0120 and UF-43BA3EE5 change eligibility. FT-0133 remains included: enlarged original Figure 3 shows age >=65 in both elderly labels, matching Methods/Table 1; the earlier >65-versus->=65 discrepancy was a transcription error and has been removed from current notes. Other numerical inconsistencies and adult-subset restrictions remain. FT-0637 remains descriptive-only within its original 37-person adult report population. These are source-based technical reconciliations, not a claim of another independent human review.

Supplementary Table S6 Qualitative only reports

The 15 records below comprise 14 descriptive series and the BOX companion abstract FT-1174, retained solely for report linkage, not as an independent study. None contributes to core counts, percentages or pooled estimates. The source notes below preserve earlier qualifications; any pending-confirmation wording belongs to those historical notes and is not a new pending eligibility state. The operative disposition is qualitative only, with all source restrictions retained; no unresolved numerical effect is declared ready for pooling.

FT-0466 A mixture of organophosphate and pyrethroid intoxication requiring intensive care unit admission: a diagnostic dilemma and therapeutic approach.

PMID: 16861425; year: 2006. Eight clinically relevant adult mixed-insecticide intoxications with actual pupil findings and treatment changes. The source is labeled Case Report but contains an eight-patient series; completeness or consecutive ascertainment is not stated, so the rare-condition series exception requires second review.

Recorded age evidence: Table 1 provides all eight ages: 48, 34, 58, 42, 38, 44, 41 and 62 years. Current use: qualitative description only, outside all core percentages and pooled estimates. Earlier quantitative restriction: Not cleared by this mapping-normalization revision; endpoint-specific extraction and study-family checks remain separate.

FT-0498 Pseudohypoxic brain swelling: a newly defined complication after uneventful brain surgery, probably related to suction drainage.

PMID: 14633298; year: 2003. Seventeen adult postoperative cases include six with documented pupil abnormalities. Cases were largely recalled from memory and the authors explicitly do not claim completeness; suitable only for a rare-syndrome qualitative series pending protocol judgment.

Recorded age evidence: Individual ages in Table 1 range 21-67 years; all 17 adults. Six pupil cases are aged 46, 38, 44, 21, 45 and 32. Current use: qualitative description only, outside all core percentages and pooled estimates. Earlier quantitative restriction: Not cleared by this mapping-normalization revision; endpoint-specific extraction and study-family checks remain separate.

FT-0510 Collective poisoning with hallucinogenous herbal tea.

PMID: 12208022; year: 2002. A multi-patient adult acute poisoning outbreak with documented mydriasis and laboratory-confirmed exposure is clinically relevant, but clinical sampling is not demonstrably consecutive and pupil counts are absent. Retain only as qualitative rare-exposure evidence pending author scope confirmation.

Recorded age evidence: More than 30 event participants explicitly aged 20-50. Current use: qualitative description only, outside all core percentages and pooled estimates. Earlier quantitative restriction: Not cleared by this mapping-normalization revision; endpoint-specific extraction and study-family checks remain separate.

FT-0523 Acute amitraz intoxication in human.

PMID: 11430553; year: 2001. Retrospective 10-case rare poisoning report contains a complete separately extractable five-adult subgroup, but consecutive recruitment and timeframe not stated. Retain adult phenotype/clinical trajectory qualitatively pending author case-series scope judgment.

Recorded age evidence: Adult Table 1 ages 26,30,20,34,19; separate pediatric Table 2 ages 4-14. Current use: qualitative description only, outside all core percentages and pooled estimates. Earlier quantitative restriction: Not cleared by this mapping-normalization revision; endpoint-specific extraction and study-family checks remain separate.

FT-0587 Tegmental pontine hemorrhages: clinical features and prognostic factors.

PMID: 1623452; year: 1992. Six adult spontaneous tegmental pontine hemorrhages with actual miosis and clinically relevant lesion characterization. Consecutive ascertainment is not specified, so eligibility relies on the larger rare-condition series exception and needs confirmation.

Recorded age evidence: Results p 1 observed ages 41-78, average 59.8. Current use: qualitative description only, outside all core percentages and pooled estimates. Earlier quantitative restriction: Not cleared by this mapping-normalization revision; endpoint-specific extraction and study-family checks remain separate.

FT-0600 Fatal central diabetes mellitus and insipidus resulting from untreated hyponatremia: a new syndrome.

PMID: 2294815; year: 1990. Eleven fully adult acute cases provide clinically relevant pupil-herniation diagnostic observations, but the series was retrospectively referred by families, physicians or attorneys and selected for a fatal rare syndrome. No population prognosis rate or independent pupil effect can be estimated.

Recorded age evidence: PDF p 1 explicitly adults over 18; p 2 actual ages 23-47. Current use: qualitative description only, outside all core percentages and pooled estimates. Earlier quantitative restriction: Not cleared by this mapping-normalization revision; endpoint-specific extraction and study-family checks remain separate.

FT-0642 Unilateral inferior temporal lobectomy with hippocampectomy for relief of incisural herniation.

PMID: 474209; year: 1979. Large historical non-consecutive clinical series with complete individual tables allowing 23 adults to be separated; preserve descriptive surgical experience, not comparative efficacy or unselected prognosis.

Recorded age evidence: Tables 1-3 list all 30 cases:23 adults aged 18-60;7 below 18. Adult counts 12 in survivors table,9 in deaths table,2 in no-herniation table. Current use: qualitative description only, outside all core percentages and pooled estimates. Earlier quantitative restriction: Not cleared by this mapping-normalization revision; endpoint-specific extraction and study-family checks remain separate.

FT-0947 Cluster of wound botulism in California: clinical, electrophysiologic, and pathologic study.

PMID: 9324085; year: 1997. Six adult hospital cases with structured clinical, electrophysiological and biopsy investigations are informative for rare acute wound botulism, but consecutive/complete ascertainment is not explicit; retain descriptive findings pending protocol judgment.

Recorded age evidence: All six individually tabulated ages 44,41,42,31,56,44 years. Current use: qualitative description only, outside all core percentages and pooled estimates. Earlier quantitative restriction: Not cleared by this mapping-normalization revision; endpoint-specific extraction and study-family checks remain separate.

FT-1021 Traumatic orbital compartment syndrome: importance of the lateral canthomy and cantholysis.

PMID: 24810151; year: 2014. Eight adult rare traumatic OCS cases with clinical pupil findings; consecutivity/completeness of ascertainment not explicitly established, so retain descriptive synthesis pending scope review.

Recorded age evidence: Ages 22-86, all eight ages individually listed in Table 1. Current use: qualitative description only, outside all core percentages and pooled estimates. Earlier quantitative restriction: Not cleared by this mapping-normalization revision; endpoint-specific extraction and study-family checks remain separate.

FT-1030 Acute optic neuritis following infection with chikungunya virus in southern rural India.

PMID: 21131222; year: 2011. Ten adult cases of acute post-chikungunya optic neuritis have complete individual age/pupil data and longitudinal findings. A 6-week epidemic period is specified, but consecutive or complete ascertainment of all eligible cases is not stated. Retain this larger rare-condition series for qualitative synthesis pending protocol judgment, not an unselected prognostic cohort.

Recorded age evidence: Results p 2 and individual Table 1 ages establish 22-45 years for all 10 patients. Current use: qualitative description only, outside all core percentages and pooled estimates. Earlier quantitative restriction: Not cleared by this mapping-normalization revision; endpoint-specific extraction and study-family checks remain separate.

FT-1033 Surgical treatment of severely traumatized eyes with no light perception.

PMID: 19952999; year: 2010. Complete age-defined adult subgroup of 13/18 operated cases is recoverable from Table 1. Prospective interventional case series with consecutive sampling not stated; retain restricted adult qualitative evidence pending selection and numerical reconciliation.

Recorded age evidence: Individual Table 1 ages identify adults 21-65 in rows 1,2,3,5,6,9,10,12,13,15,16,17,18; exclude rows 4,7,8,11,14 aged 8,11,3,8,17. Current use: qualitative description only, outside all core percentages and pooled estimates. Earlier quantitative restriction: Not cleared by this mapping-normalization revision; endpoint-specific extraction and study-family checks remain separate.

FT-1045 Abducens nerve palsy and postganglionic Horner syndrome with or without severe headache.

PMID: 16908941; year: 2006. Nine adult cases of a rare clinically localizing syndrome with complete individual descriptions. Consecutive/all-case sampling is not stated over the 26-year interval; retain qualitative diagnostic evidence, not population rates.

Recorded age evidence: Observed ages 28-63; Table 1 all nine adults. Current use: qualitative description only, outside all core percentages and pooled estimates. Earlier quantitative restriction: Not cleared by this mapping-normalization revision; endpoint-specific extraction and study-family checks remain separate.

FT-1071 Monocular temporal hemianopia.

PMID: 8343470; year: 1993. Retain qualitatively only the complete adult at-presentation stratum in this larger rare-condition series; do not use the mixed-age 24-case aggregate or delayed postoperative cases for frequencies. This supports the differential diagnosis of unexplained monocular temporal field loss, including functional/disc-dysversion mimics.

Recorded age evidence: Table 1 individual ages: exclude the 17-year-old case 23; the 15 adult cases whose interval is 0 are 19-66. Current use: qualitative description only, outside all core percentages and pooled estimates. Earlier quantitative restriction: No pooled analysis performed or cleared by this 325-record adjudication. Adult subsets must not inherit mixed-age whole-cohort effects.

FT-1093 Meningiomas and aneurysms of the cavernous sinus. Neuro-ophthalmologic features.

PMID: 629685; year: 1978. The source reports 16 adult cases, with a complete identifiable acute-onset aneurysm subgroup of four (cases 9, 10, 11, 16). Retain this subgroup descriptively; most remaining cases were chronic, and consecutive recruitment is not established. No acute population incidence or prognosis can be inferred.

Recorded age evidence: Individual Table: all 16 patients aged 47-78; the four acute-onset aneurysm cases are 71, 78, 54 and 47 years. Current use: qualitative description only, outside all core percentages and pooled estimates. Earlier quantitative restriction: Not cleared by this mapping-normalization revision; endpoint-specific extraction and study-family checks remain separate.

FT-1174 Quantitative pupillometry thresholds for early neuroprognostication after cardiac arrest: A BOX-trial substudy

PMID: Not established; year: 2023. Adult 710-person BOX pupillometry substudy with substantive results, but preliminary companion report of the later full publication. Retain for report linkage; do not use as an independent study or duplicate the full-report estimates.

Recorded age evidence: Actual range 18-93, mean 63±14, in 710 measured patients. Current use: report linkage only; not an independent study and outside all core counts, percentages and pooled estimates. Earlier quantitative restriction: Not cleared by this mapping-normalization revision; endpoint-specific extraction and study-family checks remain separate.

Supplementary Table S7 Core map distributions and reproducibility

Aetiology

Category	Reports	Percent of 355
Traumatic brain injury/trauma	116	32.7
Cerebrovascular disease	74	20.8
Cardiac arrest	39	11.0
Ocular/neuro-ophthalmic	32	9.0
Toxicological/pharmacological	29	8.2
Mixed neurological/trauma	27	7.6
Other acute/mixed	13	3.7
Infection	9	2.5
Iatrogenic/perioperative	5	1.4
Other neurological	4	1.1
Metabolic/systemic	3	0.8
Mixed acute neurological	2	0.6
Physiological/benign mimic	1	0.3
Unclear	1	0.3
Total	355	100.0

Assessment method

Category	Reports	Percent of 355
Not established - sampled and reconciled	38	10.7
Not established - not acquisition-audited	199	56.1
Automated	53	14.9
Manual	42	11.8
Manual and automated	16	4.5
Mixed other methods	4	1.1
Other instrumented	3	0.8
Total	355	100.0

The two not-established rows partition one canonical acquisition category (237/355, 66.8%); they are not separate acquisition techniques. Not acquisition-audited means outside the additional 50-record acquisition sample, not never screened or never checked for eligibility.

Care setting

Category	Reports	Percent of 355
Hospital; unit not established	120	33.8
ICU	93	26.2
ED	66	18.6
Multiple settings	25	7.0
Ophthalmic care	20	5.6
Other hospital	13	3.7
Prehospital or community	10	2.8
Not established	5	1.4
Research or volunteer	2	0.6
Neurosurgical care	1	0.3
Total	355	100.0

Primary pupil-related endpoint

Category	Reports	Percent of 355
Diagnosis/acute physiology	122	34.4
Neurological outcome	87	24.5
Mortality	67	18.9
Co-primary/multiple	42	11.8
Management/disposition	16	4.5
Other clinical outcome	16	4.5
Other methodological outcome	5	1.4
Total	355	100.0

Study design

Category	Reports	Percent of 355
Retrospective cohort / record analysis	162	45.6
Prospective observational study	64	18.0
Secondary analysis	42	11.8
Case series	32	9.0
Cross-sectional	20	5.6
Cohort, timing unclear	14	3.9
Randomized trial	9	2.5
Measurement / methodological study	6	1.7
Ambispective / mixed-time cohort	2	0.6
Case-control	2	0.6
Other interventional / experimental	2	0.6
Total	355	100.0

Publication period

Category	Reports	Percent of 355
2020-2026	165	46.5
2010-2019	109	30.7
2000-2009	48	13.5
Before 2000	33	9.3
Total	355	100.0

Supplementary Data File 1 (Supplementary_Data_File_1.zip) is provided for publication with the article. It contains the coded report-level analytic inputs, searchable S4 decision ledger, compact audit and sampling data, data dictionary, expected counts and Reproduce_All_Counts.py. The expected/Descriptive_Counts.json file preserves the underlying six-domain counts; expected/Round2_Verification_Summary.json includes verification-status splits, the combined-frame matrix and the conditional acquisition scenario. Additional original forms, detailed source records and historical search/deduplication/version logs can be requested from Jen-Tang Sun (femh83316@femh.org.tw). Rounding can prevent displayed percentages from summing exactly to 100. The report-level frame does not support a total of unique patients or independent studies.

FT-0223, FT-0296, FT-0299 and FT-0520 were recoded to methodological outcome, alongside FT-0764. FT-0299 retains secondary acute-physiology and management observations. Source evidence is in the report ledger and Correction_Source_Audit.json.

The supplementary direct-comparison field contains 12 Yes, 10 No and 333 Unclear among 355 current core reports. Yes requires source-supported manual-versus-automated comparison within eligible adult data, not merely use of both methods. Different-time performance comparisons are not synchronous agreement studies. Whole mixed-age results

cannot be transferred to a restricted adult subset. No requires affirmative evidence; otherwise retain Unclear. Methods Section 2.5 and Direct_Comparison_Audit_355.json provide current definitions, comparison types, timing, population scope and source locations. The former 357-record audit is retained only as a historical snapshot.

S7a. Descriptive eligibility-rule sensitivity analysis: 355 core + 274 EX07 records

This deliberately relaxed analysis adds all 274 age-unverifiable EX07 records to the 355 current core reports (n = 629). It includes newly excluded FT-0120 only in this relaxed sensitivity frame, using its retained mapping categories; the confirmed mixed-age exclusion UF-43BA3EE5 is not relaxed. The original 273 EX07 mapping records were not reclassified from ancillary acquisition answers in the 100-record audit. This is not an alternative eligible adult cohort or a causal estimate of age-rule bias. No source-availability, clinical-context or confirmed mixed-age exclusion is relaxed. All primary results retain n = 355.

Aetiology - core versus relaxed age-documentation frame

Category	Core 355, n (%)	EX07 274, n (%)	Combined 629, n (%)	Change, pp
Cardiac arrest	39 (11.0)	17 (6.2)	56 (8.9)	-2.1
Cerebrovascular disease	74 (20.8)	49 (17.9)	123 (19.6)	-1.3
Iatrogenic/perioperative	5 (1.4)	1 (0.4)	6 (1.0)	-0.5
Infection	9 (2.5)	0 (0.0)	9 (1.4)	-1.1
Metabolic/systemic	3 (0.8)	1 (0.4)	4 (0.6)	-0.2
Mixed acute neurological	2 (0.6)	3 (1.1)	5 (0.8)	+0.2
Mixed neurological/trauma	27 (7.6)	16 (5.8)	43 (6.8)	-0.8
Ocular/neuro-ophthalmic	32 (9.0)	19 (6.9)	51 (8.1)	-0.9
Other acute/mixed	13 (3.7)	10 (3.6)	23 (3.7)	-0.0
Other neurological	4 (1.1)	0 (0.0)	4 (0.6)	-0.5
Physiological/benign mimic	1 (0.3)	0 (0.0)	1 (0.2)	-0.1
Toxicological/pharmacological	29 (8.2)	23 (8.4)	52 (8.3)	+0.1
Traumatic brain injury/trauma	116 (32.7)	135 (49.3)	251 (39.9)	+7.2
Unclear	1 (0.3)	0 (0.0)	1 (0.2)	-0.1

Care setting - core versus relaxed age-documentation frame

Category	Core 355, n (%)	EX07 274, n (%)	Combined 629, n (%)	Change, pp
ED	66 (18.6)	55 (20.1)	121 (19.2)	+0.6
Hospital; unit not established	120 (33.8)	78 (28.5)	198 (31.5)	-2.3
ICU	93 (26.2)	84 (30.7)	177 (28.1)	+1.9
Multiple settings	25 (7.0)	23 (8.4)	48 (7.6)	+0.6
Neurosurgical care	1 (0.3)	3 (1.1)	4 (0.6)	+0.4
Not established	5 (1.4)	6 (2.2)	11 (1.7)	+0.3
Ophthalmic care	20 (5.6)	6 (2.2)	26 (4.1)	-1.5
Other hospital	13 (3.7)	5 (1.8)	18 (2.9)	-0.8
Prehospital or community	10 (2.8)	12 (4.4)	22 (3.5)	+0.7
Research or volunteer	2 (0.6)	2 (0.7)	4 (0.6)	+0.1

Assessment method - core versus relaxed age-documentation frame

Category	Core 355, n (%)	EX07 274, n (%)	Combined 629, n (%)	Change, pp
Automated	53 (14.9)	34 (12.4)	87 (13.8)	-1.1
Manual	42 (11.8)	34 (12.4)	76 (12.1)	+0.3
Manual and automated	16 (4.5)	8 (2.9)	24 (3.8)	-0.7
Mixed other methods	4 (1.1)	1 (0.4)	5 (0.8)	-0.3
Not established	237 (66.8)	197 (71.9)	434 (69.0)	+2.2
Other instrumented	3 (0.8)	0 (0.0)	3 (0.5)	-0.4

Primary pupil-related endpoint - core versus relaxed age-documentation frame

Category	Core 355, n (%)	EX07 274, n (%)	Combined 629, n (%)	Change, pp
Co-primary/multiple	42 (11.8)	32 (11.7)	74 (11.8)	-0.1
Diagnosis/acute physiology	122 (34.4)	94 (34.3)	216 (34.3)	-0.0
Management/disposition	16 (4.5)	26 (9.5)	42 (6.7)	+2.2
Mortality	67 (18.9)	52 (19.0)	119 (18.9)	+0.0
Neurological outcome	87 (24.5)	60 (21.9)	147 (23.4)	-1.1
Other clinical outcome	16 (4.5)	9 (3.3)	25 (4.0)	-0.5
Other methodological outcome	5 (1.4)	1 (0.4)	6 (1.0)	-0.5

Study design - core versus relaxed age-documentation frame

Category	Core 355, n (%)	EX07 274, n (%)	Combined 629, n (%)	Change, pp
Ambispective / mixed-time cohort	2 (0.6)	2 (0.7)	4 (0.6)	+0.1
Case series	32 (9.0)	5 (1.8)	37 (5.9)	-3.1
Case-control	2 (0.6)	4 (1.5)	6 (1.0)	+0.4
Cohort, timing unclear	14 (3.9)	11 (4.0)	25 (4.0)	+0.0
Cross-sectional	20 (5.6)	13 (4.7)	33 (5.2)	-0.4
Measurement / methodological study	6 (1.7)	4 (1.5)	10 (1.6)	-0.1
Other interventional / experimental	2 (0.6)	3 (1.1)	5 (0.8)	+0.2
Other observational study	0 (0.0)	3 (1.1)	3 (0.5)	+0.5
Prospective observational study	64 (18.0)	47 (17.2)	111 (17.6)	-0.4
Randomized trial	9 (2.5)	4 (1.5)	13 (2.1)	-0.5
Retrospective cohort / record analysis	162 (45.6)	114 (41.6)	276 (43.9)	-1.8
Secondary analysis	42 (11.8)	64 (23.4)	106 (16.9)	+5.0

Publication period - core versus relaxed age-documentation frame

Category	Core 355, n (%)	EX07 274, n (%)	Combined 629, n (%)	Change, pp
2000-2009	48 (13.5)	41 (15.0)	89 (14.1)	+0.6
2010-2019	109 (30.7)	99 (36.1)	208 (33.1)	+2.4
2020-2026	165 (46.5)	105 (38.3)	270 (42.9)	-3.6
Before 2000	33 (9.3)	29 (10.6)	62 (9.9)	+0.6

For trauma, the relaxed frame contains 251 reports, including 135 age-unverifiable records (53.8% of that stratum); for cardiac arrest, 17 of 56 (30.4%) are age-unverifiable. These unequal documentation losses limit disease comparisons. The earliest core report with an automated component is from 2014; adding EX07 includes FT-1083 (Iris autonomic function in acute glaucoma, 1989). Neither date is the first use of automated pupillometry in the field. Unequal publication periods and selected archive coverage prohibit adoption-rate or historical-origin claims.

S7b. Aetiology by acquisition with verification status in the 355 core reports

Aetiology	Auto	Manual	Both	Mixed other	Other instr.	Not estab. audited	Not estab. not audited	Total	Any auto
Traumatic brain injury/trauma	5	4	1	0	0	20	86	116	6
Cerebrovascular disease	9	8	0	0	0	9	48	74	9
Cardiac arrest	10	7	7	0	0	2	13	39	17
Ocular/neuro-ophthalmic	1	8	1	3	1	2	16	32	3
Toxicological/pharmacological	1	6	0	0	0	3	19	29	1
Mixed neurological/trauma	9	4	6	0	0	1	7	27	15
Other acute/mixed	7	2	1	1	0	0	2	13	9
Infection	4	1	0	0	0	1	3	9	4
Iatrogenic/perioperative	2	2	0	0	0	0	1	5	2
Other neurological	2	0	0	0	0	0	2	4	2
Metabolic/systemic	1	0	0	0	0	0	2	3	1
Mixed acute neurological	2	0	0	0	0	0	0	2	2
Physiological/benign mimic	0	0	0	0	1	0	0	1	0
Unclear	0	0	0	0	1	0	0	1	0
Total	53	42	16	4	3	38	199	355	71

Auto = classified automated; Both = manual and automated; other instr. = other instrumented. The two not-established columns separate the 38 sampled and reconciled records from 199 records outside the acquisition audit. These sum to the single canonical not-established category (237). The first seven displayed method/status columns are mutually exclusive and sum to each row total. Any auto is nonexclusive, not an additional category. The 30 October eligibility audit did not establish acquisition methods for the unsampled records. Not established is a current ledger status, not confirmed absence of a technique or a conclusion that the original publication omitted it. The conditional scenario in S9f does not change any cell.

S7b2. Aetiology by acquisition in the combined core plus EX07 frame

Aetiology	Auto	Manual	Both	Mixed other	Other instr.	Not estab.	Total	Any auto
Traumatic brain injury/trauma	11	18	2	0	0	220	251	13
Cerebrovascular disease	19	14	3	0	0	87	123	22
Cardiac arrest	14	10	9	0	0	23	56	23
Toxicological/pharmacological	2	9	0	0	0	41	52	2
Ocular/neuro-ophthalmic	2	12	1	4	1	31	51	4
Mixed neurological/trauma	17	5	7	0	0	14	43	24
Other acute/mixed	9	5	2	1	0	6	23	12
Infection	4	1	0	0	0	4	9	4
Iatrogenic/perioperative	3	2	0	0	0	1	6	3
Mixed acute neurological	3	0	0	0	0	2	5	3
Metabolic/systemic	1	0	0	0	0	3	4	1
Other neurological	2	0	0	0	0	2	4	2
Physiological/benign mimic	0	0	0	0	1	0	1	0
Unclear	0	0	0	0	1	0	1	0
Total	87	76	24	5	3	434	629	113

Combined frame n = 629: 355 core reports plus 274 EX07 records. The six acquisition columns are mutually exclusive; Any auto is an overlapping component indicator. This matrix reproduces the combined-frame any-automated shares reported in Section 3.4: cardiac arrest 23/56 (41.1%), mixed neurological/trauma 24/43 (55.8%), and trauma 13/251 (5.2%). EX07 acquisition categories are retained historical classifications, not newly audited classifications. The combined not-established total is 434, comprising 237 core and 197 EX07 records. This is an eligibility-rule sensitivity frame, not an alternative set of confirmed adult inclusions.

S7c. Twelve source-supported direct manual-automated comparisons

Twelve Yes, ten No and 333 Unclear values remain among the 355 core reports. Yes is a documented comparison in eligible adult data, not coexistence of both methods. The table distinguishes paired measurement agreement from different-time prognostic-performance comparison. It is a minimum supported count, not the prevalence of all available comparisons. Ultrasound-only instrument comparison is not a conventional bedside manual-versus-automated comparison.

Report	Comparison type and timing	Eligible scope and source location
FT-0223; Robba, Chiara 2020	Paired measurement agreement and reactivity discrepancy. One paired ICU assessment per patient; manual examination followed by automated measurements about one minute later.	Retained adult clinical population. PDF pp. 2-3; printed pp. 324-325, Methods and Tables 1-2.
FT-0296; Kerr, Robert G 2016	Paired measurement agreement and reactivity discrepancy. Repeated bedside assessments, at least two hours apart; nurse assessment immediately followed by device measurement under the same lighting.	Retained adult clinical population. PDF pp. 3-5; printed pp. 215-217, phase-3 procedures and Tables 2-3.
FT-0299; Couret, David 2016	Blinded paired agreement and discordance. Entry within 48 hours of acute injury; paired daily measurements less than five minutes apart.	Retained adult clinical population. PDF pp. 2-3 and 5-7, Methods and comparison results.
FT-0333; Chen, Jefferson William 2014	Descriptive paired clinical discrepancy in an adult case series. Admission manual examination, hourly acute pupillometry and repeated clinical assessments. Follow-up measurements extend beyond the acute admission.	Complete four-person adult subgroup; illustrated discrepancies in adult cases 1 and 2. Do not transfer the paediatric case. PDF pp. 3-4, Table 1; pp. 7-9, adult cases 1 and 2.
FT-0707; Lenga, Pavlina 2024	Descriptive paired confirmation/discrepancy. NPi measured before clinical brain-death determination; a single examination event rather than long-term outcome follow-up.	Retained adult clinical population. PDF pp. 2-4, Methods and Tables 1-2.
FT-0751; Peluso, Lorenzo 2021	Same-cohort method-specific prognostic performance at different times. Manual examination at admission and at least twice daily; day-3 NPi compared with the worst manual reading over days 1-2.	Retained adult clinical population. PDF pp. 2-3 and 5-6, Methods and Table 2.

S7c. Direct comparisons (continued)

Report	Comparison type and timing	Eligible scope and source location
FT-0769; Olsen, Markus Harboe 2020	Paired size/reactivity agreement plus method-specific prognosis. Repeated ICU examinations, at least eight hours apart; temporal comparison differs from a single paired agreement test.	Retained adult clinical population. PDF p. 2, Methods; pp. 2-3 and 6, results/comparison figure.
FT-0788; Oddo, Mauro 2018	Same-time head-to-head prognostic performance. Daily examinations on days 1, 2 and 3 after arrest; the any-day summary is distinct from each daily comparison.	Retained adult clinical population. PDF pp. 2-3, Methods; p. 4, Figure 1 and Table 2; supplement Methods/Table 2.
FT-0793; Tamura, Tomoyoshi 2018	Same-cohort method-specific prognostic comparison. Scheduled at 0, 6, 12, 24, 48 and 72 hours after ROSC; initial manual examination timing differs from the later quantitative schedule.	Retained adult clinical population. PDF pp. 2-5, Methods, Results, Table 3 and Discussion.
FT-0815; Heimburger, Delphine 2016	Same-cohort outcome comparison at different times. Day-1 mean approximately 4 hours; day-2 examination after 24 hours of cooling; day-3 manual examination after rewarming and sedation cessation.	Retained adult clinical population. PDF pp. 2-3, Methods; Table 1 and Figure 1.
FT-0832; Suys, Tamarah 2014	Head-to-head prognostic accuracy at days 1 and 2. Day 1 approximately 16 hours during hypothermia; day 2 approximately 46 hours, after rewarming and sedation withdrawal.	Retained adult clinical population. PDF pp. 2-5, Methods, Table 1 and Tables 2-4.
FT-1005; Kim, Hyeong Min 2018	Retrospective descriptive discrepancy within inflammatory subgroup. Initial and follow-up examinations during recovery, with dark-adapted repeated device measurements; comparison limited to the inflammatory subgroup.	Manual comparator documented for the inflammatory subgroup, not the entire 171-person cohort. PDF pp. 2-4, Methods; pp. 7-8, Table 6; pp. 10-11, Discussion.

Supplementary Table S8 Restricted use and author proposed reporting considerations

This study performs descriptive mapping only. No report is cleared for pooling merely because it appears in the core map. Whole mixed-age-cohort effects cannot be transferred to an adult subset; matched, pupil-assessed and total-enrolment denominators are not interchangeable. Cohort linkage and question-specific risk-of-bias assessment would be required before any focused quantitative synthesis.

Author proposed minimum reporting considerations

Domain	Minimum information
Population and setting	Age threshold or range; recruitment; acuity; exclusions; clinical unit at the time of the pupil measurement.
Acquisition	Manual/device technique; model; stimulus; illumination; eye/laterality; units; measurement time; repeated readings; missingness.
Confounding	Sedatives and other relevant drugs; ocular disease/injury; procedures; physiological state at measurement.
Clinical question	Measurement agreement, diagnosis, prognosis or management effect; target condition or outcome; prespecified versus exploratory thresholds.
Outcome assessment	Definition, follow-up horizon, reference standard, masking, adjudication and influence of the pupil result on care or withdrawal decisions.
Denominators and models	Enrolled patients, assessed patients, eyes and observations; adult subgroup provenance; model development versus validation; pupil-only versus multivariable effect.
Reusability	Numerator/denominator or effect and uncertainty; exact time window; subgroup membership; shared-cohort identifiers.

These are author-proposed considerations informed by the mapping and established reporting frameworks cited in the main manuscript. They are not a formally developed, validated or externally endorsed guideline. Their inclusion is not a claim that all original articles omitted the listed information.

Map inclusion versus numerical reuse

Report	Map disposition	Unresolved numerical issue	Permitted interpretation
FT-0671	Core include; neurological primary endpoint remains supported.	Mean-versus-most-abnormal daily summary unspecified; source sedation event rates conflict; laterality/device and primary-versus-sensitivity cohort definitions unresolved. Do not reconstruct comparative event tables or pool effects.	Map eligibility and domain classification established from the available source. Endpoint-specific numerical reuse is restricted pending resolution of source inconsistencies; this is not an outstanding eligibility decision.
FT-0691	Core include; mortality primary endpoint remains supported.	Final publication abstract describes first five days, local manuscript entire ICU interval; final model denominator and discordant complication/death counts unresolved. Do not pool or release source-version-sensitive numerical estimates.	Map eligibility and broad acquisition/endpoint classification established.<br>Local-versus-final-version timing and numerical discrepancies restrict effect-estimate reuse; no pooled effect from this report is used in this map.

These holds do not make the existing map classifications pending. The original source-review notes remain in the historical layer. This revision does not claim that unexamined final-version tables or inconsistent source counts were resolved.

Report overlap with the focused cardiac-arrest companion manuscript

The current companion inventory contains 23 reports. Exact report/identifier linkage finds 14 of those reports among this map's 355 core reports, all classified under cardiac arrest. The table makes the overlap explicit; it does not equate 14 reports with 14 independent cohorts. Different inclusion rules, search processing and analysis questions explain why the inventories are not identical. The evidence map provides report distributions and does not reproduce pooled effect analyses from the focused manuscript.

Map ID	Companion citation	DOI	Companion family label
FT-0221	Riker et al., 2020	10.1007/s12028-019-00717-4	FAMILY-FT-0221
FT-0693	Nyholm et al., 2024	10.1007/s00134-024-07574-6	FAMILY-BOX
FT-0719	Nyholm et al., 2023	10.1016/j.resplu.2023.100399	FAMILY-COPENHAGEN-2015-2017
FT-0733	Peluso et al., 2022	10.1016/j.resuscitation.2022.07.030	FAMILY-ODDO-2018
FT-0737	Macchini et al., 2022	10.1016/j.resuscitation.2022.04.026	FAMILY-FT-0737
FT-0738	Paramanathan et al., 2022	10.1111/aas.14078	FAMILY-TTH48
FT-0748	Menozzi et al., 2022	10.1097/MAT.0000000000001569	FAMILY-ODDO-2018
FT-0751	Peluso et al., 2021	10.3390/brainsci11070888	FAMILY-FT-0751
FT-0768	Obinata et al., 2020	10.1016/j.resuscitation.2020.06.002	FAMILY-FT-0768-PROVISIONAL
FT-0772	Tamura et al., 2020	10.1371/journal.pone.0228224	FAMILY-TAMURA-2018
FT-0788	Oddo et al., 2018	10.1007/s00134-018-5448-6	FAMILY-ODDO-2018
FT-0793	Tamura et al., 2018	10.1016/j.resuscitation.2018.06.027	FAMILY-TAMURA-2018
FT-0815	Heimburger et al., 2016	10.1016/j.resuscitation.2016.02.026	FAMILY-FT-0815
FT-0832	Suys et al., 2014	10.1007/s12028-014-9981-z	FAMILY-FT-0832-PROVISIONAL

Supplementary Table S9 Review provenance and appraisal limitations

The authors clarified the functional roles on 4 November 2026: Chiao-Yin Cheng and Hua Ho independently performed the original screening and classification. Disagreements were resolved through joint discussion and consensus among Chiao-Yin Cheng, Hua Ho and Cheng-Yueh Li, rather than a separate independent third-reviewer adjudication. Jen-Tang Sun was separate from those original decision-makers and performed the additional source-validation review described below. This clarification supersedes the earlier shorthand description of independent third-human adjudication. It does not retrospectively assign a particular historical Reviewer 1 or Reviewer 2 export column to a named person where the export lacks that mapping. Historical screening and extraction records remain preserved.

Form preparation included Claude assistance with provisional entries. Jen-Tang Sun made the source-based judgments and personally validated the evidence for the 100 sampled records, the seven age queries and 16 acquisition proposals, and the later 239-record eligibility results. Codex assisted source comparisons, reconciliation records and technical consistency checks. The tools were not reviewers or final decision-makers. The authors retain responsibility for all classifications. Software also assisted language refinement, contextual comparisons, revision drafting and formatting; these supporting tasks are distinct from the human research judgments. Original and updated fields remain versioned separately.

No map-wide formal risk-of-bias appraisal was undertaken. Critical appraisal results, certainty-of-evidence ratings and pooled diagnostic or prognostic estimates are therefore not available. Funding of each included source was not systematically extracted and cannot be reported comprehensively; this differs from the funding declaration for the present work. Source-specific quantitative-use restrictions remain in the ledger.

Coverage limitations include the closed historical sampling frame, incomplete original selection lineage, incomplete out-of-archive 2026 integration, the stringent numeric-age rule, three report-specific retrieval closures, unestablished acquisition methods and unresolved shared-cohort overlap. Search linkage percentages are not search sensitivity or evidence completeness. Zero cells and unequal-period publication counts cannot establish field-wide gaps or adoption trends.

The completed reporting checklists explicitly identify not-applicable analyses and unavailable historical details. They document reporting coverage; they do not certify that every systematic-review procedure was performed.

S9a. Registration-to-implementation audit

The supplied original registration was visually checked on all 14 pages. The second PDF supplied during this revision has the same SHA256 (ac117941e9edc866697550f62cabf04537998bb3f4765e048e5482099595e779). Only Version 1.0 dated 5 July 2026 is documented by these files. Later procedural details below are disclosed here; this does not assert that they were already posted to the registry.

Analysis scope and unit

Element	Description
Registered framework	Broad systematic identification, narrative synthesis, evidence mapping and conditional meta-analysis; link related reports at study level (pp. 8, 11, 13).
Implemented analysis	Closed historical frame of 871 reports; 355 current core report-level entries and 15 qualitative/linkage entries, not a field-wide updated systematic census; no pooled effect estimate in this manuscript.
Date / evidence	Investigator-reported archive date: 7 July 2026. Eligibility disposition and document synchronization: 30 October 2026. The source archive does not independently document distinct assembly/final-lock events or the original closing rationale.
Status	Post-registration restriction of analytic frame and report-level descriptive application.
Reason and implication	Preserves a fixed auditable denominator despite incompletely reconstructed identification history and incomplete external-candidate integration. Findings describe this archive only; related reports are not independent cohorts.

Numeric documentary evidence of adulthood

Element	Description
Registered framework	Age >=18 and separately extractable adult data in mixed-age populations (p. 3).
Implemented analysis	Explicit minimum/actual range >=18 or a complete separately extractable adult subgroup is required. Adult labels, means, medians and IQRs alone do not establish numerical eligibility. Review-created outcome-selected subsets cannot rescue an otherwise age-unverified report; a complete original adult analytic population retains its study-design limitations (see S3 and S9e).
Date / evidence	Confirmed 16 September 2026 in Existing_Operational_Adjudication_Rules.json; terminal non-inclusion authorised 11 October 2026.
Status	Post-registration documentary restriction, not verbatim prospective registration.
Reason and implication	Creates auditable adult-only eligibility at a cost of selection by age documentation. EX07 denotes adult eligibility not verifiable numerically, not confirmed paediatric enrollment; reversible evidence-recovery list retained.

Terminal disposition of unresolved records

Element	Description
Registered framework	Contact authors where eligibility or source information unclear; record exclusions (pp. 8-9).
Implemented analysis	Previously unresolved 325-record batch adjudicated under explicit precedence rules. A decisive known violation is preferred to unknown age; age unverified after available-source checks becomes EX07; missing correct complete source remains a retrieval category. Unresolved metric questions are kept separate from map eligibility.
Date / evidence	11 October 2026 operational rule file and author instruction.
Status	Post-registration operational closure rule.
Reason and implication	Produces a closed disposition ledger while preserving reversibility and uncertainty rather than treating missing evidence as demonstrated ineligibility. Does not prove a new independent human read of every source.

Six-domain categories and primary-endpoint hierarchy

Element	Description
Registered framework	Mapping by phenotype, cause, assessment method, setting and outcome; broad design eligibility described (pp. 6, 11, 13).
Implemented analysis	One canonical aetiology, care setting, acquisition category, pupil-related primary endpoint, design and publication period per report; secondary endpoint flags retained separately. Methodological agreement separated from clinical diagnosis/physiology.
Date / evidence	Current taxonomy documented in the 12 October 2026 package; not evidence of a prospectively locked exact six-domain scheme.
Status	Operationalisation and later refinement of registered mapping intent.
Reason and implication	Prevents secondary clinical observations from being promoted to primary outcomes and prevents one report entering multiple primary-category counts. Publication-period bins and deterministic primary-category rules should be described as analytic choices.

Three investigator-directed retrieval closures

Element	Description
Registered framework	No publication-date restriction (p. 8).
Implemented analysis	FT-0609, FT-0643 and FT-0897 retained as unresolved-source closures, outside the 497 eligibility exclusions and 355 core reports.
Date / evidence	Author instruction and 12 October 2026 technical package; not a prospectively defined age-of-publication cutoff.
Status	Report-specific post hoc stop-retrieval decisions.
Reason and implication	May preferentially omit older evidence. Publication age was not an eligibility criterion; these were report-specific retrieval closures. Do not merge them with full-text eligibility exclusions.

BOX preliminary companion report

Element	Description
Registered framework	Link reports from the same cohort and retain useful associated data; exclude abstracts only when insufficiently extractable (pp. 6, 8).
Implemented analysis	FT-1174 retained only for linkage beside full BOX report FT-0693, not as an independent study or a core entry. It is the one companion abstract in 15 non-core records; the other 14 are descriptive series. FT-1147 excluded for missing essential standalone information.
Date / evidence	Current package rule clarified during this revision; exact evidence independently checked from local abstracts on 28 October 2026.
Status	Clarification of associated-report handling, not a new independent cohort.
Reason and implication	FT-1174 has explicit adult ages and extractable outcomes; FT-1147 does not. No transfer of missing abstract-level eligibility from the parent full report and no duplicate participants in analysis.

S9b. Returned judgments and subsequent source reconciliation

Chiao-Yin Cheng prepared and passed the completed form dated 29 October 2026 to Jen-Tang Sun for source-based judgment. The authors clarified on 4 November 2026 that the 43/50 adult-evidence confirmation result represents Sun's first-pass judgments after his confirmation and before the subsequent discrepancy reconciliation, not unvalidated provisional entries. Sun had not participated in the original screening or three-person consensus. The blank form omitted earlier categories and reasons, but the retained records do not establish whether he remained unaware of them during his first source review; no blinded human audit is claimed. The form date does not establish the exact dates of his confirmation or subsequent reconciliation. The sampling strata comprised 50 of 273 EX07 adult-evidence exclusions and 50 of 251 core reports with initially unestablished acquisition. Baseline, first-pass and reconciled judgments are retained separately. The 21 flagged core reports comprise 16 acquisition and seven age questions, with two overlapping reports. Sun confirmed the later seven age queries, 16 acquisition proposals and 239 eligibility results; there was no additional independent reviewer after him.

Audit stratum / endpoint	Human-confirmed first pass before discrepancy reconciliation	After source reconciliation	Interpretation
50 EX07 records: numerical adult evidence	50 unverifiable; 0 confirmed; 0 ineligible	50 unverifiable; no age-exclusion reversals	Retention 50/50: 100.0% (92.9-100.0); Wilson 95% CI.
50 core records: acquisition method	16 Manual; 34 not established	12 Manual; 38 not established	Initial baseline retention 34/50: 68.0% (54.2-79.2). Final retention 38/50: 76.0% (62.6-85.7).
50 core records: numerical adult evidence	43 confirmed; 5 ineligible; 2 unverifiable	50 confirmed with retained adult-subgroup/protocol restrictions	Sun-confirmed first pass: 43/50, 86.0% (Wilson 95% CI 73.8-93.0). The seven queries were resolved later; 50/50 after reconciliation is not the initial result. Masking is not established.
Current core and relaxed-frame totals (30 October)	Original baseline: 30 Manual; 251 not established	Current: 42 Manual (11.8%); 237 not established (66.8%) = 38 sampled/reconciled + 199 not acquisition-audited.	Current core + EX07: 434/629 not established (69.0%). Historical 50/273 and 50/251 sampling frames are unchanged.

The core age result confirmed by Jen-Tang Sun before discrepancy reconciliation was 43/50 (86.0%; Wilson 95% CI 73.8-93.0%), with five judged ineligible and two unverifiable, as clarified by the authors. It is not a tally of unvalidated provisional entries. Later source reconciliation retained all 50 with population restrictions and does not replace the first-pass 43/50 result. These are human-confirmed judgments in selected strata, not evidence of a blinded audit or map-wide inter-rater reliability. Each sampling stratum had a single baseline category, so Cohen's kappa is not used to imply agreement across all 871 reports. The 16 first-pass Manual classifications are distinct from the 12 subsequently supported by source reconciliation.

S9c. Origin of acquisition uncertainty in the sampled core records

Source-check category	Reports	Percent of 50	Interpretation
Recovered clinical acquisition evidence	12	24.0	Manual after source reconciliation: the earlier extraction had not established an acquisition method.
No explicit acquisition description in examined materials	29	58.0	The consulted materials did not describe how the pupil findings were acquired; unexamined supplements or other linked sources are not presumed to be missing information.
Ambiguous acquisition evidence	8	16.0	Includes four returned Manual proposals not supported by a sufficiently specific acquisition description (FT-0594, FT-1011, FT-0171, FT-0773).
Supporting material unavailable	1	2.0	FT-0010 remains unresolved because relevant supporting material was unavailable.
Total sampled core records	50	100.0	12 Manual plus 38 not established.

The 29-record category is limited to the materials actually examined; it is not a finding that every original article and supplement omitted acquisition details. At sampling, 201 of the 251 initially unestablished records were unsampled. The later eligibility audit excluded two of those 201, leaving 199 unsampled core records and all 50 sampled records. Of the sampled records, 12 were reclassified as manual and 38 remained unestablished. Thus the current 237 comprises 38 sampled/reconciled and 199 not acquisition-audited records. Sample fractions are not used to overwrite individual records. S9f instead reports a clearly conditional scenario under an explicit transferability assumption. The original 273 EX07 and 251 acquisition sampling frames remain preserved historical denominators.

S9d. Report-level resolution of the 21 flagged core reports

Original returned classifications are preserved below. The final column records current source-based reconciliation. Manual requires a described clinical pupil observation or qualitative light-response test; a named examiner or penlight model is not mandatory. A pupil category, generic examination heading or absence of standardized products alone is insufficient. Manual does not mean manual-only. Adult eligibility may use the complete baseline age-defined adult subset, but not outcome-selected cases.

Report	Original returned assessment	Current reconciliation and source limits
R006 / FT-0700	Age: confirmed; acquisition: manual	Age: confirmed; acquisition: Manual. Acquisition: The source links PLR to the clinical examination and describes the study's manual examinations and attending-physician acquisition. This is more specific than a stand-alone normal/abnormal label. The p.5 statement that most studies use manual examination would not be sufficient by itself, but the own-study p.7 limitation supplies the needed context. Sources: PDF p.2, Methods, Definition of good neurological outcome predictors; PDF p.7, Discussion limitations, first column; PDF p.7, same paragraph
R009 / FT-0446	Age: ineligible; acquisition: unestablished	Age: confirmed; acquisition: Not established. Adult scope: The complete age-defined adult subgroup: 17 of 18 original patients, ages 22-84. Exclude patient 7, aged 17. Age source: PDF p2 / printed p80, Materials and methods and Results; PDF p3 / printed p81, Tableau I (visually checked) Restrictions: Do not use the pooled 18-patient estimates as adult-only. The paper contains inconsistent GOS coding and pupil counts between prose and table; age confirmation does not resolve those quantitative extraction issues. Acquisition method is a separate assessment and is not changed by this age audit.
R011 / FT-0594	Age: confirmed; acquisition: manual	Age: confirmed; acquisition: Not established. Acquisition: Manual assessment is clinically plausible, but the actual acquisition link is not stated. The first quotation establishes a general neurological examination; the pupil-size item appears in the retrospective chart-abstraction list. Under the stated rule, connecting these two passages is an inference rather than an explicit pupil examination. The problem is not the absence of a named examiner or penlight. Sources: PDF p.2 / printed p.74, Patients and Therapeutic Protocol; PDF p.2 / printed p.74, Data Analysis; PDF p.2 / printed p.74, Data Analysis; PDF p.3 / printed p.75, Table 1 and Results; continuation p.4 / printed p.76
R013 / FT-0514	Age: ineligible; acquisition: manual	Age: confirmed; acquisition: Manual. Adult scope: All five adults identified at baseline in the 12-person monitored series, cases 8-12, ages 21, 24, 35, 47, 59. Age source: PDF p4 / printed p265, Patient material and methods; PDF p5 / printed p266, Table 1 (visually checked); PDF p5 / printed p266, Results, right column; PDF p6 / printed p267, opening Results continuation Restrictions: Do not define the eligible population as only the two patients who died; that would be outcome-selected. The age-defined population remains all five adults. Do not assume that the remaining three adult patients had normal pupils or reconstruct a complete 2 x 2 pupil-outcome table. This age verdict alone does not certify the report for quantitative pooling; incomplete pupil-specific denominators must be handled separately. Manual: Methods describe pupil/light-response signs in individual clinical assessment (PDF p.4, printed p.265), and Results explicitly describe pupils nonresponsive to light stimulation (PDF p.5, printed p.266). Exact examiner and light-source details are unspecified; other acquisition methods are not ruled out.

Report	Original returned assessment	Current reconciliation and source limits
R014 / FT-1155	Age: confirmed; acquisition: manual	Age: confirmed; acquisition: Manual. Acquisition: The report directly connects PLR to serial examinations conducted by specified clinician groups. This satisfies clinician-observed pupil examination without requiring a penlight brand or a named individual. Sources: PDF p.2 / printed p.94, Materials and Methods; PDF p.2 / printed p.94, next sentence
R017 / FT-0345	Age: unverifiable; acquisition: unestablished	Age: confirmed; acquisition: Not established. Adult scope: The 765-person CADISP report, supported by the directly linked parent-study numerical eligibility criterion of 18 years or older; this is protocol-based age evidence, not an observed minimum age. Age source: PDF p2 / printed p1654, Standard protocol approvals registrations and patient consents; PDF p2 / printed p1654, Methods and Results; Official ClinicalTrials.gov API, protocolSection.eligibilityModule.minimumAge; Official ClinicalTrials.gov API, protocolSection.statusModule.lastUpdatePostDateStruct Restrictions: This establishes a registered eligibility floor; it does not supply individual observed ages or independently prove compliance in every patient. The last-posted registry version was verified. No claim is made that an earlier 2008 version had an identical age field. No conflicting inclusion of minors was found in the checked report. If such evidence emerges, reconsider the classification.
R020 / FT-0481	Age: confirmed; acquisition: manual	Age: confirmed; acquisition: Manual. Acquisition: Pupil asymmetry is part of the prospectively performed clinical evaluation, and the report explicitly assigns evaluation to an investigator. This is not merely a pupil variable abstracted from an unspecified historical record. Taken together the passages support clinician observation, although the technique is not detailed. Sources: PDF p.2 / printed p.42, Patients and methods; PDF p.2 / printed p.42, same paragraph; PDF p.2 / printed p.42, randomization paragraph
R037 / FT-0590	Age: ineligible; acquisition: unestablished	Age: confirmed; acquisition: Not established. Adult scope: All 24 adults, ages 20-71, after removing the sole 16-year-old from the original 25-person series. Age source: PDF p1 / printed p85, Material and methods; PDF p2 / printed p86, Table 1 (visually checked); PDF p2 / printed p86, Table3 (visually checked) Restrictions: Label these adult data as reconstructed by the review team, not directly published adult-only results. Preserve the derivation and original tables; no mixed-age 25-person effect should be presented as adult-only. The eye-reaction categories combine pupil and oculocephalic details; extraction must preserve the actual categories.
R038 / FT-0783	Age: confirmed; acquisition: manual	Age: confirmed; acquisition: Manual. Acquisition: The source describes the original clinician evaluation and explicitly places pupil reactivity within its recorded baseline findings. Retrospective study design does not erase this original acquisition description. This supports manual clinical observation rather than a categorical-label-only inference. Sources: PDF p.2 / printed p.2, section 2.2 Study protocol; PDF p.2 / printed p.2, next sentence
R040 / FT-0452	Age: confirmed; acquisition: manual	Age: confirmed; acquisition: Manual. Acquisition: Pupils are expressly included in repeated clinical evaluations performed by an investigator. This provides a direct clinician-assessment link; the inference is not based on absence of a pupillometer name. Sources: PDF p.1 / printed p.1118, Abstract; PDF p.2 / printed p.1119, Randomization and evaluation; PDF p.2 / printed p.1119, same paragraph

Report	Original returned assessment	Current reconciliation and source limits
R041 / FT-0379	Age: confirmed; acquisition: manual	Age: confirmed; acquisition: Manual. Acquisition: The report explicitly describes pupil size/reaction assessment in ocular examinations and clinician observation of pupil reactions, rather than merely reporting pupil abnormalities. This supports manual clinical assessment. Sources: PDF p.2 / printed p.179, Materials and methods; PDF p.2 / printed p.179, follow-up paragraph; PDF p.2 / printed p.179, next sentence; PDF p.4 / printed p.181, Discussion
R042 / FT-1011	Age: confirmed; acquisition: manual	Age: confirmed; acquisition: Not established. Acquisition: This establishes that RAPD was assessed, but does not describe its acquisition, observer or light-test procedure. The other ophthalmic methods and anatomical inability to evaluate RAPD cannot uniquely establish manual rather than instrument-based acquisition. This is a verification/classification uncertainty, not a claim that the study used an automated device. Sources: PDF p.1, Methods; PDF p.3, Results; PDF p.4, Discussion
R053 / FT-0039	Age: ineligible; acquisition: unestablished	Age: confirmed; acquisition: Not established. Adult scope: The complete 12-adult subgroup, cases 2-13, ages 20-82; case 1, aged 16, excluded. Age source: PDF p4 / printed p230, Methods and Results; PDF p5 / printed p231, Table 1 (visually checked) Restrictions: Exclude the 16-year-old from adult-derived counts. The published 13-person multivariable regression is mixed-age and cannot be relabelled an adult-only result.
R063 / FT-0171	Age: confirmed; acquisition: manual	Age: confirmed; acquisition: Not established. Acquisition: A retrospective anisocoria category attributed to the first examination does not identify a manual observer, pupil measurement technique or non-automated test. This meets the instruction's example of a generic examination/category that should not alone establish acquisition. Sources: PDF p.4 / printed p.2058, Materials and methods; PDF p.5 / printed p.2059, section 3.4 Neurological status; PDF p.6 / printed p.2060, Table 3
R064 / FT-0021	Age: ineligible; acquisition: unestablished	Age: confirmed; acquisition: Not established. Adult scope: All eight adult patients, ages 25-75, individually identifiable in the 17-person series. Age source: PDF p1, abstract; PDF p3, Study population and demographics; PDF p4, Table 1 (visually checked) Restrictions: Do not use the mixed 17-patient estimates for adult synthesis. Six-month GOS is unavailable for adult cases 10 and 15; missing follow-up is not an age-verification failure. Acquisition method remains a separate question.
R070 / FT-0987	Age: confirmed; acquisition: manual	Age: confirmed; acquisition: Manual. Acquisition: The pupil findings are explicitly tied to the ophthalmologist's pupil examination and clinical determination of definite/questionable APD. This is more specific than a generic neurological assessment or pupil label alone, and supports a documented manual clinical assessment. It does not establish that no other acquisition method was used. Sources: PDF p.2 / printed p.4154, Materials and Methods; PDF p.2 / printed p.4154, Materials and Methods; PDF p.3 / printed p.4155, Results; PDF p.5 / printed p.4157, Results

Report	Original returned assessment	Current reconciliation and source limits
R071 / FT-0459	Age: unverifiable; acquisition: manual	Age: confirmed; acquisition: Manual. Adult scope: Restrict to the complete age >=50 subgroup within the main clinical comparison: 57 idiopathic/vasculopathic third-nerve-palsy patients plus 11 symptomatic PCoA-aneurysm patients, total 68. Do not claim the entire study cohort is numerically adult-confirmed. Age source: PDF p3 / printed p230, Table 1; PDF p4 / printed p231, Table2; PDF p6 / printed p233, Discussion, left column (visually checked); PDF p4 / printed p231, Results; PDF p6 / printed p233, Discussion Restrictions: Age confirmation applies only to the 68-person restricted comparison subset, not every patient in the study, and not all possible adults aged 18-49. Do not assert an exact complete diagnostic 2 x 2 table for this adult subset without resolving the missing pupil-assessment age stratum. The four other etiological cases listed in Table 1 are not part of the main 73-versus-15 comparison and must not silently be included in the 68-person denominator. Acquisition: A named investigator performed the clinical examination and specifically noted bilateral pupil size and direct/consensual light reactivity. This establishes manual clinical observation even without a named ruler or light source. Sources: PDF p.2 / printed p.229, Imaging Evaluations; PDF p.2 / printed p.229, Grading and Categorization; PDF p.2 / printed p.229, Grading and Categorization
R076 / FT-0773	Age: confirmed; acquisition: manual	Age: confirmed; acquisition: Not established. Unestablished: registry pupil categories and the Table 1 clinical-examination heading do not describe acquisition (PDF pp.2,4). Lack of standardized products (PDF p.6) is not proof of manual assessment. The AMPAS protocol (Zhao 2014, PDF pp.2-3) and linked 2016/2019 parent reports add no pupil-specific acquisition procedure. The 19-patient external-validation supplement was not obtained.
R077 / FT-0102	Age: confirmed; acquisition: manual	Age: confirmed; acquisition: Manual. Acquisition: The source specifically describes a pupil examination and response to projected light within clinical neurological examination, not merely a generic pupil code in a database. This supports a manual clinical assessment category, while leaving the precise stimulus, measuring tool and individual examiner unspecified. Sources: PDF p.2 / printed p.20, Data collection; PDF p.2 / printed p.20, Data collection
R087 / FT-0328	Age: confirmed; acquisition: manual	Age: confirmed; acquisition: Manual. Acquisition: Trained bedside nurses directly observed and recorded the named pupil sign repeatedly. This is explicit clinical-observer acquisition and supports manual, although the exact light source and test procedure are not specified. Sources: PDF p.2 / printed p.823, Data Collection; PDF p.5 / printed p.826, Limitations
R089 / FT-0366	Age: confirmed; acquisition: manual	Age: confirmed; acquisition: Manual. Acquisition: The detailed bedside clinical observations around the procedure, pupil findings and associated elicited neurological signs support manual clinical assessment, not merely a retrospective pupil category. The exact acquisition device is not reported; this must not be converted into a claim that no pupillometer was used. Sources: PDF p.2 / printed p.190, Methods; PDF p.2 / printed p.190, Results; PDF p.6 / printed p.194, Limitations

All seven age resolutions retain their original core eligibility and explicitly constrain the supported population. FT-0345 uses the directly linked CADISP minimum age of 18 years (NCT00657969), not a claim that an observed minimum was reported in the article. FT-0514 retains all five baseline-defined adults, not only the two who died; pupil-specific data remain incomplete. Whole mixed-age estimates, incomplete adult outcomes and unavailable pupil denominators are not cleared for quantitative pooling by this audit. The original returned form is not overwritten.

S9e. Eligibility-only technical audit of the 239 acquisition-unestablished reports

After the 29 October acquisition reconciliation, all 239 reports still classified as acquisition Not established were already included in the core map; they were not 239 pending screening decisions. A Codex-assisted technical eligibility audit on 30 October evaluated saved numerical-age evidence, acute clinical pupil relevance, design, source identity and restrictions. All 239 source files were available and matched their recorded SHA256 values. Twenty-one reports received targeted rereading of recorded original-source pages; the other 218 were checked using preserved source-grounded evidence. Jen-Tang Sun personally validated the evidence and final eligibility decisions, as confirmed by the authors on 4 November 2026. That author confirmation does not imply that all 239 complete PDFs were newly read, and it does not newly establish acquisition methods. File availability and hash agreement are distinct from review depth.

Audit item	Reports	Meaning
Eligible without a newly imposed restricted adult subset (IN01)	191	Retain current inclusion, with existing source/metric limitations.
Complete restricted adult subgroup (IN02)	46	Retain only the explicit eligible subgroup; no mixed-age parent effects.
Adult eligibility numerically unverified (EX07)	1	FT-0120; later ICU disposition cannot supply baseline adult eligibility.
Inseparable mixed-age population (EX02)	1	UF-43BA3EE5; response-selected adult nonresponders cannot supply a complete baseline adult cohort.
Total audited	239	237 retained; two eligibility exclusions.
Targeted original-source recheck	21	Only explicitly documented pages newly consulted.
Preserved source-evidence audit	218	No assertion of a new full-text reading.

The two changes reduce the core from 357 to 355 and current unestablished acquisition from 239 to 237; criteria exclusions increase from 495 to 497. EX07 increases from 273 to 274, so the deliberately relaxed core-plus-EX07 frame is 629, not 630. The 15 qualitative/linkage records, three unresolved-source closures and one duplicate remain unchanged. Neither changed report belongs to the original 100-record random audit; its 50/273 and 50/251 frames and observed audit results therefore remain unchanged. These targeted eligibility proportions are not map-wide agreement coefficients, and no new kappa is calculated. The other 632 historical-frame records were not re-adjudicated in this exercise.

Report	Current decision	Reason and restriction
FT-0120	Exclude; EX07	The 122-patient ketamine-intoxication cohort has only a mean age of 26.7 years (SD 6.5), without a numerical adult entry criterion or observed full age range. The seven individually aged ICU patients were selected by disposition after emergency care, explicitly listed as an outcome. This subsequent-outcome subgroup cannot establish adult eligibility for the report under the current rule. Adult eligibility is unverified; this is not evidence of confirmed paediatric enrollment. Source: PDF p.2 / printed p.7, Methods: disposition after emergency care; PDF p.3 / printed p.8, Table 1: ICU admission under Desenlace; PDF p.7 / printed p.12, Table 2: seven ICU cases

Report	Current decision	Reason and restriction
UF-43BA3EE5	Exclude; EX02	The report evaluates 43 naloxone recipients. The 28 treatment responders include ages 14-83, while the 15 nonresponders are 33-89. Group assignment was determined by improvement after naloxone. Selecting only adult nonresponders cannot establish an adult baseline cohort, and adult pupil findings from the mixed-age responders cannot be separated. The source documents mixed ages, unlike an age-unverified exclusion. Source: PDF p.2 / printed p.447, Methods and Results: response-defined allocation; PDF p.3 / printed p.448, Table 4: group age ranges; PDF p.4 / printed p.449: chart documentation limitations

FT-0133 retains its complete separately reported age >=65 subgroup; the original Figure 3 label confirms >=65 and the earlier transcription discrepancy is removed. FT-0637 retains only its complete originally defined 37-person adult recovery report, with no inference about the 125-person parent population (S3). Full before/after records, exact consulted pages, source hashes and reversibility conditions are preserved in Eligibility_Audit_239_20261030 and the dated master fields. The source-specific restrictions do not clear any report for pooling. Search-provenance gaps, unintegrated external candidates and uncertain cohort overlap remain unresolved limitations.

S9f. Verification coverage and conditional acquisition sensitivity scenario

The recorded 237/355 (66.8%) is the proportion carrying the current not-established label. It combines two different verification depths and is not a fully audited prevalence estimate. The sampled and unsampled contributions are shown separately below. The 239-record technical eligibility check does not convert the 199 unsampled records into acquisition-audited records.

Current verification status	Reports	Percent of 355	Interpretation
Sampled and reconciled; acquisition still not established	38	10.7	Observed source-reconciliation result within the additional acquisition sample.
Not acquisition-audited; previous not-established label retained	199	56.1	Outside the additional acquisition sample; later eligibility checks did not establish acquisition.
Current not-established total	237	66.8	Ledger total, not a uniformly audited estimate.
Sampled and reconciled; manual acquisition recovered	12	3.4	Observed reclassification within the sample; included in the 42 current Manual records.

Under the conditional assumption that the 199 unsampled retained records would have the same residual nonresolution fraction as the reconciled sample, $q = 38/50 = 0.76$, the scenario is $(38 + 199q)/355 = 53.3\%$. The Wilson 95% limits for the sample nonresolution fraction are 62.6%-85.7%. Substituting those limits into the same expression gives a scenario range of 45.8%-58.7%. This transformed range is not a confidence interval for a newly observed prevalence across all 355 reports. It assumes comparable verification yield in sampled and unsampled retained records, treats the reconciled sample outcomes as fixed, and does not include uncertainty in reconciliation or in other acquisition categories.

No report is reclassified on the basis of this calculation. The observed ledger remains 38 sampled/reconciled plus 199 not acquisition-audited not-established records. The scenario is presented alongside those counts to show how unresolved verification could affect the headline proportion; it does not estimate clinical adoption, field-wide prevalence, or omissions in original publications. Supplementary Data File 1 includes the row-level sampling identifiers, verification-status fields and executable calculation; detailed original source-verification records are available on request.
