# Supplementary material for: An Evidence Map of Pupillary Assessment in a Preselected Acute-Care Literature Archive
Source: Diagnostics (Basel). 2026 Nov 9;16(21):3698. doi: 10.3390/diagnostics16213698 (PMC13761962; doi:10.3390/diagnostics16213698)
Supplement: Supplementary file 1 [file diagnostics-16-03698-s001.zip › 04_S4_Report_Decision_Index.pdf]

Supplementary Table S4

An Evidence Map of Pupillary Assessment in a Preselected Acute-Care Literature Archive

Complete index of 871 historical report records. Current dispositions: 355 core, 15 qualitative/linkage only, 497 criteria exclusions, three unresolved-source closures and one duplicate alias. Full current reasons, source locations, adult restrictions and historical fields are preserved in the accompanying searchable HTML and machine-readable records.

The 30 October eligibility-only audit retained 237 of the 239 acquisition-unestablished reports and excluded FT-0120 (EX07) and UF-43BA3EE5 (EX02). Historical human review is preserved; 21 reports received targeted source rereading and 218 had preserved-evidence audits. This is not a new full-text read of all 239 reports. FT-0133 Figure 3 confirms age >=65; its eligibility is unchanged.

FT-0671 and FT-0691 remain core with numerical-reuse restrictions. FT-1174 is BOX report linkage only. Acquisition Not established is not itself an exclusion or a verified absence of a method. Complete restricted adult subgroups must not be represented by whole mixed-age parent results.

Report ID	Author / year	Title	Current disposition	Identifiers
FT-0001	Wiefhoff, Jan; 2026	Incidence and impact of intracranial complications in patients undergoing extracorporeal membrane oxygenation treatment	Exclude; EX07	PMID 42382653 DOI 10.1016/j.bas.2026.106123
FT-0003	Gourier, Sylvain; 2026	Impact of sex on outcomes in critically ill patients with subarachnoid hemorrhage: a secondary analysis of a French prospective cohort study.	Core; IN01	PMID 42204647
FT-0007	Sarac, Mustafa Emre; 2026	From Neurological Severity to Anatomical Burden: An Integrated Clinical-CT Model for Predicting In-Hospital Mortality in Adults with Penetrating Cranial Gunshot Injuries.	Core; IN01	PMID 42072871
FT-0008	Andersson, Alice S; 2026	Favourable outcome in a subset of ultra-severe traumatic brain injury patients - a single-centre retrospective study.	Exclude; EX02_POPULATION	PMID 42058275
FT-0009	Rolland, David; 2026	Association between invasive intracranial pressure monitoring and 1-year functional outcome in ICU patients with severe stroke: a pre-planned ancillary study of the SPICE prospective cohort (SPICE-ICP).	Core; IN01	PMID 41881341
FT-0010	Lunz, Dirk; 2026	Prehospital initiation of extracorporeal life support for refractory out-of-hospital cardiac arrest-results of a prospective observational study.	Core; IN01	PMID 41857661
FT-0012	Mansvelder, Floor J; 2026	Prehospital Cardiopulmonary Resuscitation in Patients with Suspected Severe Traumatic Brain Injury: A BRAIN PROTECT Sub-Analysis.	Exclude; EX02	PMID 41682614
FT-0013	Sarabbadieh, Armin Mansouri; 2026	Investigation of serum concentration and the type of opioid used in patients with opium poisoning.	Core; IN01	PMID 41675972

Report ID	Author / year	Title	Current disposition	Identifiers
FT-0014	Schuller, Diana; 2026	Predictive Accuracy of Glasgow Coma Scale and Pupillary Data on Presence of Traumatic Brain Injury.	Exclude; EX07	PMID 41598635
FT-0015	Gopakumar, Sricharan; 2026	Prognostication of civilian gunshot wounds to the head: the Baylor score.	Core; IN02	PMID 41569760
FT-0016	Klavansky, Dana; 2025	A Multimodal Non-invasive Approach for Intracranial Pressure Assessment: A Single-Center Study.	Core; IN	PMID 41510443
FT-0017	Tegegne, Belachew; 2025	Management outcome of epidural hematoma patients at Dessie Comprehensive Specialized Hospital: one-year prospective observational study.	Exclude; EX07	PMID 41394170
FT-0019	Peng, Jinxiu; 2025	Predictive modeling of brain herniation risk factors and critical thresholds in spontaneous intracerebral hemorrhage: a pilot study.	Exclude; EX03	PMID 41255783
FT-0020	Kaaniche, Fatma Medhioub; 2025	Acute drug intoxication: predictive factors for hospitalization.	Exclude; EX02_POPULATION	PMID 41246242
FT-0021	Benko, Zsolt; 2025	The role of decompressive craniectomy in the management of intracranial infections: a single-center 15-year retrospective study.	Core; IN02	PMID 41175411
FT-0024	Hengartner, Astrid C; 2026	Withdrawal of life-supporting treatment in severe traumatic brain injury.	Exclude; EX07	PMID 40939206
FT-0026	Gonzalez, Hector Jr; 2025	Pupillometry in the Emergency Department: A Tool for Predicting Patient Disposition.	Core; IN	PMID 40794983
FT-0027	Peng, Guoyi; 2025	Development and validation of a novel scoring model for predicting underlying intracranial atherosclerosis prior to endovascular treatment in acute posterior circulation large-vessel occlusion.	Core; IN01	PMID 40771984
FT-0028	Oktay, K; 2025	Evaluation of Prognostic Factors in Patients with Cranial Gunshot Wounds.	Exclude; EX02_POPULATION	PMID 40717052
FT-0029	Vattipally, Vikas N; 2025	Operative neurosurgery for traumatic subdural hematoma: association between trauma center variation and patient outcomes.	Core; IN01	PMID 40712171
FT-0030	Yasuda, Masaki; 2025	Prognostic Factors and Initial Treatment Strategies for Patients with Head Trauma and Vital Signs of Shock.	Exclude; EX02_POPULATION	PMID 40697805
FT-0031	Vattipally, Vikas N; 2026	Interhospital Variation in Operative Intervention for Firearm-Related Penetrating Traumatic Brain Injury and Associations With Inpatient Mortality.	Core; IN01	PMID 40673687
FT-0032	Kumar, Abhinav; 2026	Clinical determinants for survival following emergency thoracotomy in trauma patients: An 8-year experience from a level 1 trauma center.	Exclude; EX03	PMID 40610304
FT-0033	Beekman, Rachel; 2025	The impact of pupil diameter on assessing illness severity and outcome after out-of-hospital cardiac arrest in patients with unreactive pupils.	Core; IN01	PMID 40582484

Report ID	Author / year	Title	Current disposition	Identifiers
FT-0034	Yamaguchi, Junko; 2025	Pupillary Responses and Vital Signs in Hypoglycemic Patients with Impaired Consciousness During Prehospital Care: A Retrospective Observational Study.	Exclude; EX07	PMID 40564809 DOI 10.3390/diagnostics15121487
FT-0037	Egas Teran, Maria Ines; 2025	Time-to-treatment in traumatic brain injury: unraveling the impact of early surgical intervention on patient outcomes.	Core; IN01	PMID 40483551
FT-0038	Savi, Marzia; 2025	Validation of the SHELTER score after aneurysmal subarachnoid hemorrhage cohort: An observational retrospective study.	Core; IN01	PMID 40449256
FT-0039	Baig, Azam Ali; 2026	Cranial gunshot wounds: comparative analysis of management and outcomes in a United Kingdom Major Trauma Centre and the United States.	Core; IN02	PMID 40353337
FT-0040	Veerapaneni, Divya; 2025	Early Pupil Abnormality Frequency Predicts Poor Outcomes and Enhances International Mission for Prognosis and Analysis of Clinical Trials in Traumatic Brain Injury (IMPACT) Model Prognostication in Traumatic Brain Injury.	Exclude; EX07	PMID 40299976
FT-0041	Eric Nyam, Tee-Tau; 2025	Age and pupil size: key predictors of mortality in traumatic brain injury patients with GCS 3.	Core; IN01	PMID 40255893
FT-0042	Liu, Jiaqi; 2025	Timing of pharmacological venous thromboembolism prophylaxis after firearm-related penetrating brain injury.	Exclude; EX07	PMID 40184674
FT-0043	Haman, Nassourou Oumarou; 2025	Predictors of poor outcomes in patients with traumatic brain injury at the University Teaching Hospital of Bamenda, Cameroon.	Exclude; EX02_POPULATION	PMID 40057998
FT-0044	Devalckeneer, Antoine; 2025	Predictive outcome factors in the treatment of subarachnoid hemorrhage with hematoma caused by ruptured anterior circulation aneurysms: A monocentric experience.	Core; IN01	PMID 40022867
FT-0045	Gmeiner, Raphael; 2025	Impact of interhospital transport on outcome in traumatic epidural hematoma: experiences of a level-1 trauma center.	Exclude; EX02	PMID 39918624
FT-0046	Shen, Jun; 2025	Remote Site Delayed Extradural Hematoma Secondary to Decompressive Craniectomy for Traumatic Brain Injury: Incidence, Predictors, Characteristics, and Outcomes	Exclude; EX02	PMID 39913905 DOI 10.1097/SCS.0000000000011124
FT-0047	Rana, Chahat; 2025	Neuroradiological Correlates of Abnormal Pupillary Light Reflex Findings Among Patients in the Neuroscience Intensive Care Unit	Exclude; EX07	PMID 39883016 DOI 10.1097/JNN.0000000000000818
FT-0048	Aziz, Peter; 2025	Is Intracranial Pressure Monitoring After Open Cranial Procedures Associated With Outcome?	Exclude; EX07	PMID 39842048
FT-0049	Vattipally, Vikas N; 2025	Latent Variable Analysis of Demographic and Clinical Drivers of Care Intensity Before Palliative Care Consultation Among Older Adult Patients with Traumatic Brain Injury.	Core; IN01	PMID 39821567
FT-0050	Song, Li; 2025	A nomogram for predicting prognostic risk factors in individuals with poor grade aneurysmal subarachnoid hemorrhage: a retrospective study.	Core; IN01	PMID 39775311

Report ID	Author / year	Title	Current disposition	Identifiers
FT-0052	Kim, Ji-Na; 2025	Outcomes of Acute Subdural Hematoma in Adults with a Glasgow Coma Scale Score of 3 : An Analysis from Five Regional Trauma Centers.	Core; IN01	PMID 39721575
FT-0053	Zellner, Tobias; 2024	Munich cCT Rule for Patients with Recreational Drug and Ethanol Poisoning.	Exclude; EX07	PMID 39685555
FT-0054	Alqirnas, Muhannad Q; 2025	The Impact of Intoxication on the Prognosis of High-Speed Motor Vehicle Accidents: A Tertiary Care Center Experience.	Core; IN01	PMID 39670240
FT-0055	Wu, Ruhong; 2024	Risk factor analysis and nomogram model establishment for in-hospital death of elderly patients with surgically treated traumatic acute subdural hematoma.	Core; IN01	PMID 39654253
FT-0056	Hosseininejad, Seyed M; 2024	The clinico-epidemiological, diagnostic, and therapeutic aspects of methadone poisoning: a registry-based clinical study.	Exclude; EX07	PMID 39649894
FT-0057	Modi, Priyanka; 2024	Correlation of Point of Care Ultrasound (POCUS) Guided Pupillary Assessment Parameter with Glasgow Coma Scale in Patients with Altered Mental Status- A Cross Sectional Study.	Core; IN	PMID 39634693
FT-0059	Jalawadi, Vishwanath Malkappa; 2024	Managing Amitraz Poisoning: Epidemiological Data and Clinical Insights From a Tertiary Care Hospital in Southern India.	Exclude; EX02_POPULATION	PMID 39628755
FT-0060	Tentis, Morgan; 2024	Predictors of End-of-Life Care in Nonelderly Adults With Severe Traumatic Brain Injury.	Core; IN01	PMID 39613514
FT-0062	Castano-Leon, Ana M; 2024	The impact of early surgery on mortality and functional recovery in older adults with traumatic intracranial lesions: a propensity score-based analysis.	Core; IN01	PMID 39503799
FT-0065	Ding, Xiaohu; 2025	Clinical spectrum and possible pathogenesis of progressive outer retinal necrosis.	Core; IN01	PMID 39237291
FT-0069	Greil, Madeline E; 2024	Extracranial Complications in Monitored and Nonmonitored Patients with Traumatic Brain Injury in the BEST TRIP Trial and a Companion Observational Cohort.	Exclude; EX07	PMID 39069132
FT-0071	Schroder, Isabelle; 2024	Single-centre real-life observational study on mortality and outcomes: decompressive craniectomy and brain death in traumatic brain injury, haemorrhage, and other cerebral diseases.	Core; IN01	PMID 38969875
FT-0072	Chongolo, Anna M; 2024	Predictors of 30-day mortality following the first episode of stroke among patients admitted at referral hospitals in Dodoma, central Tanzania: A prospective longitudinal observational study.	Core; IN01	PMID 38933419
FT-0073	Doyle, Brittany R; 2024	Anisocoria After Direct Light Stimulus is Associated with Poor Outcomes Following Acute Brain Injury.	Exclude; EX07	PMID 38918339
FT-0074	Dantan, E; 2024	Long-term survival of traumatic brain injury and intra-cerebral haemorrhage patients: A multicentric observational cohort.	Exclude; EX07	PMID 38875914

Report ID	Author / year	Title	Current disposition	Identifiers
FT-0075	Rodriguez-Vazquez, Alejandro; 2025	Computed tomography perfusion as an early predictor of malignant cerebral infarction.	Exclude; EX03	PMID 38872264
FT-0076	Promrungsri, Puangpak; 2024	Clinical features of seven patients poisoned with a tolfenpyrad-based insecticide in Thailand.	Core; IN02	PMID 38856367
FT-0077	Ahmed, Nasim; 2025	Surgical Outcomes of Epidural Hematoma in Trauma Patients with Absent Pupillary Reactions: A National Trauma Data Analysis.	Core; IN01	PMID 38821065
FT-0080	Ramamoorthy, Thirumoorthy; 2024	Diagnostic Value of Point-of-Care Ultrasound-Guided Assessment of Relative Afferent Pupillary Defect in Adult Ocular Trauma Patients Presenting to the Emergency Department: A Prospective Cohort Study.	Core; IN	PMID 38581178
FT-0081	Park, Jung Hwan; 2024	The Efficacy of Traumatic Brain Injury Treatment by Neurotrauma Specialists.	Exclude; EX07	PMID 38576504
FT-0082	Aaron, Sanjit; 2024	Outcomes of Decompressive Surgery for Patients With Severe Cerebral Venous Thrombosis: DECOMPRESS2 Observational Study.	Exclude; EX07	PMID 38572636
FT-0083	Zewdu, Melaku; 2024	Incidence of intraoperative hypotension and its factors among adult traumatic head injury patients in comprehensive specialized hospitals, Northwest Ethiopia: a multicenter observational study.	Core; IN01	PMID 38561657
FT-0084	Fonseca, Ricardo A; 2024	Prolonged hourly neurological examinations are associated with increased delirium and no discernible benefit in mild/moderate geriatric traumatic brain injury.	Exclude; EX03	PMID 38509046
FT-0086	Khalid, Shah; 2023	Mortality And Prognostic Factors In Patients Operated For Acute Epidural Hematoma.	Exclude; EX02_POPULATION	PMID 38406908
FT-0089	Teshita, Gemechu; 2024	Clinical characteristics and functional outcome of surgically treated adult head trauma patients with acute subdural hematoma: Ethiopian tertiary hospitals experience.	Exclude; EX02_POPULATION	PMID 38260115
FT-0090	Chilakapati, Sai; 2024	Use of MRI in patients with severe diffuse traumatic brain injury: A matched National Trauma Data Bank analysis	Exclude; EX07	PMID 38196125 DOI 10.1097/TA.0000000000004255
FT-0091	Gomila Muniz, Isabel; 2024	Retrospective analysis of venlafaxine-induced hypoglycemia in patients with overdose.	Exclude; EX07	PMID 38182480
FT-0092	Koipapi, Sengua; 2024	Outcomes of traumatic brain injury patients with acute epidural and subdural hematoma who underwent burr hole surgery: A two-year study at Kilimanjaro Christian Medical Centre, Tanzania.	Core; IN01	PMID 38090192
FT-0093	Kamaludin, Ahmad I; 2024	Evaluation of severe traumatic brain injury referrals to the National Tertiary Neurosurgical Centre in the Republic of Ireland.	Core; IN01	PMID 38071143
FT-0094	Hofmann, Bjorn B; 2024	Clinical Outcome Prediction of Early Brain Injury in Aneurysmal Subarachnoid Hemorrhage: the SHELTER-Score.	Core; IN01	PMID 38030877

Report ID	Author / year	Title	Current disposition	Identifiers
FT-0095	Suehiro, Eiichi; 2023	Retrospective Observational Study of Patients With Subdural Hematoma Treated With Idarucizumab.	Core; IN01	PMID 38028276
FT-0096	Oyemolade, Toyin Ayofe; 2024	Outcome of Nonoperative Management of Selected Cases of Acute Traumatic Intracranial Hematomas in a Rural Neurosurgical Service of a Developing Country.	Exclude; EX07	PMID 37995994
FT-0098	Shen, Hanchao; 2023	Risk factors of prognosis in older patients with severe brain injury after surgical intervention.	Core; IN01	PMID 37925438
FT-0101	Griepp, Daniel W; 2024	Emergency decompressive surgery in patients with transtentorial brain herniation and pupillary abnormalities: the importance of improved pupillary response after osmotherapy and surgery.	Core; IN	PMID 37548576
FT-0102	Tongyoo, S; 2023	Prevalence and independent predictors of in-hospital stroke among patients who developed acute alteration of consciousness in the medical intensive care unit: A retrospective case-control study.	Core; IN	PMID 37521958
FT-0103	Lambeck, Johann; 2023	[Prospective investigation of extracranial duplex sonography for the detection of cerebral circulatory arrest in patients with irreversible loss of brain function].	Exclude; EX07	PMID 37477664
FT-0104	Westphal, Laura Philine; 2022	Sex-related differences in symptom presentation of patients with aneurysmal subarachnoid hemorrhage.	Exclude; EX07	PMID 37476819
FT-0105	Ang, Darwin; 2023	Data-Driven Blood Transfusion Thresholds for Severely Injured Patients During Blood Shortages.	Exclude; EX07	PMID 37331188
FT-0107	Lang, Lijian; 2023	An independently validated nomogram for individualised estimation of short-term mortality risk among patients with severe traumatic brain injury: a modelling analysis of the CENTER-TBI China Registry Study.	Exclude; EX07	PMID 37180469
FT-0108	Zacchetti, Lucia; 2023	Clinical frailty scale as a predictor of outcome in elderly patients affected by moderate or severe traumatic brain injury.	Core; IN01	PMID 37090991
FT-0109	Greuter, Ladina; 2023	Mortality of Surgically Treated Neurotrauma in Elderly Patients and the Development of a Prediction Score: Geriatric Neurotrauma Mortality Score.	Core; IN01	PMID 37054949
FT-0111	Jakobsen, Elisabeth Waldemar; 2023	Brimonidine eye drops reveal diminished sympathetic pupillary tone in comatose patients with brain injury.	Core; IN	PMID 37014450
FT-0113	Nassrallah, Georges; 2024	Incidence of ophthalmologic pathology and associated risk factors in orbital fractures at a level I trauma centre.	Exclude; EX02_POPULATION	PMID 37001561
FT-0114	Mansour, Ali; 2023	Comparative Effectiveness of Intracranial Pressure Monitoring vs No Monitoring in Severe Penetrating Brain Injury Management.	Exclude; EX07	PMID 36961466

Report ID	Author / year	Title	Current disposition	Identifiers
FT-0115	Kam, Jeffrey K P; 2023	Outcomes after inter-hospital transfer of intensive care patients with haemorrhagic stroke: a 5-year retrospective review.	Core; IN01	PMID 36947603
FT-0117	Zhou, Jingyi; 2023	Prognostic models for survival and consciousness in patients with primary brainstem hemorrhage.	Exclude; EX07	PMID 36908614
FT-0118	Lim, Jia Xu; 2023	Outcomes of decompressive craniectomy for large territory cerebral infarction with and without prior reperfusion: a multicentre retrospective review.	Core; IN01	PMID 36808008
FT-0119	Barea-Mendoza, Jesus Abelardo; 2023	External validation of the Glasgow Coma Scale-Pupils in patients with severe head injury.	Exclude; EX07	PMID 36756915
FT-0120	Gomila Muniz, Isabel; 2023	Ketamine detection in urine samples from patients poisoned by recreational drugs: epidemiologic, clinical, and toxicologic profiles.	Exclude; EX07	PMID 36756911
FT-0121	El Zahran, Tharwat; 2023	The yield of neuroimaging in patients presenting to the emergency department with isolated neuro-ophthalmological complaints: A retrospective chart review.	Exclude; EX02_POPULATION	PMID 36705369
FT-0122	Sweeney, Jared; 2023	Risk of Neurological Decline in Patients With Temporal Lobe Brain Masses.	Core; IN01	PMID 36697071
FT-0123	Beniwal, Hemant Kumar; 2022	Prognostic Value of Swirl Sign in Acute Epidural Hemorrhage.	Exclude; EX02_POPULATION	PMID 36644411
FT-0125	Dos Santos, Josefa Cristina Pereira; 2023	Pesticide exposure and poisoning in Brazil: Outcome severity, clinical manifestations and management of cases reported to a poison control center.	Exclude; EX02_POPULATION	PMID 36573257
FT-0126	Wu, Li; 2023	Cortico-hypothalamic pathway of Horner syndrome derived from isolated lenticulostriate stroke.	Core; IN	PMID 36507977
FT-0127	Groshi, A G M; 2022	Factors influencing the outcomes of patients with severe traumatic brain injury following road traffic crashes.	Exclude; EX02_POPULATION	PMID 36477057
FT-0128	Choi, Dae Han; 2022	Clinical Outcome of Patients Diagnosed Traumatic Intracranial Epidural Hematoma With Severe Brain Injury (Glasgow Coma Scale </=8) Who Undergo Surgery: A Report From the Korean Neuro-Trauma Data Bank System.	Core; IN01	PMID 36381437
FT-0129	Mkubwa, J J; 2022	Traumatic brain injury: Association between the Glasgow Coma Scale score and intensive care unit mortality.	Core; IN01	PMID 36101711
FT-0130	Griepp, Daniel W; 2022	Bilaterally Fixed and Dilated Pupils Are Not the Kiss of Death in Patients with Transtentorial Herniation: A Single Surgeon's Experience.	Core; IN02	PMID 35964901
FT-0131	Moyer, Jean-Denis; 2022	Machine learning-based prediction of emergency neurosurgery within 24 h after moderate to severe traumatic brain injury.	Core; IN01	PMID 35922831

Report ID	Author / year	Title	Current disposition	Identifiers
FT-0132	Krueger, Evan M; 2023	Simple wound closure compared with surgery for civilian cranial gunshot wounds.	Core; IN01	PMID 35901757
FT-0133	Borazjani, Roham; 2022	Current Status and Outcomes of Critical Traumatic Brain Injury (GCS = 3-5) in a Developing Country: A Retrospective, Registry-Based Study.	Core; IN02	PMID 35789431
FT-0134	Kolias, Angelos G; 2022	Evaluation of Outcomes Among Patients With Traumatic Intracranial Hypertension Treated With Decompressive Craniectomy vs Standard Medical Care at 24 Months: A Secondary Analysis of the RESCUEicp Randomized Clinical Trial.	Exclude; EX07	PMID 35666526
FT-0136	Pansell, Jakob; 2022	Can Quantitative Pupillometry be Used to Screen for Elevated Intracranial Pressure? A Retrospective Cohort Study	Exclude; EX07	PMID 35606562 DOI 10.1007/s12028-022-01518-y
FT-0137	Wu, Ruhong; 2022	Analysis of the risk factors for intraoperative acute diffuse brain swelling in patients with isolated traumatic acute subdural haematomas.	Core; IN01	PMID 35568840
FT-0138	Trevisi, Gianluca; 2024	The role of the craniotomy size in the surgical evacuation of acute subdural hematomas in elderly patients: a retrospective multicentric study.	Core; IN01	PMID 35380204
FT-0140	Yang, Chun; 2022	Intracranial Pressure Monitoring in the Intensive Care Unit for Patients with Severe Traumatic Brain Injury: Analysis of the CENTER-TBI China Registry.	Exclude; EX07	PMID 35246788
FT-0141	Raghib, Muhammad Faraz; 2022	Risk Factors and Outcomes of Redo Craniotomy: A Tertiary Care Center Analysis.	Exclude; EX02_POPULATION	PMID 35223225
FT-0142	Skulberg, Arne Kristian; 2022	Comparison of intranasal and intramuscular naloxone in opioid overdoses managed by ambulance staff: a double-dummy, randomised, controlled trial.	Core; IN01	PMID 35137493
FT-0143	On, Sophia; 2022	The burden and risks factors for intracerebral hemorrhage in a Southeast Asian population.	Exclude; EX07	PMID 35114482
FT-0144	Singh, Prachi; 2021	Identification of abnormal pupil dilation velocity as a biomarker of cerebral injury in neurocritically ill patients.	Exclude; EX07	PMID 35107552
FT-0146	Huckhagel, T; 2022	Cranial nerve injuries in patients with moderate to severe head trauma - Analysis of 91,196 patients from the TraumaRegister DGU(R) between 2008 and 2017.	Exclude; EX02_POPULATION	PMID 34902753
FT-0147	Servia, Luis; 2021	Development of a new score for early mortality prediction in trauma ICU patients: RETRASCORE.	Exclude; EX07	PMID 34876199
FT-0148	Maksimovic, Zana M; 2023	Acute organophosphate and carbamate pesticide poisonings - a five-year survey from the National Poison Control Center Of Serbia.	Exclude; EX02_POPULATION	PMID 34875948
FT-0149	Goncalves, Ruben; 2022	Involuntary MDMB-4en-PINACA intoxications following cannabis consumption: clinical and analytical findings.	Exclude; EX02_POPULATION	PMID 34850659
FT-0150	Nazerian, Peiman; 2022	Comparison of out of hospital cardiac arrest due to acute brain injury vs other causes.	Exclude; EX07	PMID 34798571

Report ID	Author / year	Title	Current disposition	Identifiers
FT-0151	Wang, Changyi; 2022	Early Prediction of Malignant Edema After Successful Recanalization in Patients with Acute Ischemic Stroke.	Exclude; EX03	PMID 34751418
FT-0152	Mummadi, Mahesh Kumar; 2021	Investigation of an acute neurological outbreak in Eluru, India, 2020.	Core; IN02	PMID 34748567
FT-0154	Shaaban, Ahmed; 2021	Retrospective analysis of the surgical management of spontaneous supratentorial intracerebral hemorrhage: A single-center study.	Exclude; EX07	PMID 34712600
FT-0155	Amare, Abraham Tsedalu; 2021	Survival status and predictors of mortality among traumatic brain injury patients in an Ethiopian hospital: A retrospective cohort study.	Exclude; EX07	PMID 34703730
FT-0156	Benveniste, Ronald; 2022	Intracranial Hypertension After Primary Decompressive Craniectomy for Head Trauma.	Core; IN01	PMID 34656793
FT-0157	Prescott BR; 2022	Anisocoria and Poor Pupil Reactivity by Quantitative Pupillometry in Patients With Intracranial Pathology.	Exclude; EX07	PMID 34637415 DOI 10.1097/CCM.0000000000005272
FT-0158	Nyancho, Daniel; 2021	Anisocoria Correlates With Injury Severity and Outcomes After Blunt Traumatic Brain Injury.	Exclude; EX07	PMID 34620803
FT-0159	Akkina, Sarah R; 2024	Factors Associated with Ocular Injury in Orbital Fracture Patients: Who Requires Urgent Ophthalmic Evaluation?	Core; IN01	PMID 34569815
FT-0161	Chang, Tao; 2021	Risk Factors and Neurologic Outcomes in Patients with Traumatic Brain Injury and Coagulopathy Within 72 h After Surgery.	Core; IN01	PMID 34531657
FT-0162	Bocca, Leonardo Favi; 2021	Traumatic acute subdural hematoma and coma: retrospective cohort of surgically treated patients.	Core; IN01	PMID 34513187
FT-0163	Yoshida, Shinsuke; 2021	Best Motor Response Predicts Favorable Outcome for "True" WFNS Grade V Patients with Aneurysmal Subarachnoid Hemorrhage.	Exclude; EX07	PMID 34481320
FT-0164	van Veen, Ernest; 2021	Occurrence and timing of withdrawal of life-sustaining measures in traumatic brain injury patients: a CENTER-TBI study.	Exclude; EX07	PMID 34351445
FT-0165	Pattnaik, Shradha; 2021	Spectrum of Ocular Findings in Closed Head Injuries, Correlation With Severity of Neurological Involvement, and Treatment Outcome: A Hospital-Based Cross-Sectional Study.	Core; IN01	PMID 34295601
FT-0166	Robba, Chiara; 2021	Intracranial pressure monitoring in patients with acute brain injury in the intensive care unit (SYNAPSE-ICU): an international, prospective observational cohort study.	Core; IN01	PMID 34146513
FT-0167	Gousias, Konstantinos; 2022	Distinct Behavior of Traumatic versus Nontraumatic Intracerebral Hematomas: Different Biology or Impact of Age?	Core; IN01	PMID 34126640
FT-0168	Tang, Zhiji; 2021	Outcomes of Traumatic Brain-Injured Patients With Glasgow Coma Scale < 5 and Bilateral Dilated Pupils Undergoing Decompressive Craniectomy.	Core; IN02	PMID 34113309

Report ID	Author / year	Title	Current disposition	Identifiers
FT-0169	Roberts, Ian; 2021	Tranexamic acid to reduce head injury death in people with traumatic brain injury: the CRASH-3 international RCT.	Exclude; EX02_POPULATION	PMID 33928903
FT-0170	Assamadi, M; 2021	[Epidemiological aspects and prognostic factors of severe traumatic brain injuries].	Exclude; EX02_POPULATION	PMID 33901522
FT-0171	Suner, Halil Ibrahim; 2021	Effect of decompressive hemicraniectomy in patients with acute middle cerebral artery infarction.	Core; IN01	PMID 33890450
FT-0172	Blanco, Georgina; 2021	Acute toxicity from the synthetic cathinone N-ethylpentylone (ephylone) in the United Kingdom.	Core; IN01	PMID 33855924
FT-0173	Chico-Fernandez, Mario; 2021	Factors associated with death due to trauma in patients with a Glasgow Coma Scale score of 3 and bilateral fixed dilated pupils.	Exclude; EX07	PMID 33750053
FT-0174	Adeleye, Amos Olufemi; 2021	Mannitol Dosing Error during Pre-neurosurgical Care of Head Injury: A Neurosurgical In-Hospital Survey from Ibadan, Nigeria.	Exclude; EX07	PMID 33531778
FT-0175	Paget, Louis-Marie; 2021	Trauma centre admissions for traumatic brain injury in France: One-year epidemiological analysis of prospectively collected data.	Exclude; EX07	PMID 33493628
FT-0177	Hong, Sukwoo; 2021	Is using intracerebral hemorrhage scoring systems valid for mortality prediction in surgically treated patients?	Exclude; EX02_POPULATION	PMID 33411094
FT-0178	Menozzi, Marco; 2021	The use of automated pupillometry in critically ill cirrhotic patients with hepatic encephalopathy.	Core; IN	PMID 33388562
FT-0179	Giede-Jeppe, Antje; 2021	Automated Pupillometry Identifies Absence of Intracranial Pressure Elevation in Intracerebral Hemorrhage Patients.	Exclude; EX07	PMID 33367973
FT-0180	Tian, Runfa; 2021	Prognostic Analysis of Emergency Decompressive Craniectomy for Patients with Severe Traumatic Brain Injury with Bilateral Fixed Dilated Pupils.	Exclude; EX02	PMID 33307262
FT-0181	Mahmood, Abda; 2021	Effect of tranexamic acid on intracranial haemorrhage and infarction in patients with traumatic brain injury: a pre-planned substudy in a sample of CRASH-3 trial patients.	Exclude; EX07	PMID 33262252
FT-0182	Uppar, Alok; 2021	Surgical outcomes of Supra- Infra Tentorial Extradural Hematoma (SIEDH): A retrospective study from a single institution.	Exclude; EX02_POPULATION	PMID 33243700
FT-0183	Jabbarli, Ramazan; 2020	The PRESSURE score to predict decompressive craniectomy after aneurysmal subarachnoid haemorrhage.	Exclude; EX07	PMID 33215084
FT-0184	Celi, Fernando; 2020	Decompressive Craniectomy for Traumatic Brain Injury: In-hospital Mortality-Associated Factors.	Core; IN01	PMID 33144798

Report ID	Author / year	Title	Current disposition	Identifiers
FT-0185	El Ahmadieh, Tarek Y; 2021	Automated Pupillometry as a Triage and Assessment Tool in Patients with Traumatic Brain Injury.	Exclude; EX07	PMID 33011358
FT-0186	Trevisi, Gianluca; 2020	Acute subdural hematoma in the elderly: outcome analysis in a retrospective multicentric series of 213 patients.	Core; IN01	PMID 33002873
FT-0187	Castano-Leon, Ana M; 2023	Surgery for acute subdural hematoma: the value of pre-emptive decompressive craniectomy by propensity score analysis.	Exclude; EX07	PMID 32972116
FT-0188	Ter Avest, Ewoud; 2021	Prehospital clinical signs are a poor predictor of raised intracranial pressure following traumatic brain injury.	Core; IN	PMID 32948620
FT-0190	Jouffroy, Romain; 2020	Pupil Reactivity in Refractory Out-of-Hospital Cardiac Arrest Treated by Extra-Corporeal Cardiopulmonary Resuscitation.	Exclude; EX07	PMID 32864644
FT-0191	Amir, Anum; 2020	Organophosphate Poisoning: Demographics, Severity Scores and Outcomes From National Poisoning Control Centre, Karachi.	Exclude; EX02_POPULATION	PMID 32626615
FT-0192	Moalla, Khadija Sonda; 2020	[Prognostic factors for mortality due to acute arterial stroke in a North African population].	Core; IN01	PMID 32537055
FT-0194	Achamallah, Natalie; 2020	Pupillary Light Reflex Is Not Abolished by Epinephrine and Atropine Given During Advanced Cardiac Life Support in Patients Who Achieve Return of Spontaneous Circulation.	Core; IN	PMID 32066312
FT-0196	Robba, Chiara; 2020	Tracheostomy practice and timing in traumatic brain-injured patients: a CENTER-TBI study.	Exclude; EX07	PMID 32025780
FT-0197	Kim, Ji Hee; 2020	Factors associated with the development and outcome of hydrocephalus after decompressive craniectomy for traumatic brain injury.	Exclude; EX07	PMID 31953782
FT-0199	Poppe, Michael; 2020	Extracorporeal cardiopulmonary resuscitation at the emergency department: A retrospective patient selection evaluation.	Core; IN01	PMID 31860604
FT-0200	Moon, Yeji; 2021	Neuro-Ophthalmologic Features and Outcomes of Thalamic Infarction: A Single-Institutional 10-Year Experience.	Core; IN01	PMID 31851027
FT-0201	Pavarin, Raimondo Maria; 2019	Ketamine abusers referring to emergency departments in northern Italy: a cross- sectional study.	Exclude; EX07	PMID 31850860
FT-0202	Vankipuram, Siddharth; 2020	A Comparative Analysis Between Four-Quadrant Osteoplastic Decompressive Craniotomy versus Conventional Decompressive Craniectomy for Traumatic Brain Injury.	Core; IN01	PMID 31821915
FT-0203	Bowers, Kaitlin M; 2019	Administration of Naloxone by Prehospital Personnel: A Retrospective Analysis.	Core; IN01	PMID 31700715
FT-0204	Bouvet, Perrine; 2019	Long-term Outcomes of Critically Ill Patients With Stroke Requiring Mechanical Ventilation.	Exclude; EX07	PMID 31676523

Report ID	Author / year	Title	Current disposition	Identifiers
FT-0205	Tang, Zhiji; 2020	Predictors of 30-Day Mortality in Traumatic Brain-Injured Patients after Primary Decompressive Craniectomy.	Core; IN01	PMID 31629151
FT-0206	Not parsed; 2019	Effects of tranexamic acid on death, disability, vascular occlusive events and other morbidities in patients with acute traumatic brain injury (CRASH-3): a randomised, placebo-controlled trial.	Exclude; EX02_POPULATION	PMID 31623894
FT-0207	Sabbagh, Mohammad Amr; 2020	Causes of Horner Syndrome: A Study of 318 Patients.	Exclude; EX02_POPULATION	PMID 31609831
FT-0208	Cheng, Yajun; 2020	External Validation and Modification of the EDEMA Score for Predicting Malignant Brain Edema After Acute Ischemic Stroke.	Exclude; EX03	PMID 31549349
FT-0210	Gravesteijn, B Y; 2020	Variation in the practice of tracheal intubation in Europe after traumatic brain injury: a prospective cohort study.	Exclude; EX02_POPULATION	PMID 31520421
FT-0211	Venkatasubba Rao, Chethan P; 2020	Global Survey of Outcomes of Neurocritical Care Patients: Analysis of the PRINCE Study Part 2.	Exclude; EX07	PMID 31486027
FT-0212	Llompart-Pou, Juan Antonio; 2019	Neuromonitoring in the severe traumatic brain injury. Spanish Trauma ICU Registry (RETRAUCI).	Exclude; EX07	PMID 31466814
FT-0214	Czorlich, Patrick; 2020	Operative versus non-operative treatment of traumatic brain injuries in patients 80 years of age or older.	Core; IN01	PMID 31414197
FT-0216	Matsuo, Kazuya; 2020	Machine Learning to Predict In-Hospital Morbidity and Mortality after Traumatic Brain Injury.	Exclude; EX02_POPULATION	PMID 31359814
FT-0218	Winans, Nathan J; 2020	Modeling the return to consciousness after severe traumatic brain injury at a large academic level 1 trauma center.	Core; IN01	PMID 31200372
FT-0219	Areas, Fernando Zanela; 2019	Predictors of Hospital Mortality and the Related Burden of Disease in Severe Traumatic Brain Injury: A Prospective Multicentric Study in Brazil.	Exclude; EX07	PMID 31105642
FT-0220	Baharvahdat, Humain; 2019	Malignant Cerebral Venous Infarction: Decompressive Craniectomy versus Medical Treatment.	Exclude; EX02_POPULATION	PMID 31096029
FT-0221	Riker, Richard R; 2020	Neurological Pupil Index and Pupillary Light Reflex by Pupillometry Predict Outcome Early After Cardiac Arrest.	Core; IN	PMID 31069659 DOI 10.1007/s12028-019-00717-4
FT-0222	Jahns, Fritz-Patrick; 2019	Quantitative pupillometry for the monitoring of intracranial hypertension in patients with severe traumatic brain injury.	Exclude; EX07	PMID 31046817
FT-0223	Robba, Chiara; 2020	Comparison of 2 Automated Pupillometry Devices in Critically Ill Patients.	Core; IN	PMID 31033624

Report ID	Author / year	Title	Current disposition	Identifiers
FT-0224	Osman, Mohamed; 2019	Correlation of Objective Pupillometry to Midline Shift in Acute Stroke Patients.	Exclude; EX07	PMID 31031146
FT-0225	Aslan, Fatih; 2019	Correlation of Clinical Findings With Computed Tomography in Orbital Traumas.	Exclude; EX07	PMID 31022136
FT-0226	Choffat, Caroline; 2019	Effect of Pre-Hospital Intubation in Patients with Severe Traumatic Brain Injury on Outcome: A Prospective Cohort Study.	Exclude; EX07	PMID 30959868
FT-0227	Adeleye, Amos O; 2019	Trauma demography and clinical epidemiology of motorcycle crash-related head injury in a neurosurgery practice in an African developing country.	Exclude; EX02_POPULATION	PMID 30946601
FT-0228	Roberts, Derek J; 2019	Can trauma surgeons keep up? A prospective cohort study comparing outcomes between patients with traumatic brain injury cared for in a trauma versus neuroscience intensive care unit.	Core; IN01	PMID 30899790
FT-0229	Akbik, Omar S; 2019	Mortality and Functional Outcome in Surgically Evacuated Acute Subdural Hematoma in Elderly Patients.	Core; IN01	PMID 30885866
FT-0230	Zheng, Kuang; 2019	Poor-Grade Aneurysmal Subarachnoid Hemorrhage: Risk Factors Affecting Clinical Outcomes in Intracranial Aneurysm Patients in a Multi-Center Study.	Core; IN01	PMID 30873104
FT-0231	Roth, Christian; 2019	Progress of intracranial pressure and cerebral perfusion pressure in patients during the development of brain death.	Core; IN	PMID 30731304
FT-0232	Schwake, Michael; 2020	Second-look strokectomy of cerebral infarction areas in patients with severe herniation.	Exclude; EX07	PMID 30611135
FT-0233	Lee, Hui Jai; 2019	A feasibility study for the continuous measurement of pupillary response using the pupillography during CPR in out-of-hospital cardiac arrest patients.	Core; IN	PMID 30599180 DOI 10.1016/j.resuscitation.2018.11.016
FT-0234	Svedung Wettervik, Teodor; 2019	Temporal Neurophysiological Dynamics in Traumatic Brain Injury: Role of Pressure Reactivity and Optimal Cerebral Perfusion Pressure for Predicting Outcome.	Exclude; EX07	PMID 30595128
FT-0235	Fathalla, Hussein; 2018	Managing military penetrating brain injuries in the war zone: lessons learned.	Exclude; EX02_POPULATION	PMID 30544315
FT-0236	Tunthanathip, Thara; 2018	Blast-induced traumatic brain injury: the experience from a level I trauma center in southern Thailand.	Core; IN01	PMID 30544306
FT-0237	Volpi, Paola Cristina; 2018	Trajectories of early secondary insults correlate to outcomes of traumatic brain injury: results from a large, single centre, observational study.	Exclude; EX07	PMID 30518336
FT-0238	Marshall, Matthew; 2019	Feasibility and Variability of Automated Pupillometry Among Stroke Patients and Healthy Participants: Potential Implications for Clinical Practice.	Exclude; EX07	PMID 30489422
FT-0239	Udekwu, Pascal; 2019	Predictors of Futility in Near-Hanging and Therapeutic Hypothermia.	Exclude; EX02_POPULATION	PMID 30376419

Report ID	Author / year	Title	Current disposition	Identifiers
FT-0240	Chang, Victoria A; 2019	Isolated Anisocoria as a Presenting Stroke Code Symptom is Unlikely to Result in Alteplase Administration.	Exclude; EX07	PMID 30322757
FT-0241	Ong, C; 2019	Effects of Osmotic Therapy on Pupil Reactivity: Quantification Using Pupillometry in Critically Ill Neurologic Patients.	Core; IN	PMID 30298336
FT-0242	Alali, Aziz S; 2019	A clinical decision rule to predict intracranial hypertension in severe traumatic brain injury.	Core; IN02	PMID 30265194
FT-0244	Rached, Mohamed A K B; 2019	Comparison of two simple models for prediction of short term mortality in patients after severe traumatic brain injury.	Exclude; EX07	PMID 30213562
FT-0245	Rodriguez-Fernandez, Sonia; 2018	Validation of the ICH score in patients with spontaneous intracerebral haemorrhage admitted to the intensive care unit in Southern Spain.	Exclude; EX07	PMID 30104314
FT-0246	Hoogmoed, Jantien; 2018	Early Treatment Decisions in Poor-Grade Patients with Subarachnoid Hemorrhage.	Exclude; EX07	PMID 30077026
FT-0247	Slavica, Vucinic; 2018	Acute organophosphate poisoning: 17 years of experience of the National Poison Control Center in Serbia.	Exclude; EX02_POPULATION	PMID 30055297
FT-0248	Vucinic, Slavica; 2018	Clinical and analytical experience of the National Poison Control Centre with synthetic cannabinoids.	Exclude; EX02_POPULATION	PMID 29990297
FT-0249	Nannoni, Stefania; 2018	Horner syndrome in ipsilateral lenticulostriate stroke: a novel localization for a classic stroke syndrome.	Core; IN01	PMID 29987669
FT-0251	Takizawa, Go; 2018	Relative Afferent Pupillary Defects in Homonymous Visual Field Defects Caused by Stroke of the Occipital Lobe Using Pupillometer.	Core; IN	PMID 29796045
FT-0252	Nourallah, Basil; 2018	Midline Shift is Unrelated to Subjective Pupillary Reactivity Assessment on Admission in Moderate and Severe Traumatic Brain Injury.	Exclude; EX02	PMID 29619661
FT-0253	Javaudin, F; 2018	Prognostic performance of early absence of pupillary light reaction after recovery of out of hospital cardiac arrest.	Core; IN	PMID 29545138
FT-0254	Park, Hyun-Seok; 2018	Safety and Efficacy of Hypothermia (34 degrees C) after Hemicraniectomy for Malignant MCA Infarction.	Exclude; EX07	PMID 29526071
FT-0255	Sadaka, Farid; 2018	Is it possible to recover from traumatic brain injury and a Glasgow coma scale score of 3 at emergency department presentation?	Exclude; EX07	PMID 29433914
FT-0256	Huang, Jen-Fu; 2019	Deep coma does not always predict poor outcomes among patients with polytrauma.	Core; IN01	PMID 29427061
FT-0257	Nesseler, Nicolas; 2018	Patient factors and outcomes associated with the withdrawal or withholding of life-sustaining therapies in mechanically ventilated brain-injured patients: An observational multicentre study.	Core; IN01	PMID 29419564

Report ID	Author / year	Title	Current disposition	Identifiers
FT-0258	Zijlstra, I A; 2018	Ruptured middle cerebral artery aneurysms with a concomitant intraparenchymal hematoma: the role of hematoma volume.	Exclude; EX07	PMID 29356856
FT-0261	Sumiyoshi, Kyoko; 2017	Outcome of traumatic brain injury in patients on antiplatelet agents: a retrospective 20-year observational study in a single neurosurgery unit.	Core; IN01	PMID 28991499
FT-0263	Vedantam, Aditya; 2018	Clinical characteristics and temporal profile of recovery in patients with favorable outcomes at 6 months after severe traumatic brain injury.	Exclude; EX02_POPULATION	PMID 28937323
FT-0264	Portran, Philippe; 2017	Pupillary abnormalities in non-selected critically ill patients: an observational study.	Exclude; EX07	PMID 28932559
FT-0265	Guo, Cheng; 2017	Swirl sign in traumatic acute epidural hematoma: prognostic value and surgical management.	Core; IN01	PMID 28894943
FT-0266	Viejo-Moreno, Ruben; 2017	[Emergency treatment for traumatic cardiac arrest: prognostic factors and hospital outcome].	Core; IN01	PMID 28825249
FT-0267	Chesnut, Randall M; 2018	A Method of Managing Severe Traumatic Brain Injury in the Absence of Intracranial Pressure Monitoring: The Imaging and Clinical Examination Protocol.	Exclude; EX07	PMID 28726590
FT-0268	Mao, Yan-Chiao; 2017	Bungarus multicinctus multicinctus Snakebite in Taiwan.	Core; IN01	PMID 28719273
FT-0270	Goedemans, Taco; 2017	Neurologic Outcome After Decompressive Craniectomy: Predictors of Outcome in Different Pathologic Conditions.	Core; IN02	PMID 28642178
FT-0271	Banta-Green, Caleb J; 2017	Heroin and pharmaceutical opioid overdose events: Emergency medical response characteristics.	Exclude; EX07	PMID 28623805
FT-0273	Kamran, Saadat; 2017	Decompressive Hemicraniectomy for Malignant Middle Cerebral Artery Stroke: South Asian Experience.	Exclude; EX07	PMID 28579508
FT-0274	Denninghoff, Kurt R; 2017	Prehospital Intubation is Associated with Favorable Outcomes and Lower Mortality in ProTECT III.	Exclude; EX04_NO_RELEVANT_PUPIL_DATA	PMID 28489506
FT-0276	Chieregato, Arturo; 2017	Aggressive medical management of acute traumatic subdural hematomas before emergency craniotomy in patients presenting with bilateral unreactive pupils. A cohort study.	Exclude; EX07	PMID 28435989
FT-0277	Li, Lai-Fung; 2017	Outcome after operative intervention for traumatic brain injuries in the elderly.	Core; IN01	PMID 28413530
FT-0278	Sauvigny, Thomas; 2018	Intracranial pressure in patients undergoing decompressive craniectomy: new perspective on thresholds.	Exclude; EX02	PMID 28409728
FT-0279	Mian, Asim Z; 2017	Radiological imaging features of the basal ganglia that may predict progression to hemicraniectomy in large territory middle cerebral artery infarct.	Exclude; EX02_POPULATION	PMID 28353064
FT-0280	Helander, Anders; 2017	Intoxications involving acrylfentanyl and other novel designer fentanyls - results from the Swedish STRIDA project.	Core; IN01	PMID 28349714

Report ID	Author / year	Title	Current disposition	Identifiers
FT-0281	Suehiro, Eiichi; 2017	Directions for Use of Intracranial Pressure Monitoring in the Treatment of Severe Traumatic Brain Injury Using Data from The Japan Neurotrauma Data Bank.	Exclude; EX07	PMID 28335668
FT-0282	Kesmarky, Klara; 2017	Comparison of Two Predictive Models for Short-Term Mortality in Patients after Severe Traumatic Brain Injury.	Exclude; EX07	PMID 28323524
FT-0284	Ojaghihaghighi, Seyedhossein; 2017	Comparison of neurological clinical manifestation in patients with hemorrhagic and ischemic stroke.	Core; IN	PMID 28123618
FT-0285	Isbister, Geoffrey K; 2017	Adult clonidine overdose: prolonged bradycardia and central nervous system depression, but not severe toxicity.	Exclude; EX02_POPULATION	PMID 28107093
FT-0286	Woischneck, D; 2017	MRI detection of cerebral lesions in post-traumatic anisocoria: specificity and prognostic significance.	Exclude; EX02_POPULATION	PMID 28069157
FT-0287	Friedman, Matt S; 2017	A Prospective Analysis of Patients Presenting for Medical Attention at a Large Electronic Dance Music Festival.	Exclude; EX02_POPULATION	PMID 27978861
FT-0289	Wakerley, Benjamin R; 2016	Clinical classification of 103 Japanese patients with Guillain-Barre syndrome.	Exclude; EX02_POPULATION	PMID 27653863
FT-0291	Backberg, Matilda; 2016	Adverse events related to the new psychoactive substance 3-fluorophenmetrazine - results from the Swedish STRIDA project.	Core; IN	PMID 27491700
FT-0292	Hamed, Motaz; 2016	Acute Traumatic Subdural Hematoma: Surgical Management in the Presence of Cerebral Herniation-A Single-Center Series and Multivariate Analysis.	Core; IN02	PMID 27465422
FT-0294	Kwon, Young Sub; 2016	Craniotomy or Decompressive Craniectomy for Acute Subdural Hematomas: Surgical Selection and Clinical Outcome.	Exclude; EX07	PMID 27182498
FT-0295	Dhakal, Laxmi P; 2016	Early Absent Pupillary Light Reflexes After Cardiac Arrest in Patients Treated with Therapeutic Hypothermia.	Core; IN	PMID 27135180
FT-0296	Kerr, Robert G; 2016	Underestimation of Pupil Size by Critical Care and Neurosurgical Nurses.	Core; IN	PMID 27134226
FT-0297	Waugh, Jennifer; 2016	Epidemiology and clinical features of toxicity following recreational use of synthetic cannabinoid receptor agonists: a report from the United Kingdom National Poisons Information Service.	Exclude; EX02_POPULATION	PMID 27091041
FT-0298	Friedman, Matt S; 2016	Validation of Criteria to Guide Prehospital Naloxone Administration for Drug-Related Altered Mental Status.	Core; IN	PMID 27083903
FT-0299	Couret, David; 2016	Reliability of standard pupillometry practice in neurocritical care: an observational, double-blinded study.	Core; IN	PMID 27072310

Report ID	Author / year	Title	Current disposition	Identifiers
FT-0300	Soyer, Benjamin; 2016	Outcome of a cohort of severe cerebral venous thrombosis in intensive care.	Exclude; EX07	PMID 27068929
FT-0301	Emami, Pedram; 2017	Impact of Glasgow Coma Scale score and pupil parameters on mortality rate and outcome in pediatric and adult severe traumatic brain injury: a retrospective, multicenter cohort study.	Exclude; EX02	PMID 27035177
FT-0302	Strnad, Matej; 2017	Predictors of mortality in patients with isolated severe traumatic brain injury.	Exclude; EX07	PMID 26968575
FT-0303	Charry, Jose D; 2016	Results of early cranial decompression as an initial approach for damage control therapy in severe traumatic brain injury in a hospital with limited resources.	Exclude; EX07	PMID 26933337
FT-0304	Picetti, Edoardo; 2016	Delayed Fever and Neurological Outcome after Cardiac Arrest: A Retrospective Clinical Study.	Core; IN01	PMID 26896091
FT-0305	Ryu, Jeong-Am; 2016	Spontaneous intracranial haemorrhage in critically ill patients with malignancies.	Exclude; EX07	PMID 26868952
FT-0306	Helander, Anders; 2016	Intoxications involving the fentanyl analogs acetylfentanyl, 4-methoxybutyrfentanyl and furanylfentanyl: results from the Swedish STRIDA project.	Core; IN	PMID 26850293
FT-0307	Torne, Ramon; 2016	Prognosis of patients in coma after acute subdural hematoma due to ruptured intracranial aneurysm.	Core; IN02	PMID 26755452
FT-0308	Petgrave-Perez, Alexander; 2016	[Epidemiological profile of traumatic brain injury at the Dr. Rafael A. Calderon Guardia Hospital, Neurosurgery Department, during the period from 2007 to 2012].	Exclude; EX02_POPULATION	PMID 26671008
FT-0309	Mao, Xiang; 2015	Decompressive craniectomy for severe traumatic brain injury patients with fixed dilated pupils.	Core; IN01	PMID 26543370 DOI 10.2147/tcrm.s89820
FT-0310	Jelodar, Sina; 2014	Potential Risk Factors of Death in Multiple Trauma Patients.	Core; IN01	PMID 26495375
FT-0311	Charry, Jose D; 2015	Damage control of civilian penetrating brain injuries in environments of low neuro-monitoring resources.	Core; IN01	PMID 26469861
FT-0312	Lin, Chien-Min; 2015	A Prospective Randomized Study of Brain Tissue Oxygen Pressure-Guided Management in Moderate and Severe Traumatic Brain Injury Patients.	Exclude; EX03_NO_PUPIL_RELATED_RESULTS	PMID 26413530
FT-0313	Fung, Christian; 2016	Reconsidering the logic of World Federation of Neurosurgical Societies grading in patients with severe subarachnoid hemorrhage.	Exclude; EX03	PMID 26381248
FT-0314	Miyata, Kei; 2016	Therapeutic temperature modulation in severe or moderate traumatic brain injury: a propensity score analysis of data from the Nationwide Japan Neurotrauma Data Bank.	Exclude; EX07	PMID 26381247
FT-0315	Butty, Z; 2016	Horner's syndrome in patients admitted to the intensive care unit that have undergone central venous catheterization: a prospective study.	Core; IN	PMID 26381100

Report ID	Author / year	Title	Current disposition	Identifiers
FT-0316	Hassanian-Moghaddam, Hossein; 2016	Risk Factors for Mortality and Endotracheal Intubation after Methadone Intoxication.	Core; IN01	PMID 26301535
FT-0317	Shekhar, Chandra; 2015	An epidemiological study of traumatic brain injury cases in a trauma centre of New Delhi (India).	Exclude; EX02_POPULATION	PMID 26229295
FT-0318	Firsching, Raimund; 2015	Clinical, Radiologic, and Legal Significance of "Extensor Response" in Posttraumatic Coma.	Exclude; EX02_POPULATION	PMID 26216734
FT-0319	Le Roux, Gael; 2015	Recreational phenethylamine poisonings reported to a French poison control center.	Exclude; EX02	PMID 26205314
FT-0320	Lorente, Leonardo; 2015	Total antioxidant capacity is associated with mortality of patients with severe traumatic brain injury.	Core; IN01	PMID 26205136
FT-0322	Merenda, Amedeo; 2015	Predictors of clinical failure of decompressive hemicraniectomy for malignant hemispheric infarction.	Exclude; EX07	PMID 26071890
FT-0323	Sinha, Sumit; 2015	Decompressive craniectomy in traumatic brain injury: A single-center, multivariate analysis of 1,236 patients at a tertiary care hospital in India.	Exclude; EX07	PMID 25947980
FT-0324	Mojumder, Deb Kumar; 2015	Pupil to limbus ratio: Introducing a simple objective measure using two-box method for measuring early anisocoria and progress of pupillary change in the ICU.	Exclude; EX01	PMID 25883482
FT-0327	DE Souza, R L; 2015	Side of pupillary mydriasis predicts the cognitive prognosis in patients with severe traumatic brain injury.	Exclude; EX07	PMID 25678229
FT-0328	Bruera, Sebastian; 2015	Frequency and factors associated with unexpected death in an acute palliative care unit: expect the unexpected.	Core; IN	PMID 25499421
FT-0329	Chaouali, Nadia; 2014	[Acute poisoning with anticholinesterase carbamate pesticides: methomyl-lannate(R)].	Exclude; EX07	PMID 25486668
FT-0330	Backberg, Matilda; 2015	Characteristics of analytically confirmed 3-MMC-related intoxications from the Swedish STRIDA project.	Exclude; EX02_POPULATION	PMID 25422862
FT-0331	Lin, Hong; 2016	Novel Clinical Scale for Evaluating Pre-Operative Risk of Cerebral Herniation from Traumatic Epidural Hematoma.	Core; IN02	PMID 25393339
FT-0332	Kamour, Ashraf; 2014	Patterns of presentation and clinical features of toxicity after reported use of ([2-aminopropyl]-2,3-dihydrobenzofurans), the 'benzofuran' compounds. A report from the United Kingdom National Poisons Information Service.	Exclude; EX02_POPULATION	PMID 25345418
FT-0333	Chen, Jefferson William; 2014	Infrared pupillometry, the Neurological Pupil index and unilateral pupillary dilation after traumatic brain injury: implications for treatment paradigms.	Core; IN	PMID 25332854

Report ID	Author / year	Title	Current disposition	Identifiers
FT-0334	Saade, Nelson; 2014	Evaluation of prognostic factors of decompressive craniectomy in the treatment of severe traumatic brain injury.	Core; IN01	PMID 25295986
FT-0335	Gonzalez-Robledo, J; 2015	Prognostic factors associated with mortality in patients with severe trauma: from prehospital care to the Intensive Care Unit.	Exclude; EX07	PMID 25189470
FT-0336	Epstein, Daniel S; 2015	Acute traumatic coagulopathy in the setting of isolated traumatic brain injury: Definition, incidence and outcomes.	Exclude; EX07	PMID 25153987
FT-0337	Forrester, Mathias B; 2014	NBOMe designer drug exposures reported to Texas poison centers.	Exclude; EX02_POPULATION	PMID 25115175
FT-0338	Rollins, Mark D; 2014	Pupillary effects of high-dose opioid quantified with infrared pupillometry.	Exclude; EX07	PMID 25068603
FT-0339	Gressot, Loyola V; 2014	Predictors of outcome in civilians with gunshot wounds to the head upon presentation.	Exclude; EX02_POPULATION	PMID 24995781
FT-0341	Hohl, Alexandre; 2014	Role of hormonal levels on hospital mortality for male patients with severe traumatic brain injury.	Core; IN01	PMID 24841415
FT-0344	Wang, Wen-hao; 2014	Risk factors for post-traumatic massive cerebral infarction secondary to space-occupying epidural hematoma.	Exclude; EX02_POPULATION	PMID 24773559
FT-0345	Lyrer, Philippe A; 2014	Clinical import of Horner syndrome in internal carotid and vertebral artery dissection.	Core; IN01	PMID 24727317
FT-0346	Bao, Long; 2014	Fever burden is an independent predictor for prognosis of traumatic brain injury.	Core; IN01	PMID 24626046
FT-0348	Kim, Deok-Ryeong; 2014	Significance of intracranial pressure monitoring after early decompressive craniectomy in patients with severe traumatic brain injury.	Core; IN01	PMID 24570814
FT-0349	Aarabi, Bizhan; 2014	Predictors of outcome in civilian gunshot wounds to the head.	Exclude; EX02_POPULATION	PMID 24506239
FT-0350	Sharma, Bhavana; 2014	Ocular manifestations of head injury and incidence of post-traumatic ocular motor nerve involvement in cases of head injury: a clinical review.	Exclude; EX02_POPULATION	PMID 24469117
FT-0351	Wolf, Harald; 2014	Risk factors indicating the need for cranial CT scans in elderly patients with head trauma: an Austrian trial and comparison with the Canadian CT Head Rule.	Exclude; EX07	PMID 24313609
FT-0352	Kim, Kang Rae; 2013	Clinical Comparison of 30-Day Mortalities and 6-Month Functional Recoveries after Spontaneous Intracerebral Hemorrhage in Patients with or without End-Stage Renal Disease.	Core; IN01	PMID 24278643
FT-0353	Femi, Owolabi Lukman; 2013	Factors associated with death and predictors of one-month mortality from stroke in Kano, Northwestern Nigeria.	Core; IN01	PMID 24174802

Report ID	Author / year	Title	Current disposition	Identifiers
FT-0354	Vinas-Rios, Juan Manuel; 2014	Hypocalcaemia as a prognostic factor of early mortality in moderate and severe traumatic brain injury.	Exclude; EX02	PMID 24139087
FT-0355	Owolabi, Lukman Femi; 2013	Factors associated with death and predictors of 1-month mortality in nontraumatic coma in a tertiary hospital in Northwestern Nigeria.	Exclude; EX07	PMID 24133329
FT-0356	Matsukawa, Hidetoshi; 2013	Acute alcohol intoxication, diffuse axonal injury and intraventricular bleeding in patients with isolated blunt traumatic brain injury.	Exclude; EX07	PMID 24102331
FT-0358	Suehiro E; 2014	Survey of brain temperature management in patients with traumatic brain injury in the Japan neurotrauma data bank.	Exclude; EX07	PMID 24047191 DOI 10.1089/neu.2013.3057
FT-0359	Dunham, C Michael; 2013	In emergently ventilated trauma patients, low end-tidal CO2 and low cardiac output are associated and correlate with hemodynamic instability, hemorrhage, abnormal pupils, and death.	Exclude; EX07	PMID 24020798
FT-0361	Arslan, Alp; 2012	[Infection in patients with isolated head injury: risk factors and the impact on treatment cost].	Exclude; EX07	PMID 23588909
FT-0362	Fatigba, Holden O; 2013	The exploratory burr hole: indication and results at one departmental hospital of benin.	Exclude; EX02_POPULATION	PMID 23577270
FT-0363	Munivenkatappa, Ashok; 2013	Domestic animal-related neuro-trauma: An account, from a tertiary institute.	Exclude; EX02_POPULATION	PMID 23546342
FT-0364	Yuan, Q; 2013	Comparative study of decompressive craniectomy in traumatic brain injury with or without mass lesion.	Exclude; EX02_POPULATION	PMID 23384251
FT-0365	Cheng, K L; 2013	Chinese herbal medicine-induced anticholinergic poisoning in Hong Kong.	Exclude; EX02_POPULATION	PMID 23378353
FT-0366	Ziai, Wendy C; 2013	Cerebral herniation associated with central venous catheter insertion: risk assessment.	Core; IN	PMID 23159141
FT-0367	Dunham, C Michael; 2012	Emergency department noninvasive (NICOM) cardiac outputs are associated with trauma activation, patient injury severity and host conditions and mortality.	Exclude; EX07	PMID 23019674
FT-0368	Frutos Bernal, E; 2013	[Prognostic factors in severe traumatic brain injury].	Exclude; EX02_POPULATION	PMID 22854619
FT-0369	Akyel, Serkan; 2012	[Effective factors in the outcome of head injury].	Exclude; EX02_POPULATION	PMID 22792818
FT-0370	Xiao, Bo; 2012	A randomized study of urgent computed tomography-based hematoma puncture and aspiration in the emergency department and subsequent evacuation using craniectomy versus craniectomy only.	Core; IN	PMID 22769066

Report ID	Author / year	Title	Current disposition	Identifiers
FT-0371	Tao, Wen-Dan; 2012	Posterior versus anterior circulation infarction: how different are the neurological deficits?	Exclude; EX07	PMID 22678088
FT-0372	Hoffmann, M; 2012	Pupil evaluation in addition to Glasgow Coma Scale components in prediction of traumatic brain injury and mortality.	Exclude; EX07	PMID 22441866
FT-0373	Kirkman, Matthew A; 2012	Imaging in young adults with intracerebral hemorrhage.	Core; IN01	PMID 22525367
FT-0374	Kim, Kyu Hong; 2013	Comparisons of 30-day mortalities and 90-day functional recoveries after first and recurrent primary intracerebral hemorrhage attacks: a multiple-institute retrospective study.	Core; IN01	PMID 22484068
FT-0375	Banerjee, Indranil; 2012	Clinico-epidemiological characteristics of patients presenting with organophosphorus poisoning.	Exclude; EX07	PMID 22454830
FT-0376	Yanagawa, Youichi; 2012	Results of single burr hole drainage for acute subdural hematoma with non-reactive pupil.	Exclude; EX07	PMID 22437294
FT-0378	Song, Zhi; 2012	Prediction of coma and anisocoria based on computerized tomography findings in patients with supratentorial intracerebral hemorrhage.	Exclude; EX07	PMID 22257515
FT-0379	Sharma, Ranjana; 2012	Methanol poisoning: ocular and neurological manifestations.	Core; IN	PMID 22127151
FT-0380	Taussky, Philipp; 2012	Age and salvageability: analysis of outcome of patients older than 65 years undergoing craniotomy for acute traumatic subdural hematoma.	Core; IN01	PMID 22120569
FT-0381	Matsuda, Kimiko; 2011	Assessment of the severity of organophosphate (fenitrothion) poisoning based on its serum concentration and clinical parameters.	Core; IN01	PMID 22077246
FT-0384	Schneider, Eric B; 2011	Premorbid statin use is associated with improved survival and functional outcomes in older head-injured individuals.	Exclude; EX04_NO_PUPIL_RELATED_RESULTS	PMID 21986733
FT-0385	Spiller, Henry A; 2011	Clinical experience with and analytical confirmation of "bath salts" and "legal highs" (synthetic cathinones) in the United States.	Exclude; EX02_POPULATION	PMID 21824061
FT-0386	Shamim, Muhammad Shahzad; 2011	Emergency department predictors of tracheostomy in patients with isolated traumatic brain injury requiring emergency cranial decompression.	Exclude; EX02_POPULATION	PMID 21819192
FT-0387	Hohl, Alexandre; 2012	Plasma levels of oxidative stress biomarkers and hospital mortality in severe head injury: a multivariate analysis.	Core; IN01	PMID 21803537
FT-0388	Matsukawa, Hidetoshi; 2011	Genu of corpus callosum as a prognostic factor in diffuse axonal injury.	Exclude; EX07	PMID 21780860
FT-0390	Nathoo, Narendra; 2011	Brain abscess: management and outcome analysis of a computed tomography era experience with 973 patients.	Exclude; EX02_POPULATION	PMID 21704942
FT-0391	Kiboi, Julius G; 2011	Predictors of functional recovery in African patients with traumatic intracranial hematomas.	Exclude; EX02_POPULATION	PMID 21704911

Report ID	Author / year	Title	Current disposition	Identifiers
FT-0392	Edberg, M; 2011	Neurointensive care of patients with severe community-acquired meningitis.	Core; IN02	PMID 21615347
FT-0394	Wagner, Ingrid; 2011	Effects of continuous hypertonic saline infusion on perihemorrhagic edema evolution.	Exclude; EX03	PMID 21512173
FT-0396	Hauer, Eva-Maria; 2011	Early continuous hypertonic saline infusion in patients with severe cerebrovascular disease.	Exclude; EX03	PMID 21494103
FT-0398	Tazarourte, K; 2011	Pre-hospital transcranial Doppler in severe traumatic brain injury: a pilot study.	Core; IN01	PMID 21288224
FT-0399	Rahme, Ralph; 2011	Outcome of severe arteriovenous malformation-related intracranial hemorrhage: the importance of cisternal subarachnoid hemorrhage and early seizures.	Exclude; EX02	PMID 21234617
FT-0400	Jang, Ji Hwan; 2011	Predictors of 30-day mortality and 90-day functional recovery after primary pontine hemorrhage.	Core; IN01	PMID 21218037
FT-0401	Jamous, M; 2010	Emergency decompressive craniectomy for trauma patients with Glasgow Coma Scale of 3 and bilateral fixed dilated pupils.	Core; IN02	PMID 26816228
FT-0403	Lath, Rahul; 2010	Decompressive surgery for severe cerebral venous sinus thrombosis.	Core; IN01	PMID 20644266
FT-0404	Yanagawa, Youichi; 2010	Relationship between abnormal pupillary reactivity and the outcome of a psychotropic drug overdose.	Exclude; EX07	PMID 20637386
FT-0406	Huber-Wagner, S; 2010	The sequential trauma score - a new instrument for the sequential mortality prediction in major trauma.	Exclude; EX07	PMID 20562057
FT-0407	Karasu, Aykut; 2010	Analyses of clinical prognostic factors in operated traumatic acute subdural hematomas.	Exclude; EX02_POPULATION	PMID 20517749
FT-0408	Jiang, W-J; 2010	Wingspan experience at Beijing Tiantan Hospital: new insights into the mechanisms of procedural complication from viewing intraoperative transient ischemic attacks during awake stenting for vertebrobasilar stenosis.	Exclude; EX07	PMID 21990587
FT-0409	Marina-Martinez, L; 2010	[<<RETRATO>> (REgistro de TRAuma grave de la provincia de TOledo): general view and mortality].	Exclude; EX02_POPULATION	PMID 20381200
FT-0410	Bhau, Kulwant Singh; 2010	Traumatic extradural hematoma - role of non-surgical management and reasons for conversion.	Exclude; EX07	PMID 23133222
FT-0411	Chan, Agnes N; 2010	A comparison of venlafaxine and SSRIs in deliberate self-poisoning.	Exclude; EX07	PMID 20237971
FT-0412	Theaudin, Marie; 2010	Should decompressive surgery be performed in malignant cerebral venous thrombosis?: a series of 12 patients.	Core; IN01	PMID 20185780
FT-0413	Witoonpanich, Rawiphan; 2010	Survival analysis for respiratory failure in patients with food-borne botulism.	Exclude; EX02_POPULATION	PMID 20184431

Report ID	Author / year	Title	Current disposition	Identifiers
FT-0414	Carstairs, Shaun D; 2010	Peyote and mescaline exposures: a 12-year review of a statewide poison center database.	Core; IN02	PMID 20170392
FT-0415	Huber-Wagner, S; 2009	Duration and predictors of emergency surgical operations--basis for medical management of mass casualty incidents.	Exclude; EX07	PMID 20149987
FT-0416	Gupta, Arvind; 2010	Comparison of the clinical presentation and visual outcome in open globe injuries in adults and children over 30 months.	Exclude; EX02	PMID 20037911
FT-0418	Shadnia, Shahin; 2009	Prognostic value of long QT interval in acute and severe organophosphate poisoning.	Exclude; EX02_POPULATION	PMID 19876851
FT-0421	Vallersnes, Odd Martin; 2009	Epidemic of poisoning caused by scopolamine disguised as Rohypnol tablets.	Core; IN01	PMID 19821638
FT-0424	Leong, Lim Beng; 2010	Identifying risk factors for an abnormal computed tomographic scan of the head among patients with altered mental status in the Emergency Department.	Core; IN01	PMID 19773662
FT-0425	Nelson, David W; 2010	Extended analysis of early computed tomography scans of traumatic brain injured patients and relations to outcome.	Exclude; EX07	PMID 19698072
FT-0426	Poca, Maria Antonia; 2010	Monitoring intracranial pressure in patients with malignant middle cerebral artery infarction: is it useful?	Core; IN	PMID 19663552
FT-0427	Kim, Kyu-Hong; 2009	Predictors of 30-day mortality and 90-day functional recovery after primary intracerebral hemorrhage : hospital based multivariate analysis in 585 patients.	Core; IN01	PMID 19609417
FT-0428	Martins, Evandro Tostes; 2009	Mortality in severe traumatic brain injury: a multivariated analysis of 748 Brazilian patients from Florianopolis City.	Exclude; EX07	PMID 19590314
FT-0430	Wisniewski, Marek; 2009	Acute topiramate overdose--clinical manifestations.	Core; IN02	PMID 19514879
FT-0431	Liliang, Po-Chou; 2010	Tau proteins in serum predict outcome after severe traumatic brain injury.	Core; IN01	PMID 19345376
FT-0432	Chamoun, Roukoz B; 2009	Outcome in patients with blunt head trauma and a Glasgow Coma Scale score of 3 at presentation.	Exclude; EX02_POPULATION	PMID 19326973
FT-0434	Mauritz, Walter; 2009	Outcome of brain trauma patients who have a Glasgow Coma Scale score of 3 and bilateral fixed and dilated pupils in the field.	Exclude; EX07	PMID 19282759
FT-0435	Joosse, P; 2009	Outcome and prognostic factors of traumatic brain injury: a prospective evaluation in a Jakarta University hospital.	Exclude; EX07	PMID 19269829
FT-0437	Chaudhuri, Krishanu; 2009	Survival of trauma patients with coma and bilateral fixed dilated pupils.	Core; IN02	PMID 19070839
FT-0438	Kalita J; 2009	Brain herniations in patients with intracerebral hemorrhage.	Core; IN01	PMID 19053952 DOI 10.1111/j.1600-0404.2008.01095.x

Report ID	Author / year	Title	Current disposition	Identifiers
FT-0439	Vik, Anne; 2008	Relationship of "dose" of intracranial hypertension to outcome in severe traumatic brain injury.	Exclude; EX02_POPULATION	PMID 18826355
FT-0440	Moen, Kent Goran; 2008	Direct transport versus interhospital transfer of patients with severe head injury in Norway.	Exclude; EX02_POPULATION	PMID 18784502
FT-0441	Faleiro, Rodrigo Moreira; 2008	Decompressive craniotomy: prognostic factors and complications in 89 patients.	Exclude; EX07	PMID 18641874
FT-0444	Exner, Christopher J; 2009	Organophosphate and carbamate intoxication in La Paz, Bolivia.	Exclude; EX02_POPULATION	PMID 18439788
FT-0445	Bouhours, G; 2008	[Audit of the prehospital management of severe head injured patients in the "Pays-de-la-Loire" region].	Exclude; EX07	PMID 18423956
FT-0446	Paradot, G; 2008	[Craniocerebral gunshot wounds: a study of outcome predictors].	Core; IN02	PMID 18339406
FT-0447	Quigley, Matthew R; 2008	Defining survivorship after high-grade aneurysmal subarachnoid hemorrhage.	Exclude; EX07	PMID 18221774
FT-0448	Chen, Wei-Hung; 2007	Outcome of large hemispheric infarcts: an experience of 50 patients in Taiwan.	Core; IN01	PMID 17963932
FT-0451	Malhotra, Ajai K; 2007	Computed tomographic angiography for the diagnosis of blunt carotid/vertebral artery injury: a note of caution.	Exclude; EX02_POPULATION	PMID 17893500
FT-0452	Misra, U K; 2007	Effect of single mannitol bolus in intracerebral hemorrhage.	Core; IN	PMID 17727664
FT-0453	Yurumez, Yusuf; 2007	Acute organophosphate poisoning in university hospital emergency room patients.	Exclude; EX07	PMID 17603234
FT-0455	Morgan, Mary; 2007	Olanzapine overdose: a series of analytically confirmed cases.	Exclude; EX07	PMID 17414746
FT-0456	Schultz, Kara L; 2007	Diagnostic yield of the evaluation of isolated third nerve palsy in adults.	Core; IN01	PMID 17361251
FT-0459	Kupersmith, Mark J; 2006	Magnetic resonance angiography and clinical evaluation of third nerve palsies and posterior communicating artery aneurysms.	Core; IN	PMID 17219827
FT-0460	Korfias, Stefanos; 2007	Serum S-100B protein monitoring in patients with severe traumatic brain injury.	Exclude; EX02_POPULATION	PMID 17143637
FT-0461	Cheung, Phoebe S Y; 2007	Outcome of traumatic extradural haematoma in Hong Kong.	Exclude; EX02	PMID 17097656
FT-0463	Degos, Vincent; 2006	Computed tomography-estimated specific gravity of noncontused brain areas as a marker of severity in human traumatic brain injury.	Exclude; EX07	PMID 17056960
FT-0465	Ohwaki, Kazuhiro; 2006	Surgery for patients with severe supratentorial intracerebral hemorrhage.	Core; IN01	PMID 16960289

Report ID	Author / year	Title	Current disposition	Identifiers
FT-0466	Tripathi, Mukesh; 2006	A mixture of organophosphate and pyrethroid intoxication requiring intensive care unit admission: a diagnostic dilemma and therapeutic approach.	Qualitative / linkage only; outside core	PMID 16861425
FT-0467	Oh, Hyun Soo; 2006	Comparisons of the prognostic predictors of traumatic brain injury according to admission Glasgow Coma Scale scores-based on 1- and 6-month assessments.	Exclude; EX02_POPULATION	PMID 16825846
FT-0468	Boto, G R; 2006	Severe head injury and the risk of early death.	Exclude; EX07	PMID 16740580
FT-0469	Aarabi, Bizhan; 2006	Outcome following decompressive craniectomy for malignant swelling due to severe head injury.	Exclude; EX07	PMID 16619648
FT-0470	Tien, Homer C; 2006	Do trauma patients with a Glasgow Coma Scale score of 3 and bilateral fixed and dilated pupils have any chance of survival?	Exclude; EX07	PMID 16508482
FT-0471	Avsarogullari, Levent; 2006	Acute amitraz poisoning in adults: clinical features, laboratory findings, and management.	Exclude; EX02_POPULATION	PMID 16496489
FT-0474	Balci, Kemal; 2005	Clinical and neuroradiological predictors of mortality in patients with primary pontine hemorrhage.	Core; IN01	PMID 16311143
FT-0477	Murthy, J M K; 2005	Decompressive craniectomy with clot evacuation in large hemispheric hypertensive intracerebral hemorrhage.	Core; IN01	PMID 16159072
FT-0478	King, Joseph T Jr; 2005	Early Glasgow Outcome Scale scores predict long-term functional outcome in patients with severe traumatic brain injury.	Exclude; EX02_POPULATION	PMID 16156710
FT-0479	Komotar, Ricardo J; 2005	Herniation secondary to critical postcraniotomy cerebrospinal fluid hypovolemia.	Core; IN01	PMID 16094157
FT-0480	Shah, S D; 2005	Prognostic predictors of thalamic hemorrhage.	Core; IN01	PMID 15936200
FT-0481	Misra, U K; 2005	Mannitol in intracerebral hemorrhage: a randomized controlled study.	Core; IN	PMID 15936036
FT-0482	Guloglu, Cahfer; 2005	Acute poisoning cases admitted to a university hospital emergency department in Diyarbakir, Turkey.	Exclude; EX07	PMID 15850278
FT-0483	Bahloul, Mabrouk; 2005	Neurological complications secondary to severe scorpion envenomation.	Exclude; EX02_POPULATION	PMID 15795701
FT-0484	de Bray, Jean-Michel; 2005	Isolated Horner's syndrome may herald stroke.	Exclude; EX07	PMID 15761221
FT-0485	Kilincer, C; 2005	Factors affecting the outcome of decompressive craniectomy for large hemispheric infarctions: a prospective cohort study.	Core; IN01	PMID 15739038
FT-0486	Gonul, Engin; 2005	Penetrating orbitocranial gunshot injuries.	Exclude; EX02_POPULATION	PMID 15639513

Report ID	Author / year	Title	Current disposition	Identifiers
FT-0487	Alexander, John L; 2004	Suspected opioid-related emergency medical services encounters in a rural state, 1997-2002.	Exclude; EX07	PMID 15626007
FT-0489	Anoro, Manel; 2004	[Factors related to experiencing respiratory failure in cases of opiate overdose for which care was provided in an open setting. Barcelona, Spain].	Core; IN02	PMID 15535008
FT-0490	Stocchetti, Nino; 2004	Inaccurate early assessment of neurological severity in head injury.	Exclude; EX02_POPULATION	PMID 15453984
FT-0493	Voetsch, Barbara; 2004	Basilar artery occlusive disease in the New England Medical Center Posterior Circulation Registry.	Core; IN02	PMID 15096396
FT-0494	Kinoshita, Kosaku; 2004	Risk factors for intraoperative hypotension in traumatic intracranial hematoma.	Core; IN01	PMID 15036732
FT-0495	Cruz, Julio; 2004	Successful use of the new high-dose mannitol treatment in patients with Glasgow Coma Scale scores of 3 and bilateral abnormal pupillary widening: a randomized trial.	Exclude; EX07	PMID 15035271
FT-0496	Rouxel, J-P M; 2004	[Medical prehospital rescue in head injury].	Core; IN01	PMID 14980318
FT-0497	Patel, Raj J; 2004	Prevalence of autonomic signs and symptoms in antimuscarinic drug poisonings.	Exclude; EX07	PMID 14751484
FT-0498	Van Roost, Dirk; 2003	Pseudohypoxic brain swelling: a newly defined complication after uneventful brain surgery, probably related to suction drainage.	Qualitative / linkage only; outside core	PMID 14633298
FT-0499	Glenn, Thomas C; 2003	Energy dysfunction as a predictor of outcome after moderate or severe head injury: indices of oxygen, glucose, and lactate metabolism.	Exclude; EX02_POPULATION	PMID 14526234
FT-0500	Temkin, Nancy R; 2003	Risk factors for posttraumatic seizures in adults.	Exclude; EX07	PMID 14511390
FT-0501	Lieberman, Jayme D; 2003	Use of admission Glasgow Coma Score, pupil size, and pupil reactivity to determine outcome for trauma patients.	Exclude; EX02_POPULATION	PMID 14501883
FT-0502	Rossetti, Andrea O; 2003	Central Horner's syndrome with contralateral ataxic hemiparesis: a diencephalic alternate syndrome.	Core; IN	PMID 12913193
FT-0503	Martins, Roberto S; 2003	Prognostic factors and treatment of penetrating gunshot wounds to the head.	Exclude; EX02_POPULATION	PMID 12900108
FT-0504	Strege, Rainer J; 2003	Cerebral edema leading to decompressive craniectomy: an assessment of the preceding clinical and neuromonitoring trends.	Core; IN02	PMID 12866200
FT-0505	Misra, U K; 2003	Predictors of gastrointestinal bleeding in acute intracerebral haemorrhage.	Core; IN01	PMID 12639721
FT-0507	Tokuda, Y; 2003	Pupillary evaluation for differential diagnosis of coma.	Exclude; EX02_POPULATION	PMID 12566553

Report ID	Author / year	Title	Current disposition	Identifiers
FT-0508	Fanshawe, M; 2002	Outcome of stroke patients admitted to intensive care: experience from an Australian teaching hospital.	Core; IN01	PMID 12413265
FT-0509	Singhal, A; 2002	Association between cerebrospinal fluid interleukin-6 concentrations and outcome after severe human traumatic brain injury.	Exclude; EX02_POPULATION	PMID 12225653
FT-0510	Balikova, Marie; 2002	Collective poisoning with hallucinogenous herbal tea.	Qualitative / linkage only; outside core	PMID 12208022
FT-0511	Cruz, Julio; 2002	Major clinical and physiological benefits of early high doses of mannitol for intraparenchymal temporal lobe hemorrhages with abnormal pupillary widening: a randomized trial.	Exclude; EX07	PMID 12188940
FT-0512	Keller, E; 2002	Intensive care management of patients with severe intracerebral haemorrhage after endovascular treatment of brain arteriovenous malformations.	Core; IN01	PMID 12070726
FT-0514	Grande, P-O; 2002	Treatment of intracranial hypertension and aspects on lumbar dural puncture in severe bacterial meningitis.	Core; IN	PMID 11939916
FT-0515	Matsumae, M; 2001	Pupillary abnormality on admission and brain bulging during surgery as unfavourable predictors in patients treated with hypothermia: a retrospective review of 81 patients with severe head injury.	Exclude; EX02_POPULATION	PMID 11810387
FT-0516	Dubinsky, Isser; 2002	Can specific patient variables be used to predict outcome of intracranial hemorrhage?	Core; IN	PMID 11781908
FT-0517	Schwarz, Stefan; 2002	Effects of hypertonic (10%) saline in patients with raised intracranial pressure after stroke.	Exclude; EX07	PMID 11779902
FT-0518	Banerji, S; 2001	Abuse of Coricidin HBP cough & cold tablets: episodes recorded by a poison center.	Exclude; EX02_POPULATION	PMID 11596695
FT-0519	Wijdicks, E F; 2001	MR imaging in comatose survivors of cardiac resuscitation.	Core; IN02	PMID 11559506
FT-0520	Gotoh, F; 2001	Development of a novel, weighted, quantifiable stroke scale: Japan stroke scale.	Core; IN02	PMID 11486108
FT-0521	Ono, J; 2001	Outcome prediction in severe head injury: analyses of clinical prognostic factors.	Exclude; EX02_POPULATION	PMID 11484659
FT-0522	Clusmann, H; 2001	Fixed and dilated pupils after trauma, stroke, and previous intracranial surgery: management and outcome.	Core; IN	PMID 11459888 DOI 10.1136/jnnp.71.2.175
FT-0523	Ulukaya, S; 2001	Acute amitraz intoxication in human.	Qualitative / linkage only; outside core	PMID 11430553
FT-0524	Munch, E C; 2001	Therapy of malignant intracranial hypertension by controlled lumbar cerebrospinal fluid drainage.	Exclude; EX02_POPULATION	PMID 11378607

Report ID	Author / year	Title	Current disposition	Identifiers
FT-0525	Aureli, P; 2000	An outbreak in Italy of botulism associated with a dessert made with mascarpone cream cheese.	Core; IN02	PMID 11338122
FT-0528	Ellis, A J; 2000	Subclinical seizure activity and prophylactic phenytoin infusion in acute liver failure: a controlled clinical trial.	Core; IN02	PMID 10960446
FT-0529	Servadei, F; 2000	CT prognostic factors in acute subdural haematomas: the value of the 'worst' CT scan.	Exclude; EX07	PMID 10889882
FT-0530	Fandino, J; 2000	Cerebral oxygenation and systemic trauma related factors determining neurological outcome after brain injury.	Exclude; EX02_POPULATION	PMID 10833621
FT-0532	Lubillo, S; 1999	Prognostic value of early computerized tomography scanning following craniotomy for traumatic hematoma.	Exclude; EX02_POPULATION	PMID 10507378
FT-0534	Ritter, A M; 1999	Brain stem blood flow, pupillary response, and outcome in patients with severe head injuries.	Exclude; EX02_POPULATION	PMID 10232526
FT-0535	Aibiki, M; 1999	Effect of moderate hypothermia on systemic and internal jugular plasma IL-6 levels after traumatic brain injury in humans.	Core; IN02	PMID 10195470
FT-0536	Andrefsky, J C; 1999	The ciliospinal reflex in pentobarbital coma.	Core; IN	PMID 10193607
FT-0537	St Louis, E K; 1998	Predicting neurologic deterioration in patients with cerebellar hematomas.	Core; IN01	PMID 9818861
FT-0538	Ricolfi, F; 1998	Rupture during treatment of recently ruptured aneurysms with Guglielmi electrodetachable coils.	Core; IN01	PMID 9802487
FT-0540	Schwarz, S; 1998	Effects of hypertonic saline hydroxyethyl starch solution and mannitol in patients with increased intracranial pressure after stroke.	Core; IN01	PMID 9707191
FT-0541	Wijdicks, E F; 1998	Stroke in the medical intensive-care unit.	Core; IN01	PMID 9663192
FT-0543	Kampfl, A; 1998	Prediction of recovery from post-traumatic vegetative state with cerebral magnetic-resonance imaging.	Exclude; EX07	PMID 9635948
FT-0544	Sakai, K; 1998	Outcome after external decompression for massive cerebral infarction.	Core; IN01	PMID 9597855
FT-0545	Brown, J I; 1998	Clinical significance of CSF glutamate concentrations following severe traumatic brain injury in humans.	Exclude; EX07	PMID 9555971
FT-0546	Takano, K; 1998	Differences in clinical features and computed tomographic findings between embolic and non-embolic acute ischemic stroke: a quantitative differential diagnosis.	Exclude; EX07	PMID 9550593
FT-0547	Yang, C C; 1998	Clinical experience in acute overdosage of diphenidol.	Exclude; EX04_NO_RELEVANT_PUPIL_DATA	PMID 9541039

Report ID	Author / year	Title	Current disposition	Identifiers
FT-0549	Nozaki, H; 1997	Relationship between pupil size and acetylcholinesterase activity in patients exposed to sarin vapor.	Exclude; EX02_POPULATION	PMID 9347376
FT-0550	Kelly, D F; 1997	Cerebral blood flow as a predictor of outcome following traumatic brain injury.	Exclude; EX02_POPULATION	PMID 9120627
FT-0553	Koc, R K; 1997	Acute subdural hematoma: outcome and outcome prediction.	Exclude; EX02_POPULATION	PMID 9457718
FT-0554	Quigley, M R; 1997	Defining the limits of survivorship after very severe head injury.	Core; IN02	PMID 9003251
FT-0555	Combes, P; 1996	Severe head injuries: an outcome prediction and survival analysis.	Exclude; EX07	PMID 8986491
FT-0558	Ellenberg, J H; 1996	Posttraumatic Amnesia as a predictor of outcome after severe closed head injury. Prospective assessment.	Exclude; EX07	PMID 8759985
FT-0559	Sporer, K A; 1996	Out-of-hospital treatment of opioid overdoses in an urban setting.	Core; IN02	PMID 8816181
FT-0560	Cohen, J E; 1996	Prognosis and clinical relevance of anisocoria-craniotomy latency for epidural hematoma in comatose patients.	Core; IN	PMID 8676403
FT-0561	Misra, U K; 1996	Transient renal impairment in acute intracerebral haemorrhage.	Exclude; EX07	PMID 8741083
FT-0563	Nozaki, H; 1995	Secondary exposure of medical staff to sarin vapor in the emergency room.	Core; IN01	PMID 8750130
FT-0564	Blake, P Y; 1995	MR of oculomotor nerve palsy.	Exclude; EX07	PMID 7502972
FT-0566	Krieger, D; 1995	Serial somatosensory and brainstem auditory evoked potentials in monitoring of acute supratentorial mass lesions.	Exclude; EX02_POPULATION	PMID 7774226
FT-0567	Sakas, D E; 1995	One-year outcome following craniotomy for traumatic hematoma in patients with fixed dilated pupils.	Core; IN	PMID 7760198
FT-0568	Mori, Sakan; 1995	Impact of Thalamic Hematoma on Six-Month Mortality and Motor and Cognitive Functional Outcome	Core; IN01	PMID 7709409 DOI 10.1161/01.STR.26.4.620
FT-0569	Mase, G; 1995	Immediate prognosis of primary intracerebral hemorrhage using an easy model for the prediction of survival.	Core; IN01	PMID 7625160
FT-0579	Schynoll, W; 1993	A prospective study to identify high-yield criteria associated with acute intracranial computed tomography findings in head-injured patients.	Exclude; EX02_POPULATION	PMID 8216509
FT-0580	Garnier, R; 1993	Acute fluvoxamine poisoning.	Exclude; EX02_POPULATION	PMID 7906658
FT-0584	De Mendonca, A; 1992	[Comas in the emergency room of a central hospital].	Exclude; EX07	PMID 1442192

Report ID	Author / year	Title	Current disposition	Identifiers
FT-0586	Steinke, W; 1992	Thalamic stroke. Presentation and prognosis of infarcts and hemorrhages.	Exclude; EX07	PMID 1497496
FT-0587	Lancman, M; 1992	Tegmental pontine hemorrhages: clinical features and prognostic factors.	Qualitative / linkage only; outside core	PMID 1623452 DOI 10.1017/s0317167100042323
FT-0590	Lee, J P; 1991	Acute post-traumatic intraventricular hemorrhage analysis of 25 patients with emphasis on final outcome.	Core; IN02	PMID 1950456
FT-0594	Steiger, H J; 1991	Outcome of acute supratentorial cerebral infarction in patients under 60. Development of a prognostic grading system.	Core; IN01	PMID 1950691
FT-0596	Levin, H S; 1990	Neurobehavioral outcome 1 year after severe head injury. Experience of the Traumatic Coma Data Bank.	Exclude; EX07	PMID 2213159
FT-0597	Martinez-Chuecos, J; 1990	Management of methomyl poisoning.	Exclude; EX02	PMID 2390322
FT-0598	Keane, J R; 1990	The pretectal syndrome: 206 patients.	Exclude; EX02_POPULATION	PMID 2320246
FT-0599	Katz, B; 1990	Anterior ischaemic optic neuropathy and intraocular pressure.	Exclude; EX03	PMID 2310734
FT-0600	Fraser, C L; 1990	Fatal central diabetes mellitus and insipidus resulting from untreated hyponatremia: a new syndrome.	Qualitative / linkage only; outside core	PMID 2294815
FT-0601	Walpoth, B H; 1990	Accidental deep hypothermia with cardiopulmonary arrest: extracorporeal blood rewarming in 11 patients.	Exclude; EX02	PMID 2397132
FT-0603	Foroglou, G; 1989	The timing of CT.	Exclude; EX02_POPULATION	PMID 2812367
FT-0605	Nagaseki, Y; 1989	Primary internal ophthalmoplegia due to head injury.	Core; IN01	PMID 2718803
FT-0606	Andrews, B T; 1988	The effect of intracerebral hematoma location on the risk of brain-stem compression and on clinical outcome.	Exclude; EX02_POPULATION	PMID 3418383
FT-0608	Tapaninaho, A; 1988	Emergency treatment of cerebral aneurysms with large haematomas.	Core; IN01	PMID 3394543
FT-0609	Sarabia, R; 1988	Cerebral hemisphere swelling in severe head injury patients.	Unresolved source; author-directed closure	PMID 3189019
FT-0610	Andrews, B T; 1987	Implications of systemic hypotension for the neurological examination in patients with severe head injury.	Exclude; EX02_POPULATION	PMID 3686321
FT-0611	Portenoy, R K; 1987	Intracerebral haemorrhage: a model for the prediction of outcome.	Exclude; EX07	PMID 3655832
FT-0612	Yaqub, B A; 1987	Neurologic manifestations of heatstroke at the Mecca pilgrimage.	Core; IN01	PMID 3587616

Report ID	Author / year	Title	Current disposition	Identifiers
FT-0614	Lipton, R B; 1987	Lobar vs thalamic and basal ganglion hemorrhage: clinical and radiographic features.	Exclude; EX07	PMID 3559644
FT-0617	Dan, N G; 1986	Experience with extradural haematomas in New South Wales.	Exclude; EX02_POPULATION	PMID 3461775
FT-0619	van Gijn, J; 1985	Acute hydrocephalus after aneurysmal subarachnoid hemorrhage.	Core; IN02	PMID 4020461
FT-0620	Lui, T N; 1985	Surgical treatment of spontaneous cerebellar hemorrhage.	Core; IN01	PMID 3992454
FT-0625	Turazzi, S; 1984	Review of 1,000 consecutive cases of severe head injury treated before the advent of CT scanning.	Exclude; EX07	PMID 6475574
FT-0626	Heiden, J S; 1983	Severe head injury. Clinical assessment and outcome.	Exclude; EX02_POPULATION	PMID 6647549
FT-0632	Stone, J L; 1983	Subdural hematomas. I. Acute subdural hematoma: progress in definition, clinical pathology, and therapy.	Core; IN02	PMID 6836474
FT-0633	Sheikh, K; 1983	Predictors of mortality and disability in stroke.	Core; IN01	PMID 6223968
FT-0635	Keane, J R; 1982	Neuro-ophthalmologic signs and symptoms of cysticercosis.	Exclude; EX02_POPULATION	PMID 7115171
FT-0636	Joogensen, E O; 1981	Natural history of global and critical brain ischaemia. Part III: cerebral prognostic signs after cardiopulmonary resuscitation. Cerebral recovery course and rate during the first year after global and critical ischaemia monitored and predicted by EEG and neurological signs.	Exclude; EX07	PMID 7255954
FT-0637	Jorgensen, E O; 1981	Natural history of global and critical brain ischaemia. Part I: EEG and neurological signs during the first year after cardiopulmonary resuscitation in patients subsequently regaining consciousness.	Core; IN01	PMID 6454948
FT-0642	Scoville, W B; 1979	Unilateral inferior temporal lobectomy with hippocampectomy for relief of incisural herniation.	Qualitative / linkage only; outside core	PMID 474209
FT-0643	Price, D J; 1979	The prediction of outcome of patients admitted following head injury in coma with bilateral fixed pupils.	Unresolved source; author-directed closure	PMID 158292
FT-0646	Tandon, P N; 1978	Temporal lobe lesions in head injury.	Exclude; EX02_POPULATION	PMID 665331
FT-0649	Subrahmanian, M V; 1975	Bilateral extradural haematomas.	Exclude; EX02_POPULATION	PMID 1139137
FT-0651	Drake, C G; 1962	Hypothermia in the Treatment of Critical Head Injury.	Exclude; EX02_POPULATION	PMID 20327281

Report ID	Author / year	Title	Current disposition	Identifiers
FT-0654	Lazaridis, Christos; 2026	Reevaluating the Effectiveness of Intracranial Pressure Monitoring in Severe Traumatic Brain Injury: Influence of Withdrawal of Life-Sustaining Treatments.	Exclude; EX07	PMID 42335082
FT-0657	Benghanem, Sarah; 2026	Correlation Between Quantitative Pupillometry and Other Markers for Neuroprognostication of Comatose Patients After Cardiac Arrest: A Bicentric Study.	Exclude; EX07	PMID 42274301 DOI 10.1097/ccm.0000000000007210
FT-0659	Marazzi, Thire Baggio Machado; 2026	Prognostic value of aneurysmal subarachnoid hemorrhage scores on mortality and disability in a Brazilian tertiary center.	Core; IN01	PMID 42119988
FT-0662	Alhamdan, Modar; 2026	Malignant Middle Cerebral Artery Infarction Decompressive Craniectomy Score: A Prognostic Model for Malignant Middle Cerebral Artery Infarction Patients Treated with Decompressive Craniectomy.	Exclude; EX07	PMID 41905467
FT-0663	Ziebart, Andreas; 2026	Multimodal non-invasive detection of delayed cerebral ischemia in patients with aneurysmal subarachnoid hemorrhage.	Core; IN	PMID 41861616
FT-0664	Jung, Min-Kyung; 2026	Cerebral Autoregulation, Mannitol Response, and Outcomes in Traumatic Brain Injury: A Structural Causal Model Approach.	Exclude; EX07	PMID 41823399
FT-0666	Mustafayev, Baglan; 2026	Demarcation Line of Tissue Compaction in Traumatic Brain Herniation: A Conceptual and Clinical Model.	Core; IN01	PMID 41697040
FT-0670	Zerbi, Simone Maria; 2026	Persistence of NPi differential predicts outcome at intensive care unit discharge: a single-center retrospective analysis.	Core; IN	PMID 41514369 DOI 10.1186/s44158-025-00317-z
FT-0671	Guo, Zonghai; 2025	Automated pupillometry predicts neurological deterioration in the neurosurgical ICU: A retrospective observational study with secondary analysis in traumatic brain injury.	Core; IN	PMID 41465989
FT-0672	Grutza, Martin; 2025	Quantitative pupillometry as a sensitive tool for detecting tumor-related mass effect in the posterior fossa: a prospective feasibility study comparing metastatic versus skull base lesions.	Exclude; EX04	PMID 41359085
FT-0674	Mitschang, Dorothea; 2025	Quantitative pupillometry as a sensitive biomarker for detecting subtle neurological impairment in mild traumatic brain injury - a pilot study.	Core; IN	PMID 40797039
FT-0675	Othman, Marwan H; 2025	Stimulants for disorders of consciousness in the intensive care unit: a randomized, placebo-controlled trial.	Core; IN	PMID 40501148
FT-0678	Chen, Weiliang; 2025	Metabolomics Analysis Reveals Potential Biomarkers for Diffuse Axonal Injury Article Category: Original Work.	Core; IN01	PMID 40195240
FT-0679	Scala, Irene; 2025	Automated pupillometry is a predictor of outcome of stroke patients: an observational, prospective, cohort study.	Core; IN	PMID 40040840

Report ID	Author / year	Title	Current disposition	Identifiers
FT-0681	Kim, Tae Jung; 2025	System for Predicting Neurological Outcomes Following Cardiac Arrest Based on Clinical Predictors Using a Machine Learning Method: The Neurological Outcomes After Cardiac Arrest Method.	Exclude; EX07	PMID 39979708
FT-0686	Lopez-De-Audicana-Jimenez-De-Aberasturi, Yolanda; 2024	Behavioral pain scales, vital signs, and pupilometry to pain assessment in the critically ill patient: A cross sectional study.	Core; IN	PMID 39571502
FT-0687	Maxin, Anthony J; 2025	Smartphone pupillometry predicts ischemic penumbra in acute ischemic stroke.	Exclude; EX07	PMID 39561861
FT-0688	Bunya, Naofumi; 2025	Signs of life as a favorable predictor for non-shockable cardiac arrest undergoing extracorporeal cardiopulmonary resuscitation post non-shockable cardiac arrest.	Core; IN01	PMID 39531887
FT-0689	Karabacak, Mert; 2025	The MOST (Mortality Score for TBI): A novel prediction model beyond CRASH-Basic and IMPACT-Core for isolated traumatic brain injury.	Core; IN01	PMID 39428266
FT-0691	Garone, Andrea; 2024	The prognostic role of Neurologic Pupil Index in COVID-19 patients: a retrospective study.	Core; IN	PMID 39381870
FT-0692	Zarifkar, Pardis; 2025	The Pupillary Light-Off Reflex in Acute Disorders of Consciousness.	Core; IN	PMID 39322845
FT-0693	Nyholm, Benjamin; 2024	Validating quantitative pupillometry thresholds for neuroprognostication after out-of-hospital cardiac arrest. A predefined substudy of the Blood Pressure and Oxygenations Targets After Cardiac Arrest (BOX)-trial.	Core; IN	PMID 39162825 DOI 10.1007/s00134-024-07574-6
FT-0694	Maxin, Anthony J; 2024	Smartphone pupillometry for detection of cerebral vasospasm after aneurysmal subarachnoid hemorrhage.	Exclude; EX07	PMID 39128501 DOI 10.1016/j.jstrokecerebrovasdis.2024.107922
FT-0695	Klug, Julian; 2024	Dynamically Normalized Pupillometry for Detecting Delayed Cerebral Ischemia After Aneurysmal Subarachnoid Hemorrhage.	Core; IN	PMID 39082834 DOI 10.1097/cce.0000000000001135
FT-0696	Benghanem, Sarah; 2024	Heart rate variability for neuro-prognostication after CA: Insight from the Parisian registry.	Exclude; EX07	PMID 38925291
FT-0697	Lapierre, Alexandra; 2024	Association Between Pupil Light Reflex and Delirium in Adults With Traumatic Brain Injury: Preliminary Findings.	Core; IN	PMID 38833515
FT-0699	Ran, Kathleen R; 2024	Evaluation of the Glasgow Coma Scale-Pupils score for predicting inpatient mortality among patients with traumatic subdural hematoma at United States trauma centers.	Core; IN01	PMID 38701532
FT-0700	Bang, Hyo Jin; 2024	Good outcome prediction after out-of-hospital cardiac arrest: A prospective multicenter observational study in Korea (the KORHN-PRO registry).	Core; IN	PMID 38582440
FT-0702	Shi, Lin; 2024	Assessment of sedation by automated pupillometry in critically ill patients: a prospective observational study.	Exclude; EX07	PMID 38429209
FT-0704	Thuccani, Meena; 2024	The capacity of neurological pupil index to predict the absence of somatosensory evoked potentials after cardiac arrest - An observational study.	Core; IN	PMID 38328749 DOI 10.1016/j.resplu.2024.100567

Report ID	Author / year	Title	Current disposition	Identifiers
FT-0705	Ambesi, Vito; 2025	The GCS-Pupils (GCS-P) score to assess outcomes after traumatic brain injury: a retrospective study.	Core; IN01	PMID 38259200
FT-0706	Banco, Pierluigi; 2024	Prediction of neurocritical care intensity through automated infrared pupillometry and transcranial doppler in blunt traumatic brain injury: the NOPE study.	Exclude; EX07	PMID 38226989 DOI 10.1007/s00068-023-02435-1
FT-0707	Lenga, Pavlina; 2024	The use of quantitative pupillometry in brain death determination: preliminary findings	Core; IN	PMID 38082049 DOI 10.1007/s10072-023-07251-4
FT-0708	Casey, Flora; 2023	Automated pupillometry and optic nerve sheath diameter ultrasound to define tuberculous meningitis disease severity and prognosis.	Core; IN	PMID 37722232
FT-0710	Eaton, Jessica C; 2023	Acute Extra-Arachnoid Subdural Hematomas in Patients 50 Years and Older: When Subdurals Act Like Epidurals.	Core; IN01	PMID 37683917
FT-0711	Oddo, Mauro; 2023	The Neurological Pupil index for outcome prognostication in people with acute brain injury (ORANGE): a prospective, observational, multicentre cohort study	Core; IN	PMID 37652068 DOI 10.1016/S1474-4422(23)00271-5
FT-0712	Lee, Seohee; 2023	Intraoperative neurological pupil index and postoperative delirium and neurologic adverse events after cardiac surgery: an observational study.	Core; IN	PMID 37620412
FT-0715	Cleri, Nathaniel A; 2023	Predicting traumatic brain injury outcomes using a posterior dominant rhythm.	Core; IN01	PMID 37329521
FT-0716	Kossel, Clara-Sophie; 2023	Pupillometry in the follow-up of patients undergoing EVT - prediction of space-occupying hemispheric infarction.	Exclude; EX07	PMID 37300717 DOI 10.1007/s00415-023-11797-w
FT-0717	Lele, Abhijit V; 2023	Neurological Pupillary Index and Disposition at Hospital Discharge following ICU Admission for Acute Brain Injury.	Core; IN	PMID 37298001
FT-0718	Bertotti, Melina More; 2023	Glasgow coma scale pupil score (GCS-P) and the hospital mortality in severe traumatic brain injury: analysis of 1,066 Brazilian patients.	Exclude; EX07	PMID 37257465
FT-0719	Nyholm, Benjamin; 2023	Specific thresholds of quantitative pupillometry parameters predict unfavorable outcome in comatose survivors early after cardiac arrest.	Core; IN	PMID 37252025 DOI 10.1016/j.resplu.2023.100399
FT-0720	Gouvea Bogossian, Elisa; 2023	Neurological Pupil Index and Delayed Cerebral Ischemia after Subarachnoid Hemorrhage: A Retrospective Multicentric Study.	Core; IN	PMID 37225941 DOI 10.1007/s12028-023-01744-y
FT-0722	Vanat, Aurelien; 2023	Multimodal Prediction of Favorable Outcome After Cardiac Arrest: A Cohort Study.	Exclude; EX07	PMID 36951448
FT-0723	Pease, Matthew; 2023	Association of Posttraumatic Epilepsy With Long-term Functional Outcomes in Individuals With Severe Traumatic Brain Injury.	Exclude; EX07	PMID 36948595
FT-0724	Wilson, Laura D; 2023	Prevalence and Risk Factors for Intensive Care Unit Delirium After Traumatic Brain Injury: A Retrospective Cohort Study.	Exclude; EX07	PMID 36720836

Report ID	Author / year	Title	Current disposition	Identifiers
FT-0725	Kim, Ivy So Yeon; 2022	Quantitative pupillometry and radiographic markers of intracranial midline shift: A pilot study.	Exclude; EX07	PMID 36561299 DOI 10.3389/fneur.2022.1046548
FT-0727	Su, Tsung-Ming; 2023	Vertex epidural hematoma: Diagnosis, therapeutic consideration and outcome.	Core; IN02	PMID 36411102
FT-0730	Wang, Yu-Mei; 2022	The combination of arterial lactate level with GCS-pupils score to evaluate short term prognosis in traumatic brain injury: a retrospective study.	Core; IN01	PMID 36380277
FT-0731	Wilson, Laura D; 2023	Dynamic predictors of in-hospital and 3-year mortality after traumatic brain injury: A retrospective cohort study.	Exclude; EX07	PMID 36372578
FT-0733	Peluso, Lorenzo; 2022	Neurological pupil index and its association with other prognostic tools after cardiac arrest: A post hoc analysis.	Core; IN	PMID 35914656 DOI 10.1016/j.resuscitation.2022.07.030
FT-0734	Privitera, Claudio M; 2022	A differential of the left eye and right eye neurological pupil index is associated with discharge modified Rankin scores in neurologically injured patients.	Exclude; EX07	PMID 35869429
FT-0735	Romagnosi, Federico; 2022	Neurological Pupil Index for the Early Prediction of Outcome in Severe Acute Brain Injury Patients.	Core; IN	PMID 35624996 DOI 10.3390/brainsci12050609
FT-0736	Kamal, Abdulkadir; 2022	Pilot Study of Neurologic Pupil Index as A Predictor of External Ventricular Drain Clamp Trial Failure After Subarachnoid Hemorrhage.	Core; IN	PMID 35525437
FT-0737	Macchini, Elisabetta; 2022	Pain pupillary index to prognosticate unfavorable outcome in comatose cardiac arrest patients.	Core; IN	PMID 35504344 DOI 10.1016/j.resuscitation.2022.04.026
FT-0738	Paramanathan, Sansuthan; 2022	Quantitative pupillometry in comatose out-of-hospital cardiac arrest patients: A post-hoc analysis of the TTH48 trial.	Core; IN	PMID 35488868 DOI 10.1111/aas.14078
FT-0739	Thamminaina, Abhinov; 2022	The impact of alcohol intoxication on early Glasgow Coma Scale-Pupil reactivity score in patients with traumatic brain injury: A prospective observational study.	Core; IN01	PMID 35433390
FT-0740	Johnson, Mark D; 2022	An independent, external validation and component analysis of the Surviving Penetrating Injury to the Brain score for civilian cranial gunshot injuries.	Core; IN01	PMID 35426813
FT-0741	Warren, Alex; 2022	Early quantitative infrared pupillometry for prediction of neurological outcome in patients admitted to intensive care after out-of-hospital cardiac arrest.	Exclude; EX07	PMID 35256151 DOI 10.1016/j.bja.2021.12.052
FT-0742	Sharma, Rahul A; 2022	Prognostic Value of the Neurological Pupil Index in Patients With Acute Subarachnoid Hemorrhage.	Core; IN	PMID 35195545
FT-0743	El Boujdaini, Adil; 2022	Prognostic role of automatic pupillometry in sepsis: a retrospective study.	Core; IN	PMID 35191640
FT-0745	Luz Teixeira, Thomas; 2021	Early Pupillometry Assessment in Traumatic Brain Injury Patients: A Retrospective Study.	Core; IN	PMID 34942959 DOI 10.3390/brainsci11121657

Report ID	Author / year	Title	Current disposition	Identifiers
FT-0746	Nourelahi, Mehdi; 2022	A machine learning model for predicting favorable outcome in severe traumatic brain injury patients after 6 months.	Exclude; EX02_POPULATION	PMID 34762793
FT-0747	Kashkoush, Ahmed; 2022	Predictors of Mortality, Withdrawal of Life-Sustaining Measures, and Discharge Disposition in Octogenarians with Subdural Hematomas.	Core; IN01	PMID 34626845
FT-0748	Menozzi, Marco; 2022	Early Neurological Pupil Index Assessment to Predict Outcome in Cardiac Arrest Patients Undergoing Extracorporeal Membrane Oxygenation.	Core; IN	PMID 34494986 DOI 10.1097/mat.0000000000001569
FT-0749	Traylor, Jeffrey I; 2021	Quantitative pupillometry in patients with traumatic brain injury and loss of consciousness: A prospective pilot study.	Core; IN	PMID 34373065
FT-0750	Ercole, Ari; 2021	Imputation strategies for missing baseline neurological assessment covariates after traumatic brain injury: A CENTER-TBI study.	Exclude; EX02_POPULATION	PMID 34358231
FT-0751	Peluso, Lorenzo; 2021	Multimodal Approach to Predict Neurological Outcome after Cardiac Arrest: A Single-Center Experience.	Core; IN	PMID 34356123
FT-0752	Godau, Jana; 2022	Automated Pupillometry for Assessment of Treatment Success in Nonconvulsive Status Epilepticus.	Core; IN	PMID 34331202
FT-0753	Darby, Joseph M; 2021	Reliability of the Telemedicine Examination in the Neurologic Diagnosis of Death.	Exclude; EX07	PMID 33968467
FT-0754	Podolsky-Gondim, Guilherme G; 2021	Traumatic Brain Injury in the Elderly: Clinical Features, Prognostic Factors, and Outcomes of 133 Consecutive Surgical Patients.	Exclude; EX04	PMID 33815990
FT-0756	Lin Y; 2021	The prediction value of Glasgow coma scale-pupils score in neurocritical patients: a retrospective study.	Exclude; EX07	PMID 33645359 DOI 10.1080/02699052.2021.1890821
FT-0757	Zhao, Zirun; 2021	Cardiac arrest after severe traumatic brain injury can be survivable with good outcomes.	Core; IN01	PMID 33634211
FT-0758	Kamal, Vineet Kumar; 2021	Development and temporal external validation of a simple risk score tool for prediction of outcomes after severe head injury based on admission characteristics from level-1 trauma centre of India using retrospectively collected data.	Exclude; EX07	PMID 33455929
FT-0759	Yang C; 2021	Effects of Primary Decompressive Craniectomy on the Outcomes of Serious Traumatic Brain Injury with Mass Lesions, and Independent Predictors of Operation Decision.	Core; IN01	PMID 33422716 DOI 10.1016/j.wneu.2020.12.158
FT-0760	Battaglini, Denise; 2020	Neurological Complications and Noninvasive Multimodal Neuromonitoring in Critically Ill Mechanically Ventilated COVID-19 Patients.	Core; IN	PMID 33329361
FT-0761	Avsenik J; 2021	Prognostic value of CT perfusion and permeability imaging in traumatic brain injury.	Exclude; EX07	PMID 33009337 DOI 10.1097/TA.0000000000002964

Report ID	Author / year	Title	Current disposition	Identifiers
FT-0762	Kerezoudis, Panagiotis; 2020	Morbidity and mortality in elderly patients undergoing evacuation of acute traumatic subdural hematoma.	Core; IN01	PMID 33002870
FT-0763	Li, Xia; 2020	Establishment and validation of a model for brain injury state evaluation and prognosis prediction.	Exclude; EX02_POPULATION	PMID 32928607
FT-0764	Yic, Christian D; 2020	Comparison of ultrasonographic versus infrared pupillary assessment.	Core; IN	PMID 32794115
FT-0765	Gu, Jia-Cheng; 2020	Intracranial Pressure during External Ventricular Drainage Weaning Is an Outcome Predictor of Traumatic Brain Injury.	Exclude; EX07	PMID 32695822
FT-0766	Siepen, Bernhard M; 2020	Neuromonitoring Using Neurosonography and Pupillometry in A Weaning and Early Neurorehabilitation Unit.	Exclude; EX04	PMID 32592294
FT-0767	Oh SH; 2020	Prognostic Value of P25/30 Cortical Somatosensory Evoked Potential Amplitude After Cardiac Arrest.	Core; IN	PMID 32568854 DOI 10.1097/CCM.0000000000004460
FT-0768	Obinata, Hirofumi; 2020	Early automated infrared pupillometry is superior to auditory brainstem response in predicting neurological outcome after cardiac arrest.	Core; IN	PMID 32531404
FT-0769	Olsen, Markus Harboe; 2020	Automated pupillometry and the FOUR score - what is the diagnostic benefit in neurointensive care?	Core; IN	PMID 32383011
FT-0771	Okidi, R; 2020	Factors affecting mortality after traumatic brain injury in a resource-poor setting.	Exclude; EX02_POPULATION	PMID 32207576
FT-0772	Tamura, Tomoyoshi; 2020	Early outcome prediction with quantitative pupillary response parameters after out-of-hospital cardiac arrest: A multicenter prospective observational study	Core; IN	PMID 32191709 DOI 10.1371/journal.pone.0228224
FT-0773	Liu J; 2020	Predicting Long-Term Outcomes After Poor-Grade Aneurysmal Subarachnoid Hemorrhage Using Decision Tree Modeling.	Core; IN01	PMID 32171016 DOI 10.1093/neuros/nyaa052
FT-0775	Favre, Eva; 2020	Neuromonitoring of delirium with quantitative pupillometry in sedated mechanically ventilated critically ill patients.	Core; IN	PMID 32093710
FT-0776	Amorim, Robson Luis; 2020	Prediction of Early TBI Mortality Using a Machine Learning Approach in a LMIC Population.	Exclude; EX07	PMID 32038454
FT-0777	Miroz, John-Paul; 2020	Neurological Pupil index for Early Prognostication After Venoarterial Extracorporeal Membrane Oxygenation	Core; IN	PMID 31870911 DOI 10.1016/j.chest.2019.11.037
FT-0778	Silva Neto, Angelo R; 2019	Transcalvarial brain herniation volume as a predictor of posttraumatic hydrocephalus after decompressive craniectomy.	Exclude; EX02_POPULATION	PMID 31096109
FT-0779	Mikati, Abdul Ghani; 2019	Multicenter Validation of the Survival After Acute Civilian Penetrating Brain Injuries (SPIN) Score.	Core; IN01	PMID 31065707

Report ID	Author / year	Title	Current disposition	Identifiers
FT-0782	Dawes, Aaron J; 2019	Injury-specific variables improve risk adjustment and hospital quality assessment in severe traumatic brain injury.	Core; IN01	PMID 30958810
FT-0783	Khalili, Hosseinali; 2019	Determinants of reoperation after decompressive craniectomy in patients with traumatic brain injury: A comparative study.	Core; IN	PMID 30954701
FT-0784	Obling, Laust; 2020	Prognostic value of automated pupillometry: an unselected cohort from a cardiac intensive care unit.	Exclude; EX07	PMID 30950641
FT-0785	Mangat, Halinder S; 2020	Hypertonic Saline is Superior to Mannitol for the Combined Effect on Intracranial Pressure and Cerebral Perfusion Pressure Burdens in Patients With Severe Traumatic Brain Injury.	Exclude; EX07	PMID 30877299
FT-0786	Aoun, Salah G; 2020	Detection of delayed cerebral ischemia using objective pupillometry in patients with aneurysmal subarachnoid hemorrhage.	Exclude; EX07	PMID 30641848
FT-0787	Para, Reyaz Ahmed; 2018	Our Experience with Management and Outcome of Isolated Traumatic Brain Injury Patients Admitted in Intensive Care Unit.	Core; IN01	PMID 30568372
FT-0788	Oddo, Mauro; 2018	Quantitative versus standard pupillary light reflex for early prognostication in comatose cardiac arrest patients: an international prospective multicenter double-blinded study.	Core; IN	PMID 30478620 DOI 10.1007/s00134-018-5448-6
FT-0789	Bus, Sander; 2019	Do older patients with acute or subacute subdural hematoma benefit from surgery?	Core; IN01	PMID 30317874
FT-0790	Natzeder, Samuel; 2019	Portable Infrared Pupillometer in Patients With Subarachnoid Hemorrhage: Prognostic Value and Circadian Rhythm of the Neurological Pupil Index (NPi).	Exclude; EX07	PMID 30303882
FT-0791	Ding, Wu; 2018	Development and validation of the hypertensive intracerebral hemorrhage prognosis models.	Exclude; EX07	PMID 30278523
FT-0793	Tamura, Tomoyoshi; 2018	Quantitative assessment of pupillary light reflex for early prediction of outcomes after out-of-hospital cardiac arrest: A multicentre prospective observational study.	Core; IN	PMID 29958957
FT-0794	Nasi, Davide; 2018	Decompressive Craniectomy for Traumatic Brain Injury: The Role of Cranioplasty and Hydrocephalus on Outcome.	Exclude; EX02_POPULATION	PMID 29772371
FT-0795	Darwazeh, Rami; 2018	Traumatic Brain Injury Caused by Missile Wounds in the North of Palestine: A Single Institution's Experience with 520 Consecutive Civilian Patients.	Exclude; EX02_POPULATION	PMID 29738860
FT-0796	Murray, Gordon D; 2018	Simplifying the use of prognostic information in traumatic brain injury. Part 2: Graphical presentation of probabilities.	Core; IN02	PMID 29631517
FT-0797	Brennan, Paul M; 2018	Simplifying the use of prognostic information in traumatic brain injury. Part 1: The GCS-Pupils score: an extended index of clinical severity.	Exclude; EX02_POPULATION	PMID 29631516
FT-0798	Sinnah, Fabrice; 2018	Early Electroencephalography Findings in Cardiogenic Shock Patients Treated by Venoarterial Extracorporeal Membrane Oxygenation.	Exclude; EX07	PMID 29389771

Report ID	Author / year	Title	Current disposition	Identifiers
FT-0799	Mangalore, Sandhya; 2017	Can Transcranial Color Doppler Spectral Signatures be a Novel Biomarker for Monitoring Cerebrovascular Autoregulation and Intracranial Pressure? A Speculative Synthesis.	Exclude; EX07	PMID 28904458
FT-0800	Han, Julian Xinguang; 2017	Models of Mortality and Morbidity in Severe Traumatic Brain Injury: An Analysis of a Singapore Neurotrauma Database.	Exclude; EX07	PMID 28867312
FT-0801	McNett, Molly; 2017	Correlations Between Hourly Pupillometer Readings and Intracranial Pressure Values.	Core; IN	PMID 28661946
FT-0802	Reynolds, Alexandra S; 2017	Post-anoxic quantitative MRI changes may predict emergence from coma and functional outcomes at discharge.	Exclude; EX07	PMID 28624592
FT-0803	Solari, Daria; 2017	Early prediction of coma recovery after cardiac arrest with blinded pupillometry.	Exclude; EX07	PMID 28470675
FT-0804	Vathanalaoha, Karin; 2017	Predictive Factors of Survival and 6-Month Favorable Outcome of Very Severe Head Trauma Patients; a Historical Cohort Study.	Exclude; EX02_POPULATION	PMID 28286831
FT-0805	Yang, Eric; 2018	Infrared pupillometry helps to detect and predict delirium in the post-anesthesia care unit	Core; IN	PMID 28275978 DOI 10.1007/s10877-017-0009-z
FT-0806	Khalili, Hosseinali; 2017	Effects of cerebrolysin on functional recovery in patients with severe disability after traumatic brain injury: A historical cohort study.	Exclude; EX02_POPULATION	PMID 27871029
FT-0807	Cicuendez, Marta; 2017	Prognostic value of corpus callosum injuries in severe head trauma.	Exclude; EX07	PMID 27796652
FT-0808	Muehlschlegel, Susanne; 2016	Predicting survival after acute civilian penetrating brain injuries: The SPIN score.	Core; IN01	PMID 27784772
FT-0809	Rizoli, Sandro; 2016	Early prediction of outcome after severe traumatic brain injury: a simple and practical model.	Exclude; EX07	PMID 27553436
FT-0810	Fountain, Daniel M; 2017	Survival Trends After Surgery for Acute Subdural Hematoma in Adults Over a 20-year Period.	Exclude; EX07	PMID 27172128
FT-0811	Khajavikhan, Javaher; 2016	Outcome and Predicting Factor Following Severe Traumatic Brain Injury: A Retrospective Cross-Sectional Study.	Core; IN01	PMID 27042518
FT-0812	Kamal, Vineet Kumar; 2016	Prognostic models for prediction of outcomes after traumatic brain injury based on patients admission characteristics.	Exclude; EX07	PMID 27003280
FT-0813	Kasprowicz, Magdalena; 2016	A comparison of the Full Outline of UnResponsiveness (FOUR) score and Glasgow Coma Score (GCS) in predictive modelling in traumatic brain injury.	Exclude; EX07	PMID 27001246
FT-0814	van der Ploeg, Tjeerd; 2016	Modern modeling techniques had limited external validity in predicting mortality from traumatic brain injury.	Exclude; EX07	PMID 26987507
FT-0815	Heimburger, Delphine; 2016	Quantitative pupillometry and transcranial Doppler measurements in patients treated with hypothermia after cardiac arrest.	Core; IN	PMID 26970030

Report ID	Author / year	Title	Current disposition	Identifiers
FT-0816	Huang, Yu-Hua; 2016	Prognostic Impact of Intracranial Pressure Monitoring After Primary Decompressive Craniectomy for Traumatic Brain Injury.	Exclude; EX02_POPULATION	PMID 26732966
FT-0817	Roe, Cecilie; 2015	Mortality and One-Year Functional Outcome in Elderly and Very Old Patients with Severe Traumatic Brain Injuries: Observed and Predicted.	Core; IN01	PMID 26688614
FT-0818	Ozyurt, Erhan; 2015	Retrospective Analysis of Prognostic Factors of Severe Traumatic Brain Injury in a University Hospital in Turkey.	Core; IN01	PMID 26617136
FT-0819	Park, Jeong Goo; 2015	Clinical Utility of an Automated Pupillometer in Patients with Acute Brain Lesion.	Core; IN	PMID 26587191
FT-0820	Takala, Riikka S K; 2016	Glial Fibrillary Acidic Protein and Ubiquitin C-Terminal Hydrolase-L1 as Outcome Predictors in Traumatic Brain Injury.	Exclude; EX07	PMID 26547005
FT-0822	Roger, Claire; 2015	Neuron specific enolase and Glasgow motor score remain useful tools for assessing neurological prognosis after out-of-hospital cardiac arrest treated with therapeutic hypothermia.	Core; IN01	PMID 26324761
FT-0823	Olgun, Gokhan; 2015	Pupillometry in brain death: differences in pupillary diameter between paediatric and adult subjects.	Core; IN	PMID 26184095
FT-0825	Meguro, Toshinari; 2015	Primary pontine hemorrhage in the acute stage: clinical features and a proposed new simple scoring system.	Core; IN01	PMID 25724243
FT-0826	Dawes, Aaron J; 2015	Intracranial pressure monitoring and inpatient mortality in severe traumatic brain injury: A propensity score-matched analysis.	Exclude; EX07	PMID 25710418
FT-0827	Hu, Yu; 2015	Acute bilateral mass-occupying lesions in non-penetrating traumatic brain injury: a retrospective study.	Exclude; EX07	PMID 25618576
FT-0828	Mangat, Halinder S; 2015	Hypertonic saline reduces cumulative and daily intracranial pressure burdens after severe traumatic brain injury.	Exclude; EX07	PMID 25380107
FT-0829	Gomez, Pedro A; 2014	Validation of a prognostic score for early mortality in severe head injury cases.	Exclude; EX07	PMID 25237737
FT-0830	Majdan, Marek; 2015	Glasgow coma scale motor score and pupillary reaction to predict six-month mortality in patients with traumatic brain injury: comparison of field and admission assessment.	Exclude; EX07	PMID 25227136
FT-0831	Sobuwa, Simpiwe; 2014	Predicting outcome in severe traumatic brain injury using a simple prognostic model.	Exclude; EX07	PMID 25214051
FT-0832	Suys, Tamarah; 2014	Automated Quantitative Pupillometry for the Prognostication of Coma After Cardiac Arrest	Core; IN	PMID 24760270 DOI 10.1007/s12028-014-9981-z
FT-0833	Bulstrode, Harry; 2014	Mitochondrial DNA and traumatic brain injury.	Exclude; EX02_POPULATION	PMID 24523223

Report ID	Author / year	Title	Current disposition	Identifiers
FT-0834	Izzy, Saef; 2013	Self-fulfilling prophecies through withdrawal of care: do they exist in traumatic brain injury, too?	Exclude; EX07	PMID 24132565
FT-0835	Rodriguez, Miguel; 2013	Predicting mortality from head injury: experience of Sancti Spiritus Province, Cuba.	Exclude; EX07	PMID 23934424
FT-0837	Lesko, Mehdi Moazzez; 2013	Models of mortality probability in severe traumatic brain injury: results of the modelling by the UK trauma registry.	Exclude; EX07	PMID 23865489
FT-0838	Mejaddam, Ali Y; 2013	Prolonged emergency department length of stay is not associated with worse outcomes in traumatic brain injury.	Core; IN01	PMID 23769388
FT-0839	Leitgeb, Johannes; 2013	Impact of concomitant injuries on outcomes after traumatic brain injury.	Exclude; EX07	PMID 23463257
FT-0841	Hoffmann, Michael; 2012	Introduction of a novel trauma score.	Exclude; EX07	PMID 23188250
FT-0842	Subaiya, Saleena; 2012	Predicting intracranial hemorrhage after traumatic brain injury in low and middle-income countries: a prognostic model based on a large, multi-center, international cohort.	Exclude; EX07	PMID 23157693
FT-0843	Egea-Guerrero, J J; 2012	Clinical variables and neuromonitoring information (intracranial pressure and brain tissue oxygenation) as predictors of brain-death development after severe traumatic brain injury.	Exclude; EX07	PMID 22974906
FT-0844	Mezue, Wilfred C; 2012	Traumatic extradural hematoma in enugu, Nigeria.	Exclude; EX02_POPULATION	PMID 24027399
FT-0845	Yuan, Fang; 2012	Predicting outcomes after traumatic brain injury: the development and validation of prognostic models based on admission characteristics.	Core; IN01	PMID 22743383
FT-0846	Lesko, M M; 2012	Prognostic value of various intracranial pathologies in traumatic brain injury.	Exclude; EX07	PMID 26815669
FT-0847	Greer, David M; 2012	Clinical examination for outcome prediction in nontraumatic coma.	Exclude; EX07	PMID 22020245
FT-0848	Panczykowski, David M.; 2012	Prospective Independent Validation of IMPACT Modeling as a Prognostic Tool in Severe Traumatic Brain Injury	Exclude; EX07	PMID 21933014 DOI 10.1089/neu.2010.1482
FT-0849	Turgeon, Alexis F; 2011	Mortality associated with withdrawal of life-sustaining therapy for patients with severe traumatic brain injury: a Canadian multicentre cohort study.	Exclude; EX07	PMID 21876014
FT-0850	Fugate JE; 2010	Predictors of neurologic outcome in hypothermia after cardiac arrest.	Core; IN01	PMID 21061401 DOI 10.1002/ana.22133
FT-0851	Rittenberger, Jon C; 2010	Association between clinical examination and outcome after cardiac arrest.	Core; IN01	PMID 20732605
FT-0852	Ho, Kwok M; 2010	Extracranial injuries are important in determining mortality of neurotrauma.	Exclude; EX07	PMID 20526198
FT-0853	Petroni, Gustavo; 2010	Early prognosis of severe traumatic brain injury in an urban argentinian trauma center.	Exclude; EX02_POPULATION	PMID 20220417

Report ID	Author / year	Title	Current disposition	Identifiers
FT-0854	Hofbauer, Marcus; 2010	Predictive factors influencing the outcome after gunshot injuries to the head-a retrospective cohort study.	Core; IN02	PMID 20173654
FT-0855	Petridis, A K; 2009	Acute subdural hematoma in the elderly; clinical and CT factors influencing the surgical treatment decision.	Core; IN01	PMID 19711259
FT-0856	Nijboer, Johanna M M; 2010	Patients beyond salvation? Various categories of trauma patients with a minimal Glasgow Coma Score.	Exclude; EX02_POPULATION	PMID 19570534
FT-0859	Abe, Toshikazu; 2009	Predictors for good cerebral performance among adult survivors of out-of-hospital cardiac arrest.	Core; IN01	PMID 19185409
FT-0860	Daubin, Cedric; 2008	A clinical and EEG scoring system that predicts early cortical response (N20) to somatosensory evoked potentials and outcome after cardiac arrest.	Exclude; EX07	PMID 19055810
FT-0861	Steyerberg, Ewout W.; 2008	Predicting Outcome after Traumatic Brain Injury: Development and International Validation of Prognostic Scores Based on Admission Characteristics	Exclude; EX07	PMID 18684008 DOI 10.1371/journal.pmed.0050165
FT-0862	Perel, Pablo; 2008	Predicting outcome after traumatic brain injury: practical prognostic models based on large cohort of international patients.	Exclude; EX07	PMID 18270239
FT-0863	Mack, William J; 2008	Pupillary reactivity upon hospital admission predicts long-term outcome in poor grade aneurysmal subarachnoid hemorrhage patients.	Core; IN01	PMID 18058256
FT-0864	Thompson, Hilaire J; 2008	Evaluation of the effect of intensity of care on mortality after traumatic brain injury.	Core; IN01	PMID 18007264
FT-0865	Sherer, M; 2008	Comparison of indices of traumatic brain injury severity: Glasgow Coma Scale, length of coma and post-traumatic amnesia.	Exclude; EX07	PMID 17928328
FT-0866	Murray, Gordon D; 2007	Multivariable prognostic analysis in traumatic brain injury: results from the IMPACT study.	Exclude; EX02	PMID 17375997
FT-0867	Cremer, Olaf L; 2006	Prognosis following severe head injury: Development and validation of a model for prediction of death, disability, and functional recovery.	Exclude; EX07	PMID 16983303
FT-0868	Hernandez, Adrian V; 2006	Adjustment for strong predictors of outcome in traumatic brain injury trials: 25% reduction in sample size requirements in the IMPACT study.	Exclude; EX07	PMID 16958582
FT-0870	Fischer, Catherine; 2006	Improved prediction of awakening or nonawakening from severe anoxic coma using tree-based classification analysis.	Exclude; EX02_POPULATION	PMID 16557163
FT-0872	Hukkelhoven, Chantal W P M; 2005	Predicting outcome after traumatic brain injury: development and validation of a prognostic score based on admission characteristics.	Exclude; EX07	PMID 16238481
FT-0873	Luaute, Jacques; 2005	Late auditory and event-related potentials can be useful to predict good functional outcome after coma.	Exclude; EX02_POPULATION	PMID 15895337

Report ID	Author / year	Title	Current disposition	Identifiers
FT-0874	Hukkelhoven, Chantal W P M; 2005	Admission of patients with severe and moderate traumatic brain injury to specialized ICU facilities: a search for triage criteria.	Exclude; EX07	PMID 15834705
FT-0875	Fischer, Catherine; 2004	Predictive value of sensory and cognitive evoked potentials for awakening from coma.	Exclude; EX02_POPULATION	PMID 15326240
FT-0876	Rovlias, A; 2004	Classification and regression tree for prediction of outcome after severe head injury using simple clinical and laboratory variables.	Core; IN02	PMID 15307901
FT-0877	Ariza, Mar; 2004	Influence of extraneurological insults on ventricular enlargement and neuropsychological functioning after moderate and severe traumatic brain injury.	Exclude; EX02_POPULATION	PMID 15307899
FT-0878	Isa, Ruwaida; 2003	Outcome of severe traumatic brain injury: comparison of three monitoring approaches.	Exclude; EX02_POPULATION	PMID 15305837
FT-0879	Myrianthefs, Pavlos; 2003	Efficacy of CPR in a general, adult ICU.	Exclude; EX07	PMID 12668298
FT-0881	Hukkelhoven, Chantal W P M; 2002	Regional differences in patient characteristics, case management, and outcomes in traumatic brain injury: experience from the tirilazad trials.	Exclude; EX07	PMID 12296638
FT-0882	Cruz, J; 2001	Improving clinical outcomes from acute subdural hematomas with the emergency preoperative administration of high doses of mannitol: a randomized trial.	Exclude; EX07	PMID 11564247
FT-0883	Azian, A A; 2001	Computed tomography of the brain in predicting outcome of traumatic intracranial haemorrhage in Malaysian patients.	Exclude; EX02_POPULATION	PMID 11534693
FT-0884	Cordato, D J; 2001	Prolonged thiopentone infusion for neurosurgical emergencies: usefulness of therapeutic drug monitoring.	Core; IN02	PMID 11512643
FT-0885	Claassen, J; 2001	Early recovery after closed traumatic head injury: somatosensory evoked potentials and clinical findings.	Exclude; EX02_POPULATION	PMID 11373410
FT-0886	Rovlias, A; 2001	The blood leukocyte count and its prognostic significance in severe head injury.	Exclude; EX07	PMID 11358584
FT-0887	Missori, P; 2000	Spontaneous acute subdural hematomas. A clinical comparison with traumatic acute subdural hematomas.	Core; IN01	PMID 10949446
FT-0888	Lannoo, E; 2000	Early predictors of mortality and morbidity after severe closed head injury.	Exclude; EX07	PMID 10833059
FT-0889	Rovlias A; 2000	The influence of hyperglycemia on neurological outcome in patients with severe head injury.	Exclude; EX02_POPULATION	PMID 10690722 DOI 10.1097/00006123-200002000-00015
FT-0890	Wu, J J; 1999	Surgical outcome of traumatic intracranial hematoma at a regional hospital in Taiwan.	Exclude; EX02_POPULATION	PMID 10421184

Report ID	Author / year	Title	Current disposition	Identifiers
FT-0891	Signorini, D F; 1999	Predicting survival using simple clinical variables: a case study in traumatic brain injury.	Exclude; EX07	PMID 9886445
FT-0892	Kilaru, S; 1996	Long-term functional status and mortality of elderly patients with severe closed head injuries.	Core; IN01	PMID 8970546
FT-0893	Mamelak, A N; 1996	Predicting survival from head trauma 24 hours after injury: a practical method with therapeutic implications.	Exclude; EX02_POPULATION	PMID 8676429
FT-0896	Jamjoom, A; 1992	Justification for evacuating acute subdural haematomas in patients above the age of 75 years.	Core; IN01	PMID 1286901
FT-0897	Feldman, Z; 1991	[Outcome of immediate management of severe head injuries].	Unresolved source; author-directed closure	PMID 1748352
FT-0899	Luerssen, T G; 1988	Outcome from head injury related to patient's age. A longitudinal prospective study of adult and pediatric head injury.	Exclude; EX02_POPULATION	PMID 3343613
FT-0900	Braakman, R; 1986	Prognosis and prediction of outcome in comatose head injured patients.	Exclude; EX01_PUBLICATION_TYPE	PMID 3541506
FT-0905	Longstreth WT Jr; 1983	Prediction of awakening after out-of-hospital cardiac arrest.	Exclude; EX07	PMID 6843631 DOI 10.1056/NEJM198306093082302
FT-0907	Clifton, G L; 1980	Neurological course and correlated computerized tomography findings after severe closed head injury.	Exclude; EX02_POPULATION	PMID 7373389
FT-0909	van den Berge, J H; 1979	Interobserver agreement in assessment of ocular signs in coma.	Exclude; EX07	PMID 533856
FT-0912	Sanvito, W L; 1978	[Prognostic-evolutive evaluation of patients in coma based on ocular reflexes].	Exclude; EX07	PMID 655897
FT-0914	Jennett, B; 1977	Aspects of coma after severe head injury.	Exclude; EX02_POPULATION	PMID 67287
FT-0915	Bhat, Shashidhar; 2025	Psychiatric aspects of mass datura poisoning.	Exclude; EX02_POPULATION	PMID 41180048
FT-0920	Biswas, Subrata; 2022	Is Serum Creatine Phosphokinase Level Useful to Predict the Severity of Organophosphate Poisoning?	Exclude; EX07	PMID 36381386
FT-0921	Kaufman, Aaron R; 2022	Pharmacologic Mydriasis Secondary to Topical Glycopyrronium Tosylate Cloths: Clinical Characterization From a Multicenter Analysis.	Core; IN02	PMID 35427257
FT-0922	Soulis, Dimitrios; 2022	The effect of atropine used in dobutamine stress echocardiography on pupil diameter.	Exclude; EX04	PMID 35088615
FT-0925	Sadaka, Ama; 2017	Evaluation of Horner Syndrome in the MRI Era.	Core; IN01	PMID 28445191
FT-0928	Silva, Julio Cesar Santos da; 2016	Factors associated with the deaths of men poisoned by carbamato ("chumbinho").	Core; IN01	PMID 27253592

Report ID	Author / year	Title	Current disposition	Identifiers
FT-0931	Park, Sun Young; 2015	Transient Horner's syndrome following thoracic epidural anesthesia for mastectomy: a prospective observational study.	Core; IN	PMID 25560203
FT-0932	Walton, Ryan N; 2014	Outbreak of type E foodborne botulism linked to traditionally prepared salted fish in Ontario, Canada.	Exclude; EX07	PMID 25188279
FT-0933	Gheshlaghi, Farzad; 2012	Evaluation of serum sodium changes in tricyclic antidepressants toxicity and its correlation with electrocardiography, serum pH, and toxicity severity.	Exclude; EX02_POPULATION	PMID 23326798
FT-0934	Gonzalez Martin-Moro, J; 2012	[Everyday anisocoria: anisocoria epidemiology in a secondary care setting].	Core; IN	PMID 22732119
FT-0936	Veale, Denise Joy; 2011	Amitraz poisoning in South Africa: a two year survey (2008-2009).	Exclude; EX02_POPULATION	PMID 21288150
FT-0937	Trinh, Kiem Xuan; 2010	Hyponatraemia, rhabdomyolysis, alterations in blood pressure and persistent mydriasis in patients envenomed by Malayan kraits (Bungarus candidus) in southern Viet Nam.	Exclude; EX07	PMID 20637219
FT-0938	Hill, S L; 2010	Electrocardiographic effects of methylphenidate overdose.	Exclude; EX02_POPULATION	PMID 20507245
FT-0939	Neghina, Adriana Maria; 2010	Foodborne botulism in southwest Romania during the post-communism period 1990-2007.	Exclude; EX02_POPULATION	PMID 19535277
FT-0940	Galldiks, Norbert; 2007	Rapid geographical clustering of wound botulism in Germany after subcutaneous and intramuscular injection of heroin.	Core; IN01	PMID 17356188
FT-0941	Koc, F; 2005	The sensitivity and specificity of 0.5% apraclonidine in the diagnosis of oculosympathetic paresis.	Exclude; EX07	PMID 16234449
FT-0942	Rahman, I; 2006	Open globe injuries: factors predictive of poor outcome.	Exclude; EX02_POPULATION	PMID 16179934
FT-0944	Boyd J; 2003	Serious overdoses involving buprenorphine in Helsinki.	Core; IN01	PMID 12904197 DOI 10.1034/j.1399-6576.2003.00201.x
FT-0945	Okudera, Hiroshi; 2002	Clinical features on nerve gas terrorism in Matsumoto.	Exclude; EX07	PMID 11749011
FT-0947	Maselli, R A; 1997	Cluster of wound botulism in California: clinical, electrophysiologic, and pathologic study.	Qualitative / linkage only; outside core	PMID 9324085
FT-0950	Lifshitz, M; 1997	Carbamate poisoning in early childhood and in adults.	Exclude; EX02_POPULATION	PMID 9022648

Report ID	Author / year	Title	Current disposition	Identifiers
FT-0951	Lau, G T; 1996	Sertraline overdose.	Exclude; EX02_POPULATION	PMID 8808373
FT-0958	Rosenberg, M L; 1989	The friction sweat test as a new method for detecting facial anhidrosis in patients with Horner's syndrome.	Core; IN01	PMID 2801865
FT-0959	van der Wiel, H L; 1988	The diagnosis of Horner's syndrome.	Exclude; EX07	PMID 3208466
FT-0960	Van der Wiel, H L; 1986	The diagnosis of Horner's syndrome. Use and limitations of the cocaine test.	Exclude; EX02	PMID 3723131
FT-0961	Fazio, D T; 1985	Acute angle-closure glaucoma associated with surgical anesthesia.	Exclude; EX04_NO_RELEVANT_PUPIL_DATA	PMID 3977709
FT-0963	Peden, N R; 1982	The problem of psilocybin mushroom abuse.	Exclude; EX02_POPULATION	PMID 7173927
FT-0964	Bawaskar, H S; 1982	Diagnostic cardiac premonitory signs and symptoms of red scorpion sting.	Exclude; EX02	PMID 6120403
FT-0967	Wadia, R S; 1974	Neurological manifestations of organophosphorous insecticide poisoning.	Exclude; EX07	PMID 4853328
FT-0969	Thompson, H S; 1971	The fixed dilated pupil. Sudden iridoplegia or mydriatic drops? A simple diagnostic test.	Exclude; EX07	PMID 5561379
FT-0971	Huong, Phan Thi Thu; 2026	Visual prognosis following open globe injury: clinical characteristics and risk factors from Vietnam National Eye Hospital.	Core; IN01	PMID 41572989
FT-0972	Bourquard, F; 2026	Diagnosis and management of orbital compartment syndrome caused by retrobulbar hematoma following orbital fractures.	Exclude; EX02_POPULATION	PMID 41529623
FT-0973	Reddy, Yeddula Venkata Rohith; 2025	Clinical profile of ocular trauma in a tertiary care hospital at Kolar district, South India.	Exclude; EX02_POPULATION	PMID 41466647
FT-0974	Ding, Yang; 2025	Intracranial aneurysm risk stratification in acute ocular motor nerve palsy based on clinical features.	Core; IN	PMID 41125709
FT-0975	Disse, Leah R; 2025	Differentiation of Horner Syndrome and Physiological Anisocoria by Automated Pupillometry.	Core; IN	PMID 40833793
FT-0976	Anant, Shruti; 2025	Demographic and Clinical Factors Associated with Mechanisms of Open Globe Injury in the United States: A Multi-Center Study.	Exclude; EX07	PMID 40357457
FT-0977	Karimaghaei, Sam; 2025	Ocular Hypertension Following Open Globe Injury in Patients Undergoing Pars Plana Vitrectomy.	Exclude; EX07	PMID 40276378

Report ID	Author / year	Title	Current disposition	Identifiers
FT-0978	Siebald, Franziska; 2025	Ultrasound pupillometry for the detection of a relative afferent pupillary defect (RAPD): Systematic evaluation in patients with optic neuritis and comparison with infrared video pupillometry.	Core; IN	PMID 39792831
FT-0979	Kitnarong, Naris; 2024	Incidence of acute angle closure in patients with primary angle closure without prophylactic iridotomy during pharmacological mydriasis for cataract surgery.	Exclude; EX04	PMID 39705431
FT-0980	Klyscz, Philipp; 2024	Application of the international criteria for optic neuritis in the Acute Optic Neuritis Network.	Core; IN01	PMID 39099240
FT-0981	Tabata, Shinya; 2024	Recovery of Oculomotor Nerve Palsy After Surgical and Endovascular Repair of Unruptured Internal Carotid-Posterior Communicating Artery Aneurysms.	Exclude; EX07	PMID 38796144
FT-0982	Shariati, Mehrdad Motamed; 2024	Development, comparison, and internal validation of prediction models to determine the visual prognosis of patients with open globe injuries using machine learning approaches.	Exclude; EX02_POPULATION	PMID 38773484
FT-0983	Ying, Yue; 2023	The occurrence of acute primary angle closure triggered, aggravated, and accelerated by COVID-19 infection: retrospective observational study.	Exclude; EX07	PMID 37645709
FT-0984	Liu, Xin; 2023	Clinical and imaging characteristics, outcomes and prognostic factors of intraocular foreign bodies extracted by vitrectomy.	Exclude; EX02_POPULATION	PMID 37644096
FT-0985	Gibbons, Alison B; 2024	Subperiosteal and Orbital Abscesses: A Comparison of Clinical Features, Management, and Outcomes.	Core; IN02	PMID 37581883
FT-0986	Porapaktham, Tuangprot; 2023	Open Globe Injury in a Tertiary Hospital of Northern Thailand: No Vision Survival and Ocular Trauma Score.	Exclude; EX02_POPULATION	PMID 36721667
FT-0987	Zhong, Eric; 2022	Orbital Fractures and Risk Factors for Ocular Injury.	Core; IN	PMID 36544896
FT-0988	Bruegger, Dominik; 2022	Evaluation of the Reddesa Chart, a New Red Desaturation Testing Method, for Optic Neuritis Screening and Grading in Clinical Routine.	Core; IN02	PMID 35873783
FT-0990	Donaldson, Laura; 2023	Yield of investigations in patients with questionable nonglaucomatous optic neuropathy.	Exclude; EX04	PMID 35123944
FT-0991	Singh, Ishan; 2022	Ocular Outcomes of Maxillofacial Trauma - A Decennary Descriptive Study.	Exclude; EX02_POPULATION	PMID 34967531
FT-0992	Gross, Andrew W; 2022	Non-traumatic open globe injuries: presenting characteristics and visual outcomes.	Core; IN02	PMID 34857923
FT-0993	Top Karti, Dilek; 2022	The evaluation of patient demographics, etiologies and apraclonidine test results in adult Horner's syndrome.	Core; IN02	PMID 34718919
FT-0994	Ng, Hui Ruan; 2021	The Epidemiological Profile of Open Globe Injuries and Prognostic Factors in a Tertiary Care Centre.	Exclude; EX07	PMID 34327079

Report ID	Author / year	Title	Current disposition	Identifiers
FT-0996	Choovuthayakorn, Janejit; 2020	Globe rupture: a single-center retrospective study of demographic patterns and outcomes.	Exclude; EX02_POPULATION	PMID 33154518
FT-0997	Erickson, B P; 2020	Evidence-based algorithm for the management of acute traumatic retrobulbar haemorrhage.	Exclude; EX07	PMID 32546417
FT-0998	Weerakoon, Sitara M; 2020	Investigation of Pupillary Changes After Carotid Endarterectomy and Carotid Stent Placement Using Automated Pupillometry.	Exclude; EX07	PMID 32107154
FT-1000	Bailey, Lucy A; 2019	Visual Outcomes of Patients With Retrobulbar Hemorrhage Undergoing Lateral Canthotomy and Cantholysis.	Exclude; EX07	PMID 31693632
FT-1001	Jiang, Hao; 2019	Analysis of Ocular Injury Characteristics in Survivors of the 8.12 Tianjin Port Explosion, China.	Core; IN	PMID 31485339
FT-1002	Siuko, Mika; 2019	The clinical spectrum and prognosis of idiopathic acute optic neuritis: A longitudinal study in Southern Finland.	Exclude; EX02_POPULATION	PMID 31401426
FT-1003	Gupta, Richa; 2019	Predicting visual outcome after open globe injury using classification and regression tree model: the Moradabad ocular trauma study.	Exclude; EX02_POPULATION	PMID 31358146
FT-1005	Kim, Hyeong Min; 2018	Quantitative analysis of pupillometry in isolated third nerve palsy.	Core; IN	PMID 30496292
FT-1006	Kitaguchi, Yoshiyuki; 2019	Bony Orbital Decompression Following Lateral Canthotomy and Cantholysis for Traumatic Orbital Compartment Syndrome.	Core; IN02	PMID 30444775
FT-1007	Chow, Jeffrey; 2018	Primary Assessment of the Patient With Orbital Fractures Should Include Pupillary Response and Visual Acuity Changes to Detect Occult Major Ocular Injuries.	Exclude; EX02_POPULATION	PMID 29782814
FT-1008	Johnson, Nigel R; 2018	Ophthalmological injuries associated with fractures of the orbitozygomaticomaxillary complex.	Exclude; EX07	PMID 29506872
FT-1009	Yoo, Yung Ju; 2017	Efficacy of digital pupillometry for diagnosis of Horner syndrome.	Exclude; EX02_POPULATION	PMID 28575101
FT-1010	Malik, Altaf Hussain; 2017	Ocular Injuries in Patients of Zygomatico-Complex (ZMC) Fractures.	Exclude; EX07	PMID 28439168
FT-1011	Sborgia, G; 2017	25-Gauge Vitrectomy in Open Eye Injury with Retained Foreign Body.	Core; IN01	PMID 28163929
FT-1013	Beebe, Johanna D; 2017	The Yield of Diagnostic Imaging in Patients with Isolated Horner Syndrome.	Exclude; EX07	PMID 27886891
FT-1016	Ho, Trung Q; 2017	The Incidence of Ocular Injuries in Isolated Orbital Fractures.	Exclude; EX02_POPULATION	PMID 26835822
FT-1017	Serdarevic, Raif; 2015	The ocular trauma score as a method for the prognostic assessment of visual acuity in patients with close eye injuries.	Exclude; EX02_POPULATION	PMID 26005272

Report ID	Author / year	Title	Current disposition	Identifiers
FT-1018	Chen, Ying; 2015	Evaluation and neuroimaging of the Horner syndrome.	Exclude; EX02_POPULATION	PMID 25863849
FT-1020	Al-Eajailat, Suha Mikail; 2014	The role of magnetic resonance imaging and visual evoked potential in management of optic neuritis.	Exclude; EX02_POPULATION	PMID 25018804
FT-1021	Sun, Michelle T; 2014	Traumatic orbital compartment syndrome: importance of the lateral canthomy and cantholysis.	Qualitative / linkage only; outside core	PMID 24810151
FT-1022	Yang, Xuejiao; 2013	Effect of anterior chamber paracentesis on initial treatment of acute angle closure.	Exclude; EX07	PMID 24314422
FT-1023	Agrawal, Rupesh; 2013	Pre-operative variables affecting final vision outcome with a critical review of ocular trauma classification for posterior open globe (zone III) injury.	Core; IN01	PMID 24212303
FT-1024	Pimolrat, Weeraya; 2014	Predictive factors of open globe injury in patients requiring vitrectomy.	Exclude; EX02_POPULATION	PMID 23830208
FT-1025	Feng, Kang; 2013	Risk factors, anatomical, and visual outcomes of injured eyes with proliferative vitreoretinopathy: eye injury vitrectomy study.	Exclude; EX02_POPULATION	PMID 23538583
FT-1026	Dehghani, Alireza; 2012	Ultrasonography in distinguishing optic neuritis from nonarteritic anterior ischemic optic neuropathy.	Core; IN02	PMID 23210062
FT-1028	Jovanovic, Milos; 2012	Ocular injuries caused by airsoft guns - ten-year experience.	Exclude; EX02_POPULATION	PMID 23654010
FT-1029	Choovuthayakorn, J; 2011	Predictive factors and outcomes of posterior segment intraocular foreign bodies.	Exclude; EX02_POPULATION	PMID 21921948
FT-1030	Rose, Nice; 2011	Acute optic neuritis following infection with chikungunya virus in southern rural India.	Qualitative / linkage only; outside core	PMID 21131222
FT-1031	Almog, Yehoshua; 2010	Diagnostic value of imaging in horner syndrome in adults.	Core; IN01	PMID 20182199
FT-1032	Hyun Lee, Seung; 2010	Emergent risk factors associated with eyeball loss and ambulatory vision loss after globe injuries.	Exclude; EX02_POPULATION	PMID 20068482
FT-1033	Heidari, Ebadollah; 2010	Surgical treatment of severely traumatized eyes with no light perception.	Qualitative / linkage only; outside core	PMID 19952999
FT-1035	Bhattacharjee, Harsha; 2008	Indirect optic nerve injury in two-wheeler riders in northeast India.	Core; IN	PMID 18974518
FT-1037	Akagi, Tadamichi; 2008	Cause and prognosis of neurologically isolated third, fourth, or sixth cranial nerve dysfunction in cases of oculomotor palsy.	Core; IN	PMID 18369697

Report ID	Author / year	Title	Current disposition	Identifiers
FT-1038	Arnold, Marcel; 2008	Ultrasound diagnosis of spontaneous carotid dissection with isolated Horner syndrome.	Exclude; EX07	PMID 18006862
FT-1039	Kim, Sang Hyun; 2007	Cranial nerve palsies accompanying pituitary tumour.	Core; IN01	PMID 17964787
FT-1040	Kent, Jerrod S; 2007	Indoor soccer-related eye injuries: should eye protection be mandatory?	Exclude; EX02_POPULATION	PMID 17641705
FT-1042	Schmidt, G W; 2008	Vision survival after open globe injury predicted by classification and regression tree analysis.	Core; IN	PMID 17588667 DOI 10.1016/j.ophtha.2007.04.008
FT-1043	He, Dongmei; 2007	Association between ocular injuries and internal orbital fractures.	Exclude; EX02_POPULATION	PMID 17368368
FT-1044	Mwanza, Jean-Claude; 2006	Ocular motor nerve palsy: a clinical and etiological study.	Exclude; EX02_POPULATION	PMID 16921214
FT-1045	Tsuda, Hiromasa; 2006	Abducens nerve palsy and postganglionic Horner syndrome with or without severe headache.	Qualitative / linkage only; outside core	PMID 16908941
FT-1046	Wickham, Louisa; 2006	Outcomes of surgery for posterior segment intraocular foreign bodies--a retrospective review of 17 years of clinical experience.	Exclude; EX07	PMID 16788826
FT-1047	Singh, Deependra Vikram; 2005	Visual outcome after fireworks injuries.	Exclude; EX02_POPULATION	PMID 16096549
FT-1050	Globocnik Petrovic, Mojca; 2004	Prognostic factors in open eye injury managed with vitrectomy: retrospective study.	Exclude; EX02_POPULATION	PMID 15185422
FT-1051	Hooi, S H; 2003	Open-globe injuries: the experience at Hospital Sultanah Aminah, Johor Bahru.	Exclude; EX02_POPULATION	PMID 14750381
FT-1053	Rosenkranz, Michael; 2003	Horner syndrome related to ipsilateral carotid wall hematoma after stent placement for the treatment of carotid stenoses.	Core; IN01	PMID 13679260
FT-1054	Pieramici, Dante J; 2003	The prognostic significance of a system for classifying mechanical injuries of the eye (globe) in open-globe injuries.	Exclude; EX07	PMID 12707539
FT-1058	Sobaci, G; 2000	Deadly weapon-related open-globe injuries: outcome assessment by the ocular trauma classification system.	Core; IN01	PMID 10653412
FT-1059	Wakakura, M; 1999	Baseline features of idiopathic optic neuritis as determined by a multicenter treatment trial in Japan. Optic Neuritis Treatment Trial Multicenter Cooperative Research Group (ONMRG).	Core; IN02	PMID 10340795
FT-1063	Jacobson, D M; 1998	Pupil involvement in patients with diabetes-associated oculomotor nerve palsy.	Core; IN	PMID 9639439

Report ID	Author / year	Title	Current disposition	Identifiers
FT-1067	Karma, A; 1995	Diagnosis and clinical characteristics of ocular Lyme borreliosis.	Exclude; EX04_NO_RELEVANT_PUPIL_DATA	PMID 7832219
FT-1069	Jacobson, D M; 1994	A prospective evaluation of cholinergic supersensitivity of the iris sphincter in patients with oculomotor nerve palsies.	Core; IN	PMID 8085595
FT-1071	Hershenfeld, S A; 1993	Monocular temporal hemianopia.	Qualitative / linkage only; outside core	PMID 8343470
FT-1072	Kardon, R H; 1993	The relationship between static perimetry and the relative afferent pupillary defect.	Exclude; EX02_POPULATION	PMID 8442495
FT-1073	Glazer, L C; 1993	Choroidal rupture and optic atrophy.	Exclude; EX04	PMID 8435396
FT-1075	Simcock, P R; 1992	Neurological problems presenting to an ophthalmic casualty department.	Exclude; EX07	PMID 1488876
FT-1077	Digre, K B; 1992	Selective MR imaging approach for evaluation of patients with Horner's syndrome.	Exclude; EX02_POPULATION	PMID 1595449
FT-1079	Pearson, R V; 1991	Anisocoria in unilateral ophthalmic disease.	Core; IN	PMID 1991083
FT-1081	Cremer, S A; 1990	Hydroxyamphetamine mydriasis in Horner's syndrome.	Exclude; EX07	PMID 2368824
FT-1083	Brazier, D J; 1989	Iris autonomic function in acute glaucoma.	Exclude; EX07	PMID 2612673
FT-1084	Bartleson, J D; 1986	Minimal oculomotor nerve paresis secondary to unruptured intracranial aneurysm.	Core; IN01	PMID 3753262
FT-1087	Van der Wiel, H L; 1983	Localization of Horner's syndrome. Use and limitations of the hydroxyamphetamine test.	Exclude; EX02	PMID 6854351
FT-1088	van der Wiel, H L; 1982	Horner's syndrome. Criteria for oculosympathetic denervation.	Core; IN	PMID 7175553
FT-1089	Cox, T A; 1982	Visual evoked potential and pupillary signs. A comparison in optic nerve disease.	Exclude; EX04	PMID 7138329
FT-1090	Maloney, W F; 1980	Evaluation of the causes and accuracy of pharmacologic localization in Horner's syndrome.	Exclude; EX02_POPULATION	PMID 7425056
FT-1092	Ellis, C J; 1979	The afferent pupillary defect in acute optic neuritis.	Exclude; EX02	PMID 501365
FT-1093	Trobe, J D; 1978	Meningiomas and aneurysms of the cavernous sinus. Neuro-ophthalmologic features.	Qualitative / linkage only; outside core	PMID 629685
FT-1094	O'DOHERTY, D S; 1958	Diagnostic value of Horner's syndrome in thrombosis of the carotid artery.	Exclude; EX07	PMID 13590396
FT-1100	Nyholm, Benjamin; 2022	Superior reproducibility and repeatability in automated quantitative pupillometry compared to standard manual assessment, and quantitative pupillary response parameters present high reliability in critically ill cardiac patients.	Exclude; EX07	PMID 35901103

Report ID	Author / year	Title	Current disposition	Identifiers
FT-1101	Singer, Kathleen E; 2021	Efficacy of Noninvasive Technologies in Triaging Traumatic Brain Injury and Correlating With Intracranial Pressure: A Prospective Study.	Exclude; EX07	PMID 33540153
FT-1102	Yokobori, Shoji; 2018	Quantitative pupillometry and neuron-specific enolase independently predict return of spontaneous circulation following cardiogenic out-of-hospital cardiac arrest: a prospective pilot study.	Exclude; EX07	PMID 30374189 DOI 10.1038/s41598-018-34367-x
FT-1103	Martin, Sean K; 2002	Blunt trauma patients with prehospital pulseless electrical activity (PEA): poor ending assured.	Exclude; EX02_POPULATION	PMID 12435937
FT-1105	McGrath, Lynn B.; 2022	Mobile Smartphone-Based Digital Pupillometry Curves in the Diagnosis of Traumatic Brain Injury	Exclude; EX07	PMID 35844221 DOI 10.3389/fnins.2022.893711
FT-1107	Okamoto, Saiko; 2022	Use of Automated Infrared Pupillometry to Predict Delirium in the Intensive Care Unit: A Prospective Observational Study	Core; IN	PMID 36081493 DOI 10.1177/23779608221124417
FT-1108	Jolkovsky, Eliana L.; 2022	Impact of acute intoxication on quantitative pupillometry assessment in the emergency department	Core; IN	PMID 36311337 DOI 10.1002/emp2.12825
FT-1114	Not parsed; 2020	Added value of somato-sensory evoked potentials amplitude for prognostication after cardiac arrest.	Exclude; EX07	PMID 32044334 DOI 10.1016/j.resuscitation.2020.01.025
FT-1115	Not parsed; 2021	Are neurophysiologic tests reliable, ultra-early prognostic indices after cardiac arrest?	Core; IN01	PMID 33573889 DOI 10.1016/j.neucli.2021.01.005
FT-1116	Not parsed; 2025	Association of prehospital pupillary diameter with return of spontaneous circulation and neurological outcome after out-of-hospital cardiac arrest: A multicenter retrospective analysis.	Core; IN	PMID 41142217 DOI 10.1016/j.resplu.2025.101112
FT-1117	Not parsed; 2024	Automated Pupillometry Is Able to Discriminate Patients with Acute Stroke from Healthy Subjects: An Observational, Cross-Sectional Study.	Exclude; EX07	PMID 38928617 DOI 10.3390/brainsci14060616
FT-1119	Not parsed; 2021	Changes in autonomic tone during delirium in acute stroke patients assessed by pupillometry and skin conductance.	Core; IN	PMID 34284172
FT-1122	Not parsed; 2023	Cognitive impairment in diffuse axonal injury patients with favorable outcome.	Core; IN01	PMID 36761409 DOI 10.3389/fnins.2023.1077858
FT-1124	Not parsed; 2007	Correlation of a high D-dimer level with poor outcome in traumatic intracranial hemorrhage.	Exclude; EX07	PMID 17880559 DOI 10.1111/j.1468-1331.2007.01908.x
FT-1126	Not parsed; 2024	Covert Consciousness in Acute Brain Injury Revealed by Automated Pupillometry and Cognitive Paradigms.	Core; IN	PMID 38605221

Report ID	Author / year	Title	Current disposition	Identifiers
FT-1131	Not parsed; 2022	Dexmedetomidine and Other Analgosedatives Alter Pupil Characteristics in Critically Ill Patients.	Exclude; EX07	PMID 35783547 DOI 10.1097/cce.0000000000000691
FT-1132	Not parsed; 2023	Diurnal variation in quantitative pupillary reactivity in large hemispheric stroke	Exclude; EX07	DOI 10.18700/jnc.230030
FT-1135	Not parsed; 2022	Early risk stratification for progression to death by neurological criteria following out-of-hospital cardiac arrest.	Exclude; EX07	PMID 35914657 DOI 10.1016/j.resuscitation.2022.07.029
FT-1136	Not parsed; 2024	Early Tracheostomy versus Late Tracheostomy in Patients with Moderate-to-Severe Traumatic Brain Injury.	Core; IN01	PMID 39205886 DOI 10.1055/s-0044-1787794
FT-1142	Not parsed; 2021	Findings Predictive of Poor Outcome in Grade 5 Subarachnoid Hemorrhage: A Cohort Study.	Exclude; EX07	PMID 33472716 DOI 10.1017/cjn.2021.13
FT-1144	Al-Obaidi, Sameer Z.; 2019	Impact of Increased Intracranial Pressure on Pupillometry: A Replication Study	Exclude; EX07	PMID 32166235 DOI 10.1097/CCE.0000000000000054
FT-1145	Not parsed; 2025	Importance of Preoperative Pupillary Reflex in Traumatic Optic Neuropathy.	Core; IN02	PMID 39136043 DOI 10.3340/jkns.2024.0083
FT-1147	Nyholm, B.; 2023	Influence of blood-pressure and oxygen levels on the prognostic value of quantitative pupillometry for comatose cardiac arrest patients - a BOX-trial substudy	Exclude; EX_CONFERENCE_INSUFFICIENT_DATA	DOI 10.1093/ehjacc/zuad036.142
FT-1149	Not parsed; 2018	Influence of Strategic Cortical Infarctions on Pupillary Function	Core; IN	DOI 10.3389/fneur.2018.00916
FT-1150	Not parsed; 2020	Initial pupil status is a strong predictor for in-hospital mortality after aneurysmal subarachnoid hemorrhage	Exclude; EX07	PMID 32179801 DOI 10.1038/s41598-020-61513-1
FT-1151	Not parsed; 2023	Mortality Prediction in Severe Traumatic Brain Injury Using Traditional and Machine Learning Algorithms.	Exclude; EX07	PMID 37062757 DOI 10.1089/neu.2022.0221
FT-1155	Not parsed; 2013	Predicting outcome after cardiopulmonary arrest in therapeutic hypothermia patients: clinical, electrophysiological and imaging prognosticators.	Core; IN	PMID 23809738
FT-1156	Not parsed; 2021	Predicting the Health-related Quality of Life in Patients Following Traumatic Brain Injury.	Core; IN01	PMID 34159258 DOI 10.1055/s-0041-1726426
FT-1157	Not parsed; 2018	Prediction of neurological outcomes following the return of spontaneous circulation in patients with out-of-hospital cardiac arrest: Retrospective fast-and-frugal tree analysis.	Core; IN01	PMID 30292802 DOI 10.1016/j.resuscitation.2018.10.002
FT-1160	Steiner, Thorsten; 1997	Prognosis of Stroke Patients Requiring Mechanical Ventilation in a Neurological Critical Care Unit	Exclude; EX07	PMID 9099184 DOI 10.1161/01.STR.28.4.711
FT-1161	Not parsed; 2024	Prognostic factors and clinical nomogram for in-hospital mortality in traumatic brain injury.	Exclude; EX02_POPULATION	PMID 38176118 DOI 10.1016/j.ajem.2023.12.037

Report ID	Author / year	Title	Current disposition	Identifiers
FT-1163	Not parsed; 2020	Prognostic role of EEG identical bursts in patients after cardiac arrest: Multimodal correlation.	Exclude; EX07	PMID 32004660 DOI 10.1016/j.resuscitation.2020.01.017
FT-1165	Not parsed; 2021	PROLOGUE (PROgnostication using LOGistic regression model for Unselected adult cardiac arrest patients in the Early stages): Development and validation of a scoring system for early prognostication in unselected adult cardiac arrest patients.	Core; IN01	PMID 33388366 DOI 10.1016/j.resuscitation.2020.12.022
FT-1166	Not parsed; 2024	Pupillary dilation velocity is reduced in intensive care unit patients with septic shock.	Core; IN	PMID 37722925 DOI 10.1111/aas.14327
FT-1168	Not parsed; 2023	Pupillary light reflex measured with quantitative pupillometry has low sensitivity and high specificity for predicting neuroworsening after traumatic brain injury.	Exclude; EX07	PMID 36763466
FT-1169	Not parsed; 2024	Pupillometer-Based Neurological Pupil Index Differential: A Potential Predictor of Post-Stroke Delirium	Exclude; EX07	PMID 38836279 DOI 10.5853/jos.2023.04406
FT-1170	Not parsed; 2023	Quantitative analysis of early apparent diffusion coefficient values from MRIs for predicting neurological prognosis in survivors of out-of-hospital cardiac arrest: an observational study.	Core; IN	PMID 37880777 DOI 10.1186/s13054-023-04696-z
FT-1174	Nyholm, B.; 2023	Quantitative pupillometry thresholds for early neuroprognostication after cardiac arrest: A BOX-trial substudy	Qualitative / linkage only; outside core	DOI 10.1093/ehjacc/zuad036.141
FT-1178	Not parsed; 2011	Risk factors analysis on traumatic brain injury prognosis.	Exclude; EX02_POPULATION	PMID 21703117 DOI 10.1016/s1001-9294(11)60027-8
FT-1179	Not parsed; 2018	Risk factors predicting prognosis and outcome of elderly patients with isolated traumatic brain injury.	Core; IN01	PMID 30390698 DOI 10.1186/s13018-018-0975-y
FT-1183	Not parsed; 2011	Single photon emission computed tomography scanning: A predictor of outcome in vegetative state of head injury.	Exclude; EX02_POPULATION	PMID 21716866 DOI 10.4103/0976-3147.80079
FT-1185	Not parsed; 2021	Supratentorial intracerebral hemorrhage volume and other CT variables predict the neurological pupil index.	Exclude; EX07	PMID 33341651
FT-1189	Not parsed; 2016	The outcomes of early aneurysm repair in World Federation of Neurosurgical Societies grade V subarachnoid haemorrhage patients with emphasis on those presenting with a Glasgow Coma Scale score of 3.	Exclude; EX07	PMID 27450281 DOI 10.1016/j.jocn.2016.03.035
FT-1194	Not parsed; 2018	Validation of Visual Objective Biomarkers for Acute Concussion.	Core; IN	PMID 29635572 DOI 10.1093/milmed/usx166
FT-1200	Not parsed; 2021	Assessing the Clinical Features and Blood Biochemistries of Acute Organophosphorus Chemical Warfare Agents in Iranian Veterans	Exclude; EX07	DOI 10.32598/ijmtfm.v11i2.32857

Report ID	Author / year	Title	Current disposition	Identifiers
FT-1213	Kalwaghe, Aditya P.; 2021	Retrospective Study of Organophosphorus Poisoning Patients with Reference to Clinical Presentation, Serum Cholinesterase Level and Outcome to Treatment	Exclude; EX02_POPULATION	DOI 10.21474/IJAR01/13574
FT-1221	Not parsed; 2017	Pseudo cholinesterase-diagnostic and prognostic value in organophosphorus poisoning	Core; IN01	DOI 10.18203/2320-6012.ijrms20172976
FT-1223	Meeker, Michele; 2005	Pupil Examination: Validity and Clinical Utility of an Automated Pupillometer	Exclude; EX02_POPULATION	PMID 15794443
FT-1227	Not parsed; 2025	Pupillometry changes following ocular administration of brimonidine in patients experiencing acute migraine attack	Core; IN	PMID 40660595 DOI 10.1097/md.0000000000043292
UF-312A3241	Cruz, Julio; 1998	The first decade of continuous monitoring of jugular bulb oxyhemoglobin saturation: Management strategies and clinical outcome	Exclude; EX07	PMID 9468174 DOI 10.1097/00003246-199802000-00039
UF-43BA3EE5	Whipple, Julianne K.; 1994	Difficulties in Diagnosing Narcotic Overdoses in Hospitalized Patients	Exclude; EX02	PMID 8038463 DOI 10.1177/106002809402800403
UF-46842A7B	Bouzarth WF; 1978	Head Injury Watch Sheet Modified for a Digital Scale	Exclude; EX07	DOI 10.1097/00005373-197808000-00003
UF-53E7DD52	Mansouri, Mohammad Reza; 2016	Ocular trauma treated with pars plana vitrectomy: early outcome report	Core; IN01	PMID 27275432 DOI 10.18240/ijo.2016.05.18
UF-A019E28A	Drake, C. G.; 1962	Hypothermia in the Treatment of Critical Head Injury	Duplicate alias; not counted	PMID 20327281
UF-B77A406A	Kühne, C. A.; 2013	Dringlichkeit neurochirurgischer Interventionen bei schwerem Schädel-Hirn-Trauma	Core; IN01	DOI 10.1007/s00113-011-2042-6
UF-C8DA9BFA	Schneider, Th.; 1989	Verlaufsbeobachtung computertomographischer Befunde bei traumatischen intrazerebralen Hämatomen	Exclude; EX02_POPULATION	
UF-DB97C8E3	Kawaguchi, Masahiko; 1996	Preoperative Predictors of Reduction in Arterial Blood Pressure Following Dural Opening During Surgical Evacuation of Acute Subdural Hematoma	Exclude; EX07	PMID 8829557 DOI 10.1097/00008506-199604000-00003
