# Supplementary material for: An Evidence Map of Pupillary Assessment in a Preselected Acute-Care Literature Archive
Source: Diagnostics (Basel). 2026 Nov 9;16(21):3698. doi: 10.3390/diagnostics16213698 (PMC13761962; doi:10.3390/diagnostics16213698)
Supplement: Supplementary file 1 [file diagnostics-16-03698-s001.zip › 05_Reporting_Checklists.pdf]

Reporting checklists and applicability crosswalk

These 80 entries describe the methods actually performed. Locations refer to physical pages of the 19-page revised manuscript and 44-page S1-S9 supplement. Historical independent review, three-person consensus, later human verification and technical assistance are distinguished; search and source limitations are not presented as completed procedures.

PRISMA ScR 22 items

Item	Topic	Status and explanation	Verified location
1	Title	Identified as a preselected-archive evidence map, not labelled a comprehensive scoping review; this is a design-specific limitation of the checklist title item.	Title, main p. 1; Section 2.1, main pp. 2-3; S1, supplement p. 2
2	Structured abstract	The structured abstract prioritizes the observed 237/355 unestablished acquisition labels, separating 38 unresolved after sampled reconciliation from 199 not sampled, and limits clinical-group comparisons accordingly. The conditional 53.3% scenario remains in the main text, not the abstract.	Abstract, main p. 1
3	Rationale	Reported; heterogeneity of clinical purposes and bounded archive rationale.	Introduction, main pp. 1-2; S1, supplement p. 2
4	Objectives	Reported; six descriptive mapping domains and no pooled-performance objective.	Introduction, main pp. 1-2; S1, supplement p. 2
5	Protocol and registration	PROSPERO CRD420261441918 Version 1.0 published 5 July 2026 is verified. Later operational rules and their dates are contrasted with registration in S9. The supplied PDF does not establish a later amendment.	Section 2.1, main pp. 2-3; S1, supplement p. 2; S9, supplement pp. 32-44
6	Eligibility	Reported: numerical adult rule, acuity, original pupil evidence, qualitative exceptions and report-specific post hoc retrieval closure.	Section 2.3, main pp. 3-4; S1, supplement p. 2; S3, supplement pp. 8-11; S5, supplement pp. 13-14
7	Information sources	Partial historical reconstruction. Exact available source logs and dates supplied; incomplete out-of-archive integration explicitly retained.	Section 2.2, main p. 3; S2, supplement pp. 3-8
8	Search	Verbatim historical search preserved; a separately dated 28 October syntax audit with July publication cutoff adds 41 plus two unintegrated candidates. Known-article check identifies a missed methodological report. Comprehensive retrieval is not claimed.	Section 2.2, main p. 3; S2, supplement pp. 3-8

Item	Topic	Status and explanation	Verified location
9	Selection of sources	Chiao-Yin Cheng and Hua Ho independently performed original screening, extraction and mapping, with three-person consensus involving Cheng-Yueh Li. Cheng prepared and passed the first audit form to Jen-Tang Sun. The 43/50 result is Sun's human-confirmed first pass before reconciliation. Sun had not made the original decisions; masking to earlier categories and reasons is not documented. Preparation, human judgments and later reconciliation are distinguished.	Section 2.4, main pp. 4-5; Section 3.5, main p. 12; S9, supplement p. 32; S9b, supplement p. 36; S9d-e, supplement pp. 38-43
10	Data charting	Historical charting and subsequent source reconciliation remain traceable. The pre-reconciliation age result was 43/50, not 50/50; seven discrepancies were later resolved. The 239-record eligibility exercise used 21 targeted PDF rechecks and 218 preserved-evidence checks and was not a complete acquisition re-audit.	Section 2.4, main pp. 4-5; Section 3.5, main p. 12; S9, supplement p. 32; S9b, supplement p. 36; S9d-e, supplement pp. 38-43
11	Data items	Six domains, age evidence, sources, denominators and restrictions defined. Funding of included reports was not systematically extracted.	Section 2.5, main pp. 5-6; S3, supplement pp. 8-11; S8, supplement pp. 29-31; S9, supplement pp. 32-44
12	Critical appraisal methods	Not undertaken map-wide. No formal appraisal tool or new reviewer appraisal is claimed; optional item is explicitly not applicable to performed analysis.	Section 2.6, main p. 6; S9, supplement pp. 32-44
13	Synthesis methods	Core report counts, cross-tabulations and nonexclusive components are reported. The core-plus-EX07 scenario contains 629 records. Current 237 unestablished-acquisition labels are split into 38 sampled/reconciled and 199 unsampled; a separate conditional verification-yield scenario does not reclassify records.	Section 2.6, main p. 6; Section 3.4, main pp. 10-12; Section 3.5, main p. 12; S7b-b2, supplement pp. 25-26; S9f, supplement p. 44
14	Selection results	Current closed-archive flow reported. A complete original database-identification-to-inclusion pathway cannot be reconstructed.	Section 3.1, main pp. 6-7; S2, supplement pp. 3-8; S4, supplement p. 12; S5, supplement pp. 13-14
15	Source characteristics	Tables 1-3 are editable report distributions. All 871 IDs are indexed; 14 descriptive series and one linkage-only abstract are distinguished outside the core.	Section 3.2, main pp. 7-9; Section 3.3, main pp. 9-10; S4, supplement p. 12; S6, supplement pp. 15-18; S7, supplement pp. 19-28
16	Critical appraisal results	Not available because map-wide formal appraisal was not performed. Not presented as low risk or evidence of quality.	Section 2.6, main p. 6; S9, supplement pp. 32-44
17	Individual source results	Report-specific mapping and source-supported restrictions supplied. Complete effect estimates for all outcomes were not the scope of this descriptive map.	S4, supplement p. 12; S5, supplement pp. 13-14; S6, supplement pp. 15-18; S8, supplement pp. 29-31

Item	Topic	Status and explanation	Verified location
18	Synthesis results	Core and 629-record combined aetiology-by-acquisition matrices, six-domain age-rule sensitivity, raw 43/50 age confirmation and acquisition-verification coverage are reported. The conditional unresolved share is 53.3% (Wilson-derived scenario range 45.8-58.7%), not a confidence interval for all 355 reports.	Section 2.6, main p. 6; Section 3.4, main pp. 10-12; Section 3.5, main p. 12; S7b-b2, supplement pp. 25-26; S9f, supplement p. 44
19	Summary of evidence	Clinical measurement, diagnostic, prognostic and management implications separated and bounded to the archive.	Discussion, main pp. 13-15; S8, supplement pp. 29-31
20	Limitations	Closed archive, incomplete search integration, numeric-age rule, source closures, unequal acquisition verification, overlap and absent formal appraisal are reported. The 199 unsampled acquisition labels may include recoverable classifications.	Section 4.5, main p. 15; S9, supplement pp. 32-44
21	Conclusions	Bounded descriptive interpretation; no comparative-superiority or field-wide completeness claim.	Conclusions, main pp. 15-16
22	Funding	Present-study funding declaration reported. Funding of individual included reports was not systematically extracted and is unavailable.	Funding, main p. 16; S9, supplement pp. 32-44

PRISMA S 16 items

Item	Topic	Status and explanation	Verified location
1	Databases and platforms	PubMed/Entrez execution documented; available supplementary-source labels and provenance listed. Full all-source execution is not asserted.	Section 2.2, main p. 3; S2, supplement pp. 3-8
2	Multi-database searching	No single multi-database platform run inferred. Overlapping source appearances and identifier groups kept distinct.	S2, supplement pp. 3-8
3	Registries	Historical planned registry sources retained. NCT and OSF source checks are targeted checks of already identified reports, not a new registry-identification search.	S2, supplement pp. 3-8; S5, supplement pp. 13-14
4	Other resources	Recovered source-specific inventory and completion limitations reported, including proxy-labelled provenance.	S2, supplement pp. 3-8
5	Citation searching	Original seeds and exhaustive backward/forward execution could not be fully reconstructed; no completed field-wide citation-search claim.	S2, supplement pp. 3-8
6	Contacts	No new investigator, expert or manufacturer contact was performed for this synchronization; historical contacts are not established by these files.	S9, supplement pp. 32-44
7	Additional methods	Local archive reconciliation, manual/UF additions and targeted main-source verification separated from new study identification.	S1, supplement p. 2; S2, supplement pp. 3-8; S5, supplement pp. 13-14
8	Full strategies	Original logged expressions remain intact. Canonical-heading corrections and free-text expansion are separately documented with raw current-date responses and complete IDs.	S2, supplement pp. 3-8
9	Limits and restrictions	Original July PubMed search had no listed date/language/age/human/publication-type filter. The October syntax comparison separately used a publication-date cutoff of 17 July 2026; eligibility age is not a search filter.	Section 2.2, main p. 3; S2, supplement pp. 3-8; S3, supplement pp. 8-11
10	Search filters	No validated published search-filter application documented. Custom concept modules are not labelled validated filters.	S2, supplement pp. 3-8
11	Prior work	v1.1 modular rerun and v1.0 audit history retained; no unsupported attribution to a published external strategy.	S2, supplement pp. 3-8

Item	Topic	Status and explanation	Verified location
12	Updates	Logged run 5 July, archive date 7 July and investigator-reported last search 17 July are distinct. The 28 October syntax audit is not a completed field-wide eligibility update.	Section 2.2, main p. 3; S1, supplement p. 2; S2, supplement pp. 3-8
13	Dates	Historical available dates and the current audit date are stated separately; current indexing results are not substituted for July counts.	S2, supplement pp. 3-8
14	Peer review of search	Formal independent search-strategy peer review was not documented. The technical syntax audit and independent article screening do not establish that peer review occurred.	S2, supplement pp. 3-8; S9, supplement pp. 32-44
15	Total records	Historical 8953 appearances/7440 PMIDs/1513 repeats remain unchanged. Current syntax comparison unions 7493/7534/7536 and +41/+2 deltas are explicitly separate.	Section 2.2, main p. 3; S2, supplement pp. 3-8
16	Deduplication	Original raw review/merge records retained; module union, historical merge events, source-group normalization and current one-alias removal distinguished.	S2, supplement pp. 3-8; S4, supplement p. 12

PRISMA 2020 applicability 42 items

Item	Topic	Status and explanation	Verified location
1	Title	Not a newly completed systematic review; retain the accurate evidence-map title.	Title, main p. 1; S1, supplement p. 2
2	Abstract	Structured descriptive abstract provided. Observed acquisition counts take priority; the conditional projection is retained only in the main text and supplement. No pooled estimate is reported.	Abstract, main p. 1
3	Rationale	Reported.	Introduction, main pp. 1-2
4	Objectives	Reported.	Introduction, main pp. 1-2
5	Eligibility	Reported including later operational rules and synthesis restrictions.	Section 2.3, main pp. 3-4; S1, supplement p. 2; S3, supplement pp. 8-11
6	Information sources	Partial historical reconstruction and incomplete integration explicitly reported.	Section 2.2, main p. 3; S2, supplement pp. 3-8
7	Search strategies	Logged PubMed expressions and original strategy materials supplied; all-source execution not fully recoverable.	S2, supplement pp. 3-8
8	Selection process	Chiao-Yin Cheng and Hua Ho independently performed original screening, extraction and mapping, with three-person consensus involving Cheng-Yueh Li. Cheng prepared and passed the first audit form to Jen-Tang Sun. The 43/50 result is Sun's human-confirmed first pass before reconciliation. Sun had not made the original decisions; masking to earlier categories and reasons is not documented. Preparation, human judgments and later reconciliation are distinguished.	Section 2.4, main pp. 4-5; Section 3.5, main p. 12; S9, supplement p. 32; S9b, supplement p. 36; S9d-e, supplement pp. 38-43
9	Data collection	Chiao-Yin Cheng prepared and passed the audit form to Jen-Tang Sun, who made source-based judgments; 43/50 reflects his confirmed first pass before reconciliation. Sun also confirmed discrepancy resolutions and 239 eligibility dispositions. Claude assisted provisional form preparation; Codex assisted source comparisons and technical checks. Human judgments were decisive; masking is not established.	Section 2.4, main pp. 4-5; Section 3.5, main p. 12; S9, supplement p. 32; S9b, supplement p. 36; S9d-e, supplement pp. 38-43
10a	Outcomes	Primary pupil-related endpoint, not every source outcome, defined.	Section 2.5, main pp. 5-6; S3, supplement pp. 8-11
10b	Other data items	Canonical/raw variables and missingness defined; included-report funding not systematically collected.	S3, supplement pp. 8-11; S9, supplement pp. 32-44

Item	Topic	Status and explanation	Verified location
11	Risk of bias methods	Not undertaken map-wide; no tool or reviewer assessment invented.	Section 2.6, main p. 6; S9, supplement pp. 32-44
12	Effect measures	Report counts and percentages only; no synthesized association or accuracy effect.	Section 2.6, main p. 6; S7, supplement pp. 19-28
13a	Synthesis eligibility	355 core reports versus 14 descriptive series plus one linkage-only abstract; EX07 sensitivity does not change eligibility.	Section 2.3, main pp. 3-4; Section 2.6, main p. 6; S8, supplement pp. 29-31
13b	Data preparation	Canonical normalization, distinct denominator units and no imputation of unestablished methods.	Section 2.5, main pp. 5-6; S3, supplement pp. 8-11
13c	Tables and displays	Three tables, three figures, full descriptive counts and report ledger supplied.	Results, main pp. 6-12; S4, supplement p. 12; S7, supplement pp. 19-28
13d	Synthesis methods	Exact integer counts and descriptive cross-tabulations; no meta-analysis or heterogeneity model.	Section 2.6, main p. 6
13e	Heterogeneity exploration	No inferential heterogeneity analysis; descriptive domain differences only.	Section 2.6, main p. 6; S7, supplement pp. 19-28
13f	Sensitivity analysis	The age-rule scenario adds 274 EX07 records to 355 core reports (629 total), without changing eligibility. A separate conditional scenario applies q=38/50 unresolved after reconciliation to 199 unsampled reports, holding the 38 sampled unresolved reports fixed; it assumes comparable verification yield.	Section 2.6, main p. 6; Section 3.4, main pp. 10-12; Section 3.5, main p. 12; S7b-b2, supplement pp. 25-26; S9f, supplement p. 44
14	Reporting-bias methods	Not assessed quantitatively; closed-frame and missing-source selection limitations described.	Section 4.5, main p. 15; S9, supplement pp. 32-44
15	Certainty methods	Not assessed; distribution mapping is not certainty grading.	Section 2.6, main p. 6; S9, supplement pp. 32-44
16a	Selection results	Closed-archive flow complete for 871 records, original field-wide identification lineage incomplete.	Section 3.1, main pp. 6-7; S2, supplement pp. 3-8; S4, supplement p. 12
16b	Excluded reports	All record IDs, source-supported reasons and source closures retained separately.	S4, supplement p. 12; S5, supplement pp. 13-14
17	Study characteristics	Report characteristics available; no independent-cohort assumption.	Section 3.2, main pp. 7-9; Section 3.3, main pp. 9-10; S4, supplement p. 12; S6, supplement pp. 15-18; S7, supplement pp. 19-28
18	Risk of bias results	Not available; map-wide formal assessment not performed.	S9, supplement pp. 32-44

Item	Topic	Status and explanation	Verified location
19	Individual results	Mapped variables and restricted uses supplied. All-outcome effect estimates are outside this descriptive analysis.	S4, supplement p. 12; S6, supplement pp. 15-18; S8, supplement pp. 29-31
20a	Synthesis characteristics	Core distributions shown; no formal risk-of-bias summary.	Results, main pp. 6-12; S7, supplement pp. 19-28; S9, supplement pp. 32-44
20b	Statistical syntheses	No meta-analysis or pooled-effect interval. Wilson intervals describe targeted audit proportions. The transformed 45.8-58.7% acquisition range is a conditional scenario band, not a confidence interval for the entire core.	Section 2.6, main p. 6; Section 3.4, main pp. 10-12; Section 3.5, main p. 12; S7b-b2, supplement pp. 25-26; S9f, supplement p. 44
20c	Heterogeneity results	No inferential heterogeneity investigation performed.	Section 2.6, main p. 6
20d	Sensitivity results	Six-domain age-rule sensitivity and the complete 629-record matrix are provided. Unestablished-acquisition labels are split by verification coverage; the conditional unresolved share would be 53.3% (45.8-58.7%) without changing the observed 237-record ledger total.	Section 2.6, main p. 6; Section 3.4, main pp. 10-12; Section 3.5, main p. 12; S7b-b2, supplement pp. 25-26; S9f, supplement p. 44
21	Reporting biases	Not formally assessed; selection limitations explicit.	Section 4.5, main p. 15; S9, supplement pp. 32-44
22	Certainty	No certainty rating assigned.	S9, supplement pp. 32-44
23a	Interpretation	Archive-bounded clinical interpretation provided.	Discussion, main pp. 13-15
23b	Evidence limitations	Age, methods, denominators, overlap and appraisal limits explicit.	Section 4.5, main p. 15; S8, supplement pp. 29-31; S9, supplement pp. 32-44
23c	Process limitations	Search/selection lineage and later technical audit limitations explicit.	Section 4.5, main p. 15; S2, supplement pp. 3-8; S9, supplement pp. 32-44
23d	Implications	Clinical-question separation and author-proposed reporting considerations.	Section 4.3, main p. 14; S8, supplement pp. 29-31
24a	Registration	PROSPERO CRD420261441918 Version 1.0 published 5 July 2026 verified.	Section 2.1, main pp. 2-3; S1, supplement p. 2
24b	Protocol access	Original registration PDF retained and compared with later operational methods. The PDF does not document a later registry amendment.	S1, supplement p. 2

Item	Topic	Status and explanation	Verified location
24c	Amendments	Protocol-to-methods differences, documentary sources and dates/status in S9; later choices are not labelled prospectively registered.	Section 2.1, main pp. 2-3; Section 2.3, main pp. 3-4; S1, supplement p. 2; S5, supplement pp. 13-14; S9, supplement pp. 32-44
25	Support	Present-study funding declaration reported.	Funding, main p. 16
26	Competing interests	Competing-interest declaration reported.	Conflicts of Interest, main p. 17
27	Data and code	Tables S1-S9, the separate S4 decision index, reporting checklists and Supplementary Data File 1 are provided for publication with the article. Supplementary_Data_File_1.zip includes coded analytic data, the searchable HTML ledger, compact audit/sampling data, dictionary, reproduction code and reference outputs. Original forms, detailed source-verification notes and retained historical search/deduplication/version records not included publicly are available on reasonable request from Jen-Tang Sun (femh83316@femh.org.tw). Copyrighted source PDFs and unpublished companion manuscripts are excluded from the public file; redistribution is not promised.	Data Availability Statement, main p. 16; Supplementary Materials and Reporting Checklists, main p. 16; S4, supplement p. 12; separate Supplementary_Data_File_1.zip
