# Supplementary material for: An Evidence Map of Pupillary Assessment in a Preselected Acute-Care Literature Archive
Source: Diagnostics (Basel). 2026 Nov 9;16(21):3698. doi: 10.3390/diagnostics16213698 (PMC13761962; doi:10.3390/diagnostics16213698)
Supplement: Supplementary file 1 [file diagnostics-16-03698-s001.zip › Supplementary_Data_File_1/S4_Searchable_Report_Ledger.html]

Searchable Report Decision Ledger 

# Searchable Report Decision Ledger

An Evidence Map of Pupillary Assessment in a Preselected Acute-Care Literature Archive. Supplementary Data File 1. This ledger contains final report-level codes, bibliographic identifiers and concise source locators for all 871 archive records. It is generated from the public analytic JSON. Full source narratives, completed forms and historical records are available on reasonable request from Jen-Tang Sun (femh83316@femh.org.tw); copyrighted source PDFs are not distributed here.

355 reports contribute to the core map. Categories for excluded and noncore records are retained for transparency, not counted as core evidence. “Not established” and “Unclear” are not evidence of absence. Source locators refer to the final preserved record, not necessarily the initial audit pass. This ledger does not establish independent cohorts or pooled patient totals.

Search Disposition AllIncludeExcludeQualitative onlyDuplicate

| Report ID | Bibliography | Disposition and code | Aetiology | Setting | Acquisition | Pupil-related primary endpoint | Design | Publication period | Source locators |
| --- | --- | --- | --- | --- | --- | --- | --- | --- | --- |
